# Multi-omics Analyses of Non-GM Tomato Scion Engrafted on GM Rootstocks

**DOI:** 10.14252/foodsafetyfscj.D-23-00005

**Published:** 2023-09-06

**Authors:** Takumi Ogawa, Kanae Kato, Harue Asuka, Yumi Sugioka, Tomofumi Mochizuki, Takumi Nishiuchi, Taira Miyahara, Hiroaki Kodama, Daisaku Ohta

**Affiliations:** 1 Graduate School of Agriculture, Osaka Metropolitan University, 1-1 Gakuen-cho, Naka-ku, Sakai, Osaka 599-8531, Japan; 2 Graduate School of Life and Environmental Sciences, Osaka Prefecture University, 1-1 Gakuen-cho, Naka-ku, Sakai, Osaka 599-8531, Japan; 3 Division of Life Science, Graduate School of Natural Science and Technology, Kanazawa University, Kakuma, Kanazawa, Ishikawa 920-1192, Japan; 4 Division of Integrated Omics Research, Bioscience Core Facility, Research Center for Experimental Modeling of Human Disease, Kanazawa University, 13-1 Takaramachi, Kanazawa, Ishikawa 920-8640, Japan; 5 Graduate School of Horticulture, Chiba University, 1-33 Yayoi-cho, Inage-ku, Chiba 263-8522, Japan

**Keywords:** new plant breeding technology, transgrafting, omics analysis, tomato, tobacco, luciferase

## Abstract

Grafting has been widely applied in agricultural production in order to utilize agriculturally valuable traits. The use of genetically modified (GM) plants for grafting with non-GM crops will soon be implemented to generate chimeric plants (transgrafting)*, and the non-GM edible portions thus obtained could fall outside of the current legal regulations. A number of metabolites and macromolecules are reciprocally exchanged between scion and rootstock, affecting the crop properties as food. Accordingly, the potential risks associated with grafting, particularly those related to transgrafting with GM plants, should be carefully evaluated based on scientific evidence. In this study, we prepared a hetero-transgraft line composed of non-GM tomato scion and GM-tobacco rootstock expressing firefly luciferase. We also prepared a homograft line (both rootstock and scion are from non-GM tomato) and a heterograft line (non-GM tobacco rootstock and non-GM tomato scion). The non-GM tomato fruits were harvested from these grafted lines and subjected to comprehensive characterization by multi-omics analysis. Proteomic analysis detected tobacco-derived proteins from both heterograft and hetero-transgraft lines, suggesting protein transfer from the tobacco rootstock to the tomato fruits. No allergenicity information is available for these two tobacco-derived proteins. The transcript levels of the genes encoding two allergenic tomato intrinsic proteins (Sola l 4.0101 and Sola l 4.0201) decreased in the heterograft and hetero-transgraft lines. Several differences were observed in the metabolic profiles, including α-tomatine and nicotine. The accumulation of tobacco-derived nicotine in the tomato fruits of both heterograft and hetero-transgraft lines indicated that the transfer of unfavorable metabolites from rootstock to scion should be assessed as a food safety concern. Further investigations are needed to clarify whether variable environmental conditions and growth periods may influence the qualities of the non-GM edible parts produced by such transgrafted plants.

## 1. Introduction

During the long history of the domestication process of wild plants, conventional breeding practices have contributed to the establishment of crop varieties with desirable agricultural traits through repeated generations of crossbreeding and selection. However, humanity now faces food supply challenges as a result of extreme climate conditions caused by global warming and major international conflicts, and finding the means to secure sustainable agricultural production with the aid of rapid and efficient crop breeding is urgently needed.

Advances in plant molecular biology are expected to provide critical clues to overcoming these unprecedented food security challenges by making possible the genetic modification of crops by inserting genes encoding desirable traits, such as high yield, improved tolerance to abiotic and biotic stresses, increased nutritional value, and reduced allergic properties. These genetically modified (GM) crops with introduced foreign DNA are subject not only to the Act on the Conservation of Biological Diversity through Regulations on the Use of Living Modified Organisms (the Cartagena Act) but are also under government regulations for the safety assessment of GM plants for use as foods and feeds^1^^)^. Furthermore, in recent years, several new plant breeding technologies (NPBTs) have been developed and implemented to facilitate the breeding of crop varieties, taking advantage of the increased accuracy of genome sequence modification and the reduced time and effort required to establish new varieties compared to conventional genetic modification techniques. NPBTs include genome editing technology, oligonucleotide-directed mutagenesis (ODM), RNA-dependent DNA methylation (RdDM), cisgenesis, intragenesis, grafting using GM rootstocks (transgrafting)*, agroinfiltration, and reverse breeding^2^^)^. Breeding based on genome editing technologies, especially CRISPR/Cas9 technology, has been rapidly attracting attention due to its versatility, simplicity, and speed^3^^)^. The most important and realistic benefit of NPBTs is the commercialization of crop varieties that do not contain foreign DNA, irrespective of the presence of transgenes during the intermediate breeding stages. Genome-edited crops have already been placed on the market, and there are dissenting opinions regarding their safety^4^^,^^5^^,^^6^^,^^7^^)^. The fact that inserted foreign DNA is no longer present makes it difficult to distinguish between crops produced by natural mutation, chemical mutation, or genome editing, and they are indistinguishable from conventional plant breeding products.

In this study, we focused on the food safety assessment of transgrafted crops* composed of GM and non-GM (not genetically modified) plant portions. In general, grafted plants continue to grow as chimera, the above-ground part of which is attached to a stem, root, etc., derived from another plant^8^^,^^9^^)^. In many cases, by grafting an above-ground portion (scion) onto a root-bearing portion with useful traits such as disease resistance or environmental tolerance (rootstock), it is possible to stably harvest from the scion with high-value traits^10^^,^^11^^)^, including those derived from GM plants. In the transgrafted plants, non-GM edible portions are physically separated from the GM-portion: transgrafting could circumvent a number of current regulations on GM crops^12^^)^. For example, the transgrafting of a non-GM scion onto a soil-borne disease-resistant GM rootstock would not be subject to regulations concerning GM crops, because fruits borne on non-GM scions are not regarded as GM. By applying transgrafting to root vegetables such as potato, the productivity of the non-GM edible tubers of conventional cultivars can be improved by GM scions. It is also possible, by using GM rootstock that produces small RNA, to modify the properties of the non-GM scion without compromising useful agricultural traits^13^^)^.

Grafting is not a universal technology. Most plant species can graft on themselves, and some plants can graft on related species, while grafting between more distantly related species is usually challenging. However, Notaguchi et al. (2020) presented the possibility of successful grafting between a wide range of different plant species using a single useful genetically modified species or a previously unavailable wild species^14^^)^. By using *Nicotiana* stem as an inter-scion, they demonstrated that intrafamily grafting between a tomato scion and rootstocks from either Asteraceae or *Arabidopsis thaliana* was established through cell wall remodeling by β-1,4-glucanase activity and successfully produced fruits from the tomato scion^14^^)^.

Thus, interspecies grafting could be broadly implemented to expand food production ability by allowing harvesting from non-GM portions that do not contain foreign DNA while incorporating the benefits of GM traits and/or useful agricultural traits from wild species that have thus far been unutilized. However, it is necessary to carefully evaluate how the harvests from the non-GM portions for food could have been affected by the GM portion or wild species. It is well known that metabolites, small RNA, and peptides are mobile between rootstock and scion through the vasculature^15^^,^^16^^,^^17^^)^. Such reciprocal mobilizations of biological molecules within grafted plants have not been broadly examined in the context of food safety assessment. Multi-omics approaches are expected to provide highly valuable contributions to address such food safety concerns related to newly developed crops produced by NPBT-based breeding processes^18^^,^^19^^)^.

To clarify the possible effects of the GM portions on the non-GM portions, we have analyzed transgrafted Solanaceae crops by multi-omics approaches^20^^,^^21^^,^^22^^)^. There was no marked difference in terms of the transcriptomic and metabolomic properties of the non-GM tomato scions grafted on GM tomato rootstocks expressing a recombinant β-glucuronidase protein^20^^)^. Likewise, in the non-GM tobacco leaves on an RdDM-inducing rootstock, the GM portions had no significant impact on the food safety of the non-GM portions^21^^)^. Miyahara et al. (2023) also demonstrated that there were no significant concerns regarding food safety in the tubers harvested from non-GM rootstock transgrafted to GM potato scion expressing FLOWERING LOCUS T (FT) peptide^22^^)^. These results indicated that there was no apparent food safety concern in the non-GM part as a result of the transgrafting of the GM grafting partners.

In this study, we investigated the possible effects of rootstock from GM-tobacco plants expressing firefly luciferase (Luc) on the properties of non-GM tomato scions. Rootstocks from Luc-expressing *Nicotiana tabacum* cv. SR1^21^^)^ were transgrafted onto non-GM tomato, *Solanum lycopersicum* cv. Micro-Tom. After cultivation, the tomato fruits from the non-GM portions were harvested and subjected to multi-omics analyses. As a food safety assessment, we also evaluated the nutritional properties, steroidal alkaloid levels, and build-up of nicotine in the tomato fruits.

## 2. Materials and Methods

### 2.1 Plant Materials

Tomato (*S. lycopersicum* L. cv. Micro-Tom), tobacco (*N. tabacum* L. cv. SR1), and a transgenic tobacco line expressing Luc were used for grafting. Seeds were surface-sterilized, washed three times with sterile distilled water, and germinated on a half-strength Murashige−Skoog medium with 0.7% agar under a long-day photoperiod (16 h light/8 h dark) at 25°C. After 2 weeks, seedlings were transplanted to plastic pots containing potting soil (Golden Granular Potting Soil; Iris Ohyama, Miyagi, Japan) and were grown under a long-day photoperiod (16 h light/8 h dark) at 25°C. Three-week-old tomato scions (non-GM) were grafted on 3-week-old tomato rootstocks (non-GM), 8-week-old non-transgenic (non-GM) tobacco rootstocks, and 8-week-old Luc-expressing tobacco rootstocks. These grafted plants were designated as MT/MT (homograft), Nt/MT (heterograft), and NtLuc/MT (hetero-transgraft), respectively (**Table 1**). After grafting, plants were grown under a long-day photoperiod (16 h light/8 h dark) at 25°C. Axillary buds were removed from each grafted plant once a week during the growth.

**Table 1.** List of grafting combinations.

Grafted plant line	Homograft	Heterograft	Hetero-transgraft
	(MT/MT)	(Nt/MT)	(NtLuc/MT)
Rootstock	non-GM tomato*	non-GM tobacco**	GM-tobacco***
Scion#	non-GM tomato	non-GM tomato	non-GM tomato

# All grafted plants were produced using non-GM tomato scion.

* *Solanum lycopersicum* L. cultivar Micro-Tom

**Nicotiana tabacum L. cultivar SR1

**Nicotiana tabacum L. cultivar SR1 expressing a firefly luciferase

### 2.2 Sampling of Tomato Fruits for Multi-omics Analyses

Tomato fruits that reached the ripening stage of 10 days after breaker (DAB) were sequentially harvested from each grafted plant from 11 to 22 weeks after grafting. After measuring the fresh weight (FW), each fruit was divided into four equal parts, frozen in liquid nitrogen, and kept in a −80°C deep freezer until use. Five fruits of average FW were selected from each grafted line, and one of the four equal portions of individual fruits was used for either transcriptomic, proteomic, or metabolomic analyses.

### 2.3 Transcriptomic Analysis of Tomato Fruits

Total RNA was extracted from tomato fruit samples using a Plant Total RNA Mini Kit (Favorgen Biotech Crop., Taiwan). We outsourced RNA library construction and mRNA sequencing to Eurofins Genomics (Tokyo, Japan). mRNA was purified as poly(A)+ RNA, and paired-end 150 bp sequencing data was generated using a NovaSeq 6000 platform (Illumina, San Diego, USA) (BioProject ID: PRJDB15279, RUN ID: DRR443119-30). Adapter sequences were trimmed, and low-quality reads containing poly-N sequences and/or those that were less than 50 bp in length were discarded using fastp (version 0.20.1). After trimming, read data were aligned to the transcriptome dataset of *S. lycopersicum* (ITAG4.0_cDNA.fasta) and *N. tabacum* (Nitab-v4.5_cDNA_Edwards2017.fasta) registered at the Sol Genomics Network (https://solgenomics.net/) using Bowtie2 version 2.4.4. RSEM (version 1.3.3) was used to calculate gene expression levels, which were expressed as transcripts per million (TPM). Hierarchical cluster analysis (Ward’s method) and the identification of differentially expressed genes (DEGs) were performed using edgeR (version 3.34.1) and R (version 4.1.0). A Venn diagram was created using Bioinformatics & Evolutionary Genomics (https://bioinformatics.psb.ugent.be/webtools/Venn/). An investigation of the transfer of the luciferase mRNA (Uniprot Accession: P08659) to scions was confirmed by alignment of the NtLuc/MT_1–4 read data in the same way as above. The tomato allergen gene transcripts (Sola l 1–4 and 6, 7) from Allergen Online (http://www.allergenonline.org/) were investigated.

### 2.4 Proteomic Analysis of Tomato Fruits

For each gram of tomato fruits tissue, we added 10 g of 7 M urea and mashed the resulting mixture in a mortar and pestle. The homogenate was then placed in a tube and kept at room temperature for 30 min. This was then sonicated four times at 1 s intervals before being centrifuged at 1,300 rpm for 30 min at 20°C. Next, 250 µL of the centrifuged supernatant was transferred to a new tube, to which three times its volume in acetone chilled to −20°C was added. After vortexing, tubes were stored at −20°C overnight. Two centrifugations were then performed to completely remove the acetone; each centrifugation was 15,000 rpm for 15 min at 4°C. The resulting precipitants were then air-dried for 5 min. Finally, 30 µL of 7 M urea was added to completely dissolve the precipitate, and the protein content was quantified using a Qubit Protein Assay Kit (Thermo Fisher Scientific, Waltham, USA). To conduct further analyses, all protein samples (10 µg) were adjusted to a final volume of 10 µL in 6 M urea and 50 mM triethylammonium bicarbonate (TEAB, pH 8.5). These proteins were then reduced, alkylated, and digested by trypsin. The trypsin-digested peptides were purified and separated using a liquid chromatography (LC) system (EASY-nLC 1200; Thermo Fisher Scientific). The peptide ions were detected using a mass spectrometer (MS; Orbitrap QE plus MS; Thermo Fisher Scientific). Tandem mass spectral database searches were then carried out using SEQUEST HT search algorithms implemented in Proteome Discoverer (PD) 2.2 (Version 2.2.0.388; Thermo Fisher Scientific) to find query sequences in the tomato protein data file (ITAG4.0_proteins.fasta) and tobacco protein data file (Nitab-v4.5_proteins_Edwards2017.fasta), which is registered on the Sol Genomics Network. Label-free quantification was also performed with PD 2.2 using precursor ion quantifier nodes. When two or more different detected peptide fragments were completely matched to a single tobacco protein, we considered the tobacco protein to be present in the tomato fruit. Abundance normalization was performed using the total peptide amount mode. The biological processes and molecular functions of the proteins whose accumulations were found to differ between the two groups were further investigated using the DAVID Bioinformatics Resource^23^^)^ (https://david.ncifcrf.gov/tools.jsp, 2022 update).

### 2.5 Metabolomic Analysis of Tomato Fruits

The tomato fruits samples were subjected to metabolomic analysis using a LC-MS system. Untargeted metabolome analyses of tomato fruits samples were conducted according to the previously described method^22^^)^ using a Q Exactive mass spectrometer (Thermo Fisher Scientific) connected to an UltiMate 3000 Rapid Separation LC system (Thermo Fisher Scientific). Briefly, a methanol:water (4:1, v/v) extract of tomato fruits samples was filtrated through MonoSpin C18 columns (GL Sciences, Tokyo, Japan), and the filtrate (2-μl) was subjected to LC-MS analysis. Chromatographic separation was carried out in an InterSustain AQ-C18 column (2.1 mm × 150 mm, 3 μm particle size, GL Sciences). The mobile phase was 0.1% (v/v) formic acid in water (A) and acetonitrile (B). The gradient program was set as follows: 2% B, constant for 3 min, followed by an increase to 98% B within 30 min, kept constant for 5 min, and reduced to 2% B in 0.1 min. The post-run equilibrium time was 4.9 min. The flow rate was kept constant at 0.2 ml min^−1^. The column oven was kept at 40°C. Mass spectrometry was performed using a Q Exactive mass spectrometer. The instrument was set to operate in the ESI positive ion mode. All spectra were acquired in the range of *m*/*z* 80–1200. Tandem mass spectrometry was performed by collision-induced dissociation for the ten most intense ions of the full mass scan. Dynamic exclusion was set at 20 s. The raw data file was converted to the mzXML file format using ProteoWizard’s MSConvertGUI software (http://proteowizard.sourceforge.net). PowerGetBatch software^24^^)^ was used for peak detection, characterization, and alignment. The peaks were annotated using the Unique Connectivity of UnCharged compound (UC2) database^25^^)^ and the ExactMassDB-HR2 (EX-HR2) database at the MFSearcher program^26^^)^ with the predicted mass values of the original molecules at 5 ppm mass tolerance. The compound records of the UC2 database include the database records of the KNApSAcK^27^^)^ and the Human Metabolome Database (HMDB)^28^^)^.

### 2.6 Quantification of α-Tomatine and Nicotine in Tomato Fruits

Determination of α-tomatine content in tomato fruits was performed by the method described by Iijima et al. (2013)^29^^)^ using an external calibration curve for α-tomatine (MedChemExpress, Monmouth Junction, USA). Determination of nicotine content in tomato fruits was performed by the method described by Liu et al. (2013)^30^^)^ with a slight modification using (±)-nicotine-methyl-*d*_3_ (CDN Isotopes, Quebec, Canada) as an internal standard. Analysis was carried out by multiple reaction monitoring (MRM) mode using mass transition of *m*/*z* 166.20 > 133.05 at a collision energy of −35 V for the labeled nicotine and mass transition of *m*/*z* 163.20 > 130.05 at a collision energy of −35 V for the non-labeled nicotine.

### 2.7 Nutrient Composition Analysis

Macro nutrients (i.e., moisture, protein, fat, ash, and carbohydrate contents) were evaluated by Japan Food Research Laboratories (Tokyo, Japan). Moisture content was analyzed by the decompression drying method. Protein content was analyzed using the combustion method and quantified as 6.25 times the N content. Crude fat was determined by acid hydrolysis and ether extraction method. Ash content was determined by the direct ash method. Carbohydrate content was calculated by subtracting measured moisture, protein, fat, and ash from the total weight. Energy was calculated using the following conversion factor: four for protein and carbohydrate and nine for fat.

### 2.8 Statistical Analyses

Principal component analysis (PCA), hierarchical clustering analysis (HCA), analysis of variance (ANOVA) and Tukey’s honestly significant difference (HSD) test was performed using MetaboAnalyst^31^^)^ ver. 5.0, a web tool for metabolomics data analysis (https://www.metaboanalyst.ca/home.xhtml). The data filtering option “mean intensity value” was selected to identify and remove ion peaks displaying ion intensities close to baseline or detection limit. Auto scaling was selected as the data standardization method for PCA. Box plots were generated using the BoxPlotR^32^^)^, a web tool for generation of box plot (http://boxplot.tyerslab.com).

## 3. Results

### 3.1 Grafted Plants

**Fig. 1.**
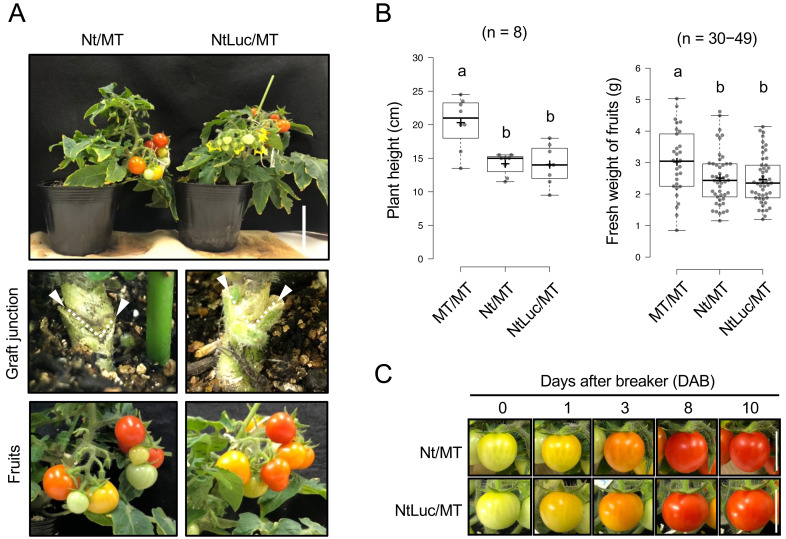
Phenotype of non-GM *S. lycopersicum* cv. Micro-Tom scions grafted on non-GM *N. tabacum* rootstock (Nt/MT), and Luc-expressing *N. tabacum* rootstock (NtLuc/MT). (A) Aerial part, graft junction, and fruits of Nt/MT (heterograft) and NtLuc/MT (hetero-transgraft). White arrowhead and dashed line indicate the junction between scion and rootstock. Scale bar indicates 50 mm. (B) Plant height and fruits fresh weight of Nt/MT and NtLuc-MT. As a comparison, non-transgenic *S. lycopersicum* cv. Micro-Tom scions grafted on non-transgenic *S. lycopersicum* cv. Micro-Tom rootstock (homograft; MT/MT) is shown. Tukey’s honestly significant difference test was applied to the three groups. Different letters above the box plot indicate statistical differences at *p* < 0.05. (C) Fruit ripening stages of Nt/MT and NtLuc/MT. Fruits at the ripening stages of 0, 1, 3, 8, and 10 days after breaker (DAB) are shown. Scale bars indicates 20 mm.

Grafting has been widely applied to improve the productivity of vegetable crops, including the Solanaceae and Cucurbitaceae family plants. In this study, we generated heterografted plants composed of tobacco rootstocks and tomato scions (**Table 1**, **Fig. 1A**). Throughout this study, non-GM tomato (*S. lycopersicum* cv. Micro-Tom) was used as the scion onto rootstocks of either non-GM or Luc-expressing (GM) tobacco plants. These heterografted plants with non-GM tomato scions were designated as Nt/MT (non-GM tobacco/Micro-Tom: hetrograft line) or NtLuc/MT (GM tobacco/Micro-Tom: hetero-transgraft line). Compared to the MT/MT (Micro-Tom/Micro-Tom: homograft line) plants generated as a control for the grafting experiment, the growth of the tomato scion parts (height and fruit weight) was significantly reduced in the heterograft (Nt/MT) and hetero-transgraft (NtLuc/MT) lines with tobacco rootstocks (**Fig. 1B**). There was no difference in the growth and fruits of the above-ground part between the heterograft line and the hetero-transgraft line (Figs. 1B and1C). It has been reported that the use of tobacco as rootstocks could endow the tomato scions benefits such as growth promotion, early flowering, and fruits weight increase^33^^,^^34^^)^. In this study, the use of tobacco as the rootstock did not afford such growth promotion of the scion parts. Further analyses were conducted using the fruits of 10 DAB from the grafted plants from 11 to 22 weeks after grafting (**Fig. 1C**).

### 3.2 Transcriptomic Profiles of Tomato Fruits from Grafted Plants

Alignment of the read data of each sample to tomato transcriptome data (ITAG4.0_cDNA.fasta) and tobacco transcriptome data (Nitab-v4.5_cDNA_Edwards2017.fasta) resulted in alignment rates of approximately 80% and 20%, respectively (**Supplementary Table S1**). Hierarchical cluster analysis (HCA) of the gene expression data obtained from each of the two alignment results showed that no clusters were formed in a particular group of grafting combinations (**Supplementary Fig. S1**). *Luc* gene sequences were not found in NtLuc/MT_1–4 read data.

**Fig. 2.**
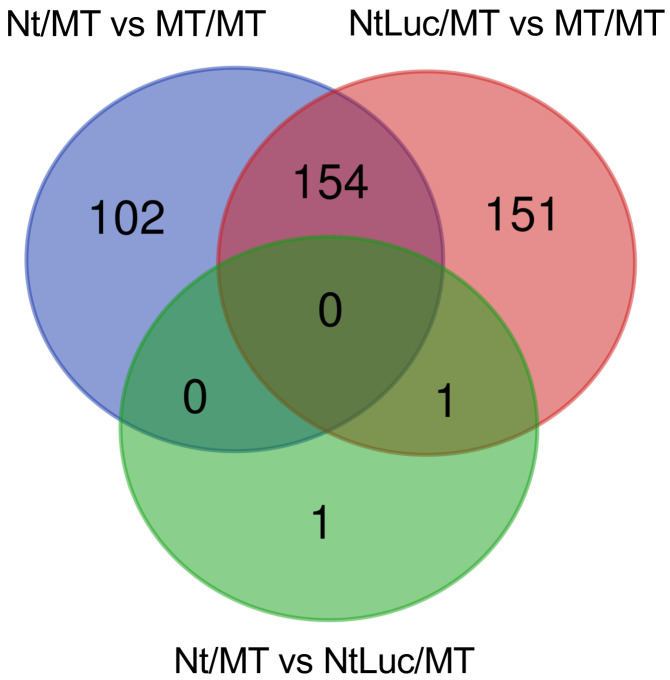
Venn diagram of DEGs from three different grafting combinations. Blue, red, and green circles represent the DEGs revealed from the comparisons of Nt/MT (heterograft) and MT/MT (homograft), NtLuc/MT (hetero-transgraft) and MT/MT, and Nt/MT and NtLuc/MT, respectively. Each digit in the diagram indicates the number of DEGs.

The gene expression data obtained by alignment to the tomato transcriptome data (ITAG4.0_cDNA.fasta) were used to investigate the differential expression genes (DEGs). As a result, 256 genes were detected as DEGs in the heterograft line (Nt/MT) and the homograft line (MT/MT) with P_FDR_ < 0.05. Of these, 122 genes were up-regulated, and 134 genes were down-regulated in the heterograft line (**Supplementary Table S2**). In the comparison between the hetero-transgraft line (NtLuc/MT) and the homograft line, 306 genes were detected as DEGs. Of these, 165 genes were up-regulated, and 141 genes were down-regulated in the hetero-transgraft line (**Supplementary Table S3**). In the comparison between the heterograft line and the hetero-transgraft line, two genes were detected as DEGs, and both genes were down-regulated in the hetero-transgraft line (**Supplementary Table S4**). There were 154 genes commonly found as DEGs in both heterograft lines (Nt/MT and NtLuc/MT) and the homograft line (Fig. 2), and 83 genes and 71 genes were up-regulated and down-regulated in similar manners in both heterograft lines, respectively (**Supplementary Table S5**). Gene ontology (GO) analysis of these DEGs indicated that genes classified as biological rhythms and stress response were variable. One gene (Solyc00g500041.1) encoding NAD(P)H-quinone oxidoreductase subunit 1 was identified as a DEG in the comparison among the grafted lines; its expression level in the hetero-transgraft line was lower than those in the transgraft and homograft lines (**Supplementary Table S6**). In the comparison with the homograft line, genes encoding allergenic proteins Sola l 4.0101 (Solyc09g090990.2) and Sola l 4.0201 (Solyc09g090980.3) were down-regulated in the hetero-transgraft line (**Supplementary Tables S3 and S5**). Sola l 4.0101 gene expression was also down-regulated in the heterograft line (**Supplementary Table S5**).

### 3.3 Proteomic Profiles of Tomato Fruits from Grafted Plants

**Fig. 3.**
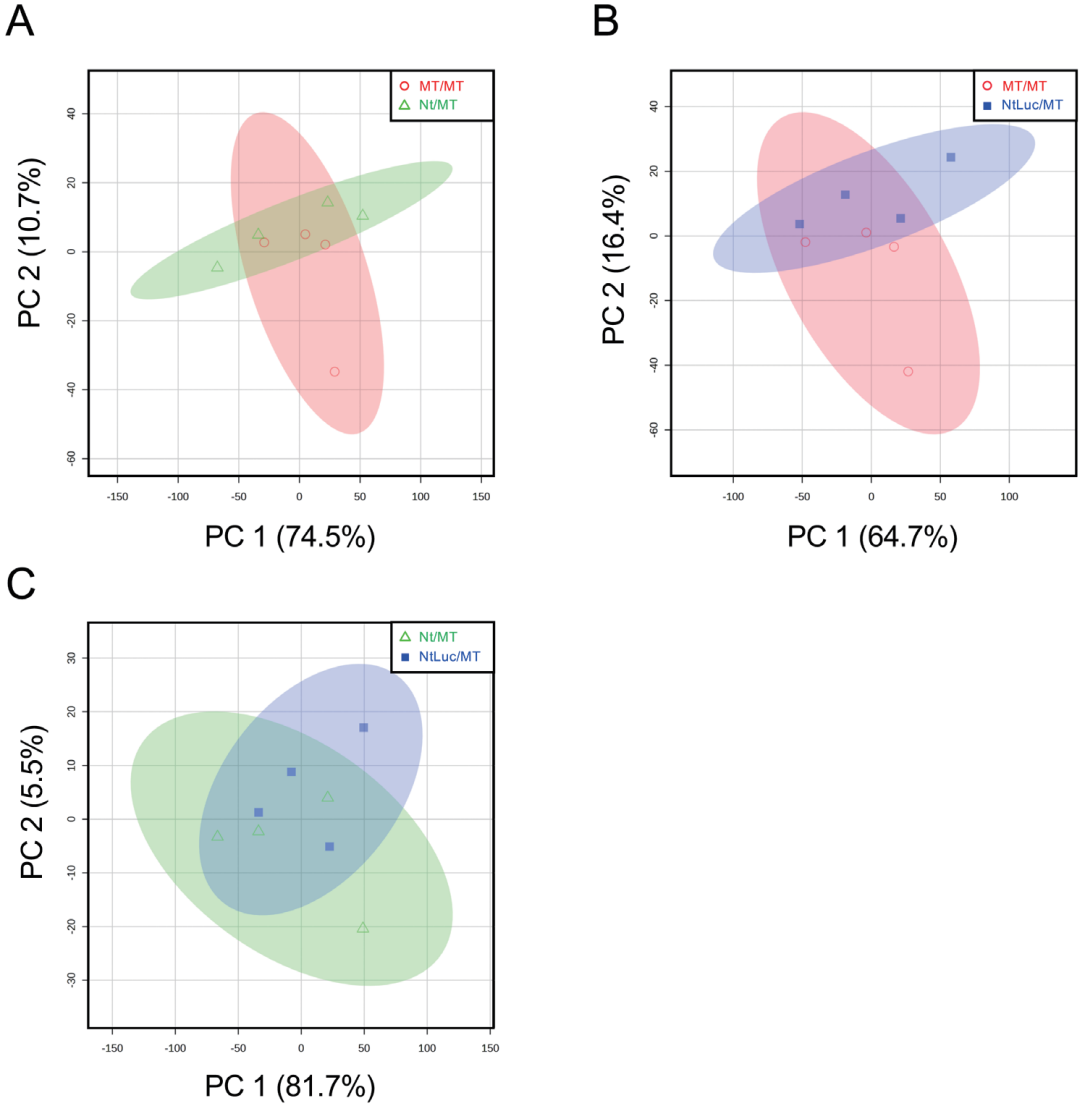
PCA of proteomic data using protein abundances from MT/MT (homograft), Nt/MT (heterograft), and NtLuc/MT (hetero-transgraft). (A) PCA of proteomic data in Nt/MT and MT/MT. (B) PCA of proteomic data in NtLuc/MT and MT/MT. (C) PCA of proteomic data in NtLuc/MT and Nt/MT. Percentage values in parentheses are the respective contribution ratios. The 95% confidence regions of MT/MT, Nt/MT, and NtLuc/MT are shown by ovals filled with light red, light green, and light blue, respectively.

**Fig. 4.**
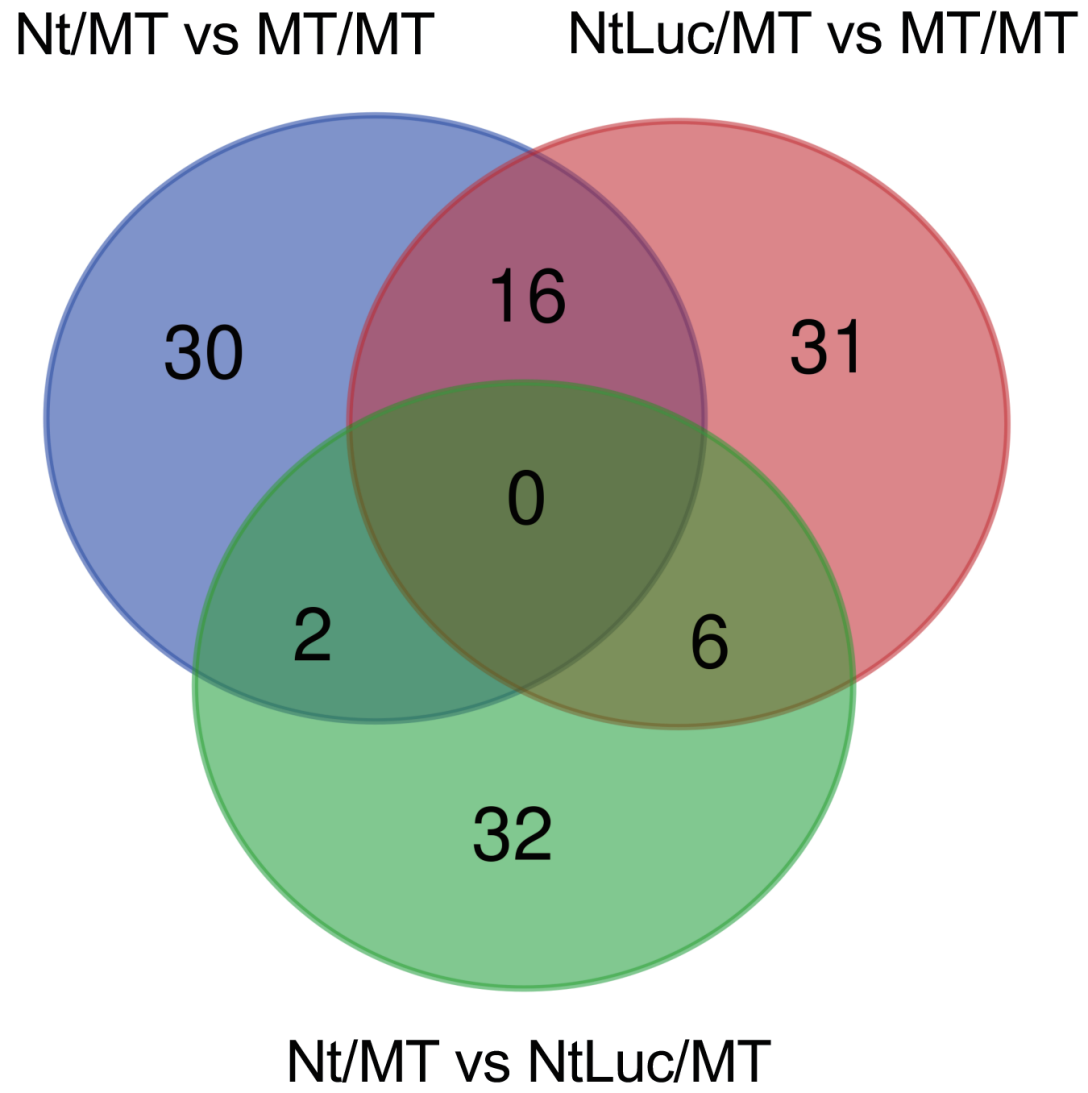
Venn diagram of differentially abundant proteins from three different grafting combinations. The blue, red, and green circles represent differentially abundant proteins obtained from the comparisons of Nt/MT (heterograft) and MT/MT (homograft), NtLuc/MT (hetero-transgraft) and MT/MT, and Nt/MT and NtLuc/MT, respectively. Digits in the circles indicate the numbers of proteins found to differ in abundance.

**Fig. 5.**
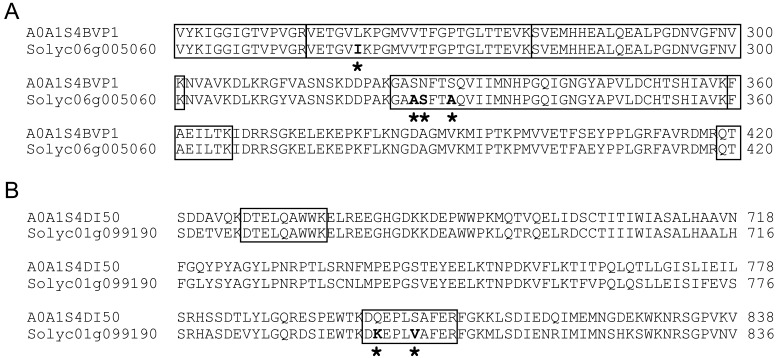
Locations of peptide fragments within tobacco proteins detected by proteome analysis and their partial alignment in tomato homologous proteins. (A) Partial alignment of tobacco elongation factor 1-alpha (A0A1S4BVP1: *Nicotiana tabacum*) and its tomato homologue (Solyc06g005060: *Solanum lycopersicum*). (B) Partial alignment of tobacco lipoxygenase (A0A1S4DI50: *Nicotiana tabacum*) and its tomato homologue (Solyc01g099190: *Solanum lycopersicum*). The boxed lines indicate peptide fragments detected from proteome analysis. Asterisks and bold letters indicate different amino acid residues in the tobacco and tomato sequences.

The detected peptide fragments were aligned using the tomato protein data (ITAG4.0_proteins.fasta) and tobacco protein data (Nitab-v4.5_proteins_Edwards2017.fasta) as query sequences. The results showed that 4,229 proteins were detected as tomato- or tobacco-derived proteins, but there was no evidence that Luc proteins were present. PCA showed no clear cluster separation between any grafting combinations (**Fig. 3**). Next, proteins with variable abundance were investigated through comparisons among the three grafted lines (MT/MT, Nt/MT, and NtLuc/MT). We chose the proteins for further analysis according to the following criteria: peptide sequences were detected at two or more locations in the query sequence, the abundance ratio *p*-value < 0.05 was met, and there was a twofold or greater difference in the abundance ratio value. The results demonstrated that the 48 proteins were differentially accumulated between the heterograft line (Nt/MT) and the homograft line (MT/MT). Similarly, 53 proteins that exhibited different abundances were listed in the comparison between the hetero-transgraft line (NtLuc/MT) and the homograft line, and 40 proteins were found to be differentially accumulated in the heterograft line and the hetero-transgraft line (**Supplementary Tables S7–S9**). A Venn diagram was generated to examine the proteins commonly found in the comparisons among the grafted plant lines (**Fig. 4**). As a result, it was found that 16 proteins commonly differed in abundances between the heterograft lines (Nt/MT and NtLuc/MT) and the homograft line (**Fig. 4**, **Supplementary Table S10**). Of these, three proteins accumulated at higher levels in both heterograft lines, and 12 exhibited decreased accumulation levels in the homograft line. GO analysis indicated that the two proteins with decreased accumulation levels in the heterograft lines were involved in lipid transport (A0A3Q7G5J4, A0A3Q7EII7). We detected six proteins that commonly varied in abundance from the comparison of the hetero-transgraft line and other lines (**Supplementary Table S11**). Of these, three proteins accumulated at higher levels, and another three proteins were found to be present at lower levels, in the hetero-transgraft line. GO analysis indicated that the accumulation levels of two proteins, K4D1U9 related to lipid transport and A0A3Q7IJL2 containing α-amylase inhibitor domain, increased and decreased in the hetero-transgraft line, respectively. In addition, two proteins were identified to have accumulated at higher levels in the heterograft line compared to the other lines (**Supplementary Table S12**). Elongation factor 1-alpha (A0A1S4BVP1) detected in the tomato fruits from the hetero-transgraft line was determined to be a tobacco protein with tobacco-specific amino acid sequences (**Fig. 5**). Lipoxygenase (A0A1S4DI50) was also detected as a tobacco-derived protein in the tomato fruits of both the heterograft and the hetero-transgraft lines. Other proteins annotated as tobacco-derived proteins had amino acid sequences consistent with the corresponding tomato proteins, suggesting that tomato proteins were mistakenly annotated. None of the allergenic proteins detected showed more than twofold variations in accumulation levels between the heterograft lines (Nt/MT and NtLuc/MT) and the homograft line. The variable expression levels of three genes in the heterograft line (**Supplementary Table S13**) and eight genes in the hetero-transgraft line (**Supplementary Table S14**) coincided with the variable abundance levels revealed by the proteomic analyses. Many genes were down-regulated in the heterograft lines (Nt/MT and NtLuc/MT) compared to the homograft line, and environmental stress-related genes, such as pathogenesis-related genes and chitinases, were down-regulated in the heterograft lines (Nt/MT and NtLuc/MT).

### 3.4 Metabolomic Profiles of Tomato Fruits from Grafted Plants

**Fig. 6.**
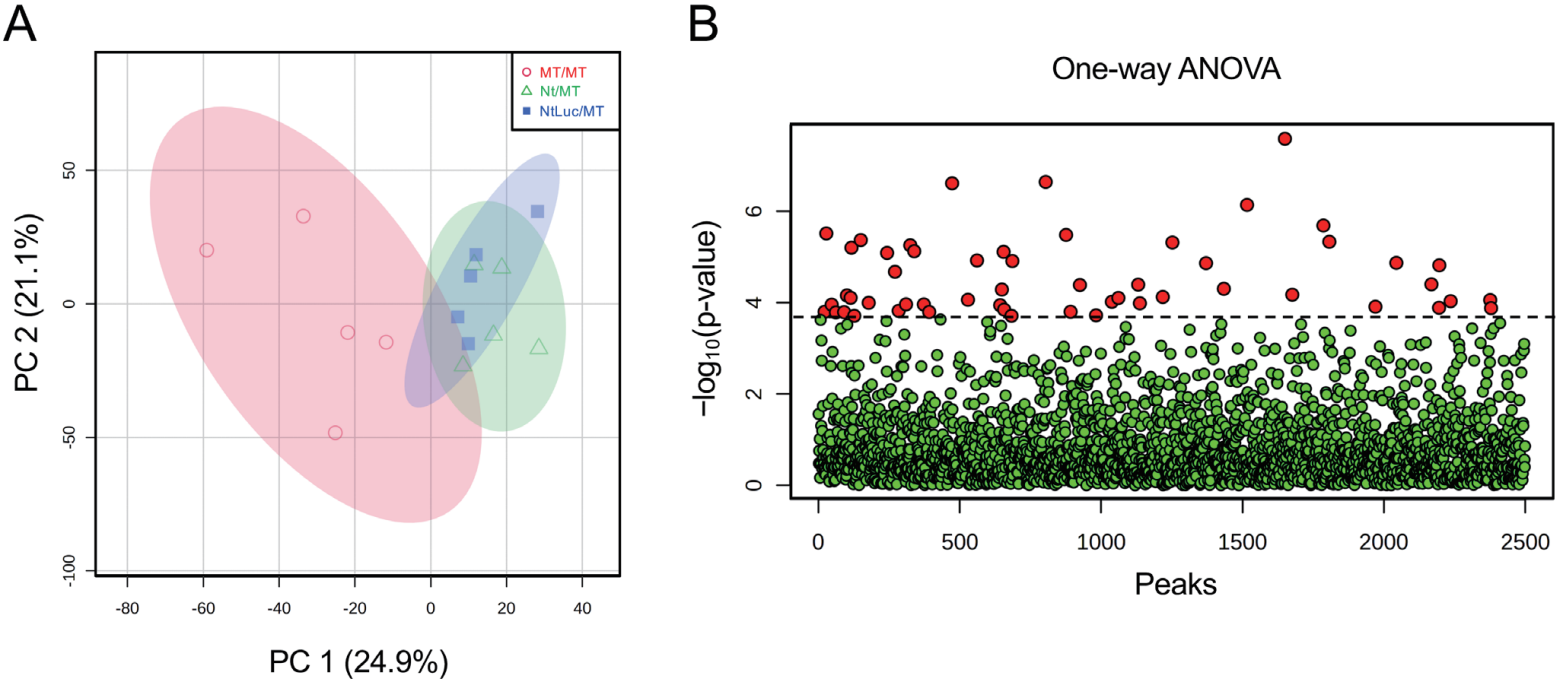
Metabolomic data analyses of fruits from MT/MT (homograft), Nt/MT (heterograft), and NtLuc/MT (hetero-transgraft). (A) PCA of metabolomic data of fruits from MT/MT, Nt/MT, and NtLuc/MT. A two-dimensional score plot graph composed of the combination of PC1 and PC2 is shown. Each plot represents an individual sample (n = 5). Percentage values in parentheses are the respective contribution ratios. The 95% confidence regions of MT/MT, Nt/MT, and NtLuc/MT are shown by ovals filled with light red, light green, and light blue, respectively. (B) One-way analysis of variance of metabolomic data from fruits from MT/MT, Nt/MT, and NtLuc/MT. Fifty-four ion peaks are identified as significant metabolites (P_FDR_ < 0.05) and shown as red circles. The ion detection information and annotation of the 54 ion peaks are shown in **Supplementary Tables S16 and S17**, respectively.

**Figure 6A** shows the PCA results to compare metabolic profiles among grafted plant lines. The PCA was performed using the ion intensity values of 2,499 ion peaks obtained by the high-resolution LC-MS analysis. From the PC 1 (contribution ratio: 24.9%), it is obvious that the metabolome profile of the homograft line (MT/MT) was clearly differentiated from those of the heterograft lines (Nt/MT and NtLuc/MT). The metabolome profiles of the heterograft lines (Nt/MT and NtLuc/MT) were closely plotted on the PCA diagram. These results indicated that the differentiation of the metabolome profiles of the heterograft lines (Nt/MT and NtLuc/MT) from the homograft line was caused by the use of the tobacco rootstocks irrespective of non-GM or GM. To obtain actual metabolite information from the PCA results, the top 10 and bottom 10 ions were listed according to each contribution for PC1 loading, and putative molecular formulas were assigned to these ions based on the high-resolution mass spectral data (**Supplementary Table S15**). It was found that ion peaks of ID 337 and ID 565 were attributable to nicotine and cotinine, the tobacco metabolites, respectively. Other ion peaks were not clarified by the database search using the MFSearcher^26^^)^ that searches across the ExactMassDB-HR2 database (http://webs2.kazusa.or.jp/mfsearcher/exmassdb-hr2/) and the UC2 database^25^^)^. The 2,499 ion peaks subjected to the PCA (**Fig. 6A**) were further analyzed by one-way ANOVA, and 54 ion peaks were identified to be present at significantly different abundances (P_FDR_ < 0.05) among the grafted plant lines (**Fig. 6B**, **Supplementary Tables S16 and S17**). These 54 ion peaks were then used for HCA (**Supplementary Fig. S2**). The homograft line and the heterograft lines (Nt/MT and NtLuc/MT) formed clearly different clusters (**Supplementary Fig. S2**). The ion peaks observed at high intensities in the fruits of the heterograft line were also detected at high intensities in the fruits of the hetero-transgraft line, and the ions detected at low intensities were common between the heterograft line and the hetero-transgraft line (**Supplementary Table S16**). Molecular formulas were deduced for these 54 ion peaks, and two ions (peak ID 337 and peak ID 565) were annotated as nicotine and cotinine (**Supplementary Table S17**), respectively. Of these 54 ion peaks, 52 showed significant differences in the mean ion intensity values between the homograft line and the heterograft lines (Nt/MT and NtLuc/MT). In the heterograft lines (Nt/MT and NtLuc/MT), only a single ion (peak ID 344) was detected with statistically significant difference in the average ion intensity values. The ion intensity value of peak ID 344 from the hetero-transgraft line was lower than the heterograft line, and it was higher than the homograft line (**Supplementary Fig. S3**).

### 3.5 α-Tomatine and Nicotine Contents of Tomato Fruits

**Fig. 7.**
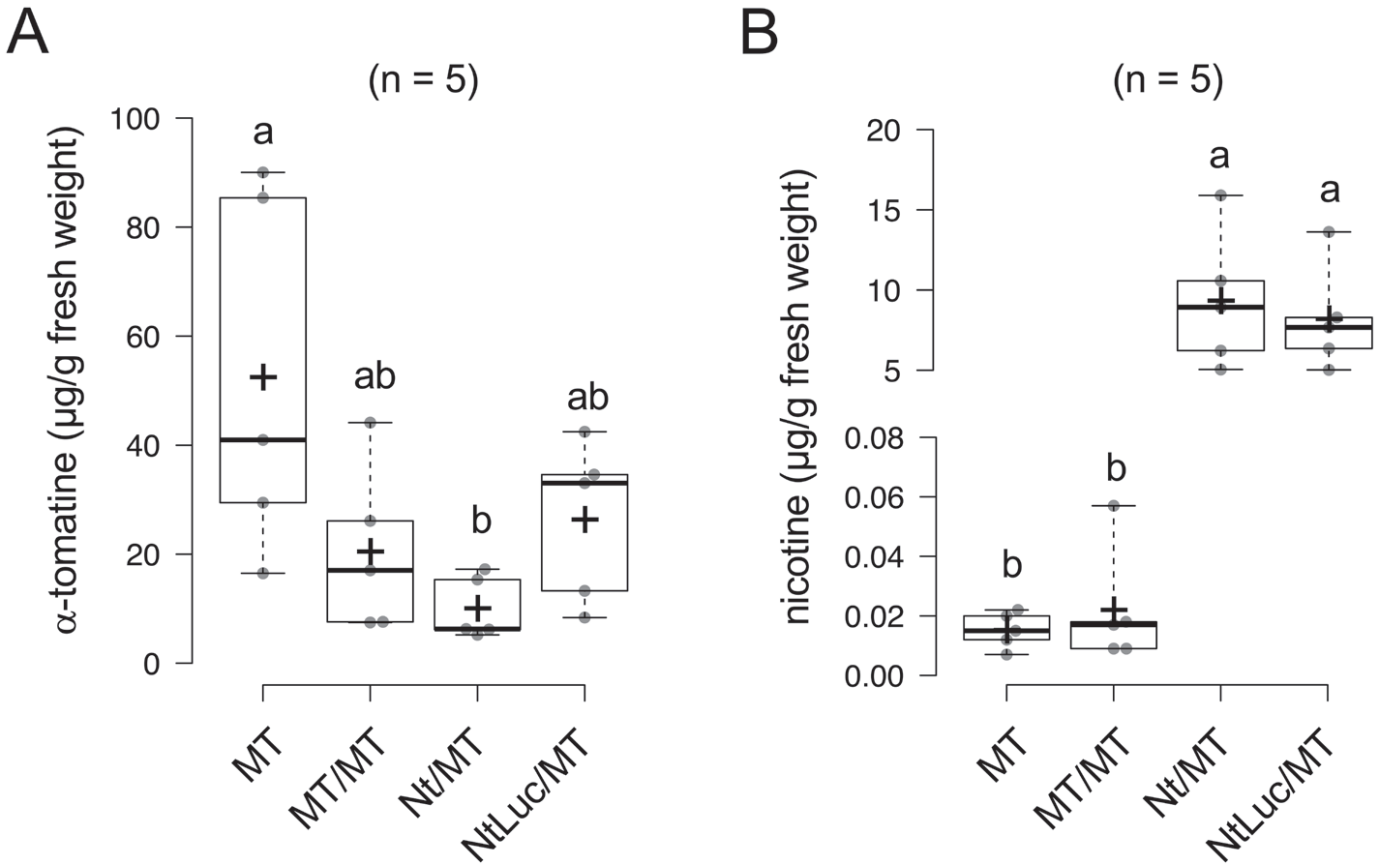
Comparisons of α-tomatine and nicotine contents in fruits. Contents of (A) α-tomatine and (B) nicotine in fruits from non-grafted non-transgenic *Solanum lycopersicum* cv. Micro-Tom (MT), MT/MT (homograft), Nt/MT (heterograft), and NtLuc/MT (hetero-transgraft) are shown. Tukey’s honestly significant difference test was applied to the four groups. Different letters above the box plot indicate statistical differences at *p* < 0.05.

**Figure 7** shows the effects of transgrafting on the α-tomatine and nicotine levels in the tomato scions. The α-tomatine contents in the tomato fruits of the grafted plants (MT/MT, Nt/MT, and NtLuc/MT) were lower than those in the non-grafted tomato plants (MT). There were no significant differences in the α-tomatine contents among the grafted plants, indicating that the use of the GM tobacco rootstock (NtLuc) did not affect the α-tomatine biosynthesis in the tomato scion parts (**Fig. 7A**). The nicotine contents in the tomato fruits were higher in the heterografted lines (Nt/MT, and NtLuc/MT) with the tobacco rootstocks regardless of non-GM or GM, indicating that nicotine transport from the tobacco rootstocks was not affected by the Luc protein expression. The nicotine contents in the tomato fruits were around 10 μg/g FW (**Fig. 7B**). No significant difference was observed between the heterograft lines (Nt/MT and NtLuc/MT).

### 3.6 Comparison of Nutrient Composition

The content of basic food components (moisture, protein, fat, ash, carbohydrate, and energy) in the fruit was compared among the three grafted lines. Several fruit samples were randomly selected from each grafted line and pooled to ensure the necessary weight (30 g FW) for analysis. The pooled samples were homogenized in a mixer mill and then subjected to further analyses. No significant difference was observed in moisture, protein, fat, ash, carbohydrate, or energy contents among any of the grafted lines (**Supplementary Table S18**). These results indicate that the use of a different type of rootstock (tobacco) and the expression of the *Luc* gene in tobacco rootstocks did not influence the basic properties as food components in tomato fruits obtained from the heterograft lines (Nt/MT and NtLuc/MT).

## 4. Discussion

In this study, we performed multi-omics analysis to clarify the effects of hetero-transgrafting on the quality of fruits harvested from non-GM tomato scions (NtLuc/MT plants using Luc expressing tobacco as rootstock). For comparison, we also analyzed the tomato fruits harvested from heterografted plants prepared using non-GM tobacco rootstock (Nt/MT). Irrespective of GM- or non-GM tobacco rootstocks, our multi-omics analysis demonstrated that several tobacco-derived molecules (proteins and metabolites) were present in the tomato fruits (Figs. 5,7B), indicating that substance exchanges across the graft junction occurred in the grafted plants composed of tomato scions and tobacco rootstocks. Proteome analysis suggested that elongation factor 1-alpha with a size of 49 kDa and lipoxygenase with a size of 97 kDa were tobacco-derived proteins (**Fig. 5**). No information is available for these two tobacco-derived proteins with regard to allergenicity or toxicity. Assuming that the full-length sequence of the protein was transferred from the tobacco rootstock to the tomato fruit, it is possible that proteins larger than 90 kDa in size could pass through the grafting junction. However, the fact that only two tobacco proteins were detected suggests that protein translocation might occur at very low frequency. In contrast, the transcript levels of the genes encoding tomato allergenic proteins Sola l 4.0101 (Solyc09g090990.2) and Sola l 4.0201 (Solyc09g090980.3) in the tomato fruits decreased in the heterograft line and the hetero-transgraft line (**Supplementary Tables S3 and S5**).

As described above, transcriptomic, proteomic, and metabolomic analyses revealed that heterografting using tobacco rootstocks (Nt/MT and NtLuc/MT) had slight effects on the profiles of metabolome and transcriptome of the tomato fruits. The expression levels of stress-related genes were reduced in the heterograft lines, although the reasons for this are not clear. First, the expression of environmental stress-related genes is likely to fluctuate in grafting; similar gene expression changes were in fact observed in tobacco homograft plants^21^^)^. In addition, the current results indicated that cellular metabolic functions are affected by grafting itself, regardless of heterografting or homografting (Fig. 7). It is possible that the successful establishment of grafting between different plant species may involve the suppression of defense response systems in both grafting partners. It should be noted that specific alterations due to the use of GM-tobacco rootstock (NtLuc) were not evident with respect to the quality of the tomato fruits (Figs. 2, 4,6). The *Luc* gene products (transcript and protein) were not detected in the non-GM tomato fruits under our experimental conditions. In the proteome analysis, significant differences in the accumulation levels of proteins related to allergies and biosynthesis of a toxic steroidal glycoalkaloid, α-tomatine, were not detectable in either heterograft or hetero-transgraft lines. Intriguingly, compared with the non-grafted tomato (MT), the α-tomatine levels (Fig. 7A) were significantly reduced in the grafted plants (MT/MT, Nt/MT, and NtLuc/MT). In this study, we did not include the fruits from non-grafted tomato in the proteome analysis, so the decreased α-tomatine contents in the grafted plants (Fig. 7A) cannot be discussed in relation to possible fluctuations of the metabolic activities involved in the α-tomatine accumulation.

The increase in the nicotine contents of the non-GM tomato fruits from heterograft line (Nt/MT) and hetero-transgraft line (NtLuc/MT) (Fig. 7B), which are attributable to the transport from the tobacco rootstocks, were confirmed to be far below the levels that raise toxicity issues. It is known that the use of tobacco as the rootstocks increased the nicotine levels in the leaves and fruits of the scions due to the transport from roots: the nicotine levels in the ripe fruits from the tomato scion of the grafted plants with tobacco rootstocks were 0.4 to 1.0 μg/g FW^33^^)^ and 21.4 to 72.0 μg/g FW^34^^)^. In this study, the nicotine contents reached 5.0 to 15.4 μg/g FW in the heterograft line and 5.0 to 13.6 μg/g FW in the hetero-transgraft line (Fig. 7B). Considering the acute reference dose (ARfD) and acceptable daily intake (ADI) (0.0008 mg/kg bw/day) for oral exposure to nicotine in food^35^^)^, the nicotine content of tomato fruits reached levels that cannot be recommended for daily consumption (Fig. 7B). In this study, it was assumed in advance that nicotine would be transported from the tobacco rootstock to the tomato scion. For the commercial use of transgrafting, it is critical to adopt rootstocks that do not produce toxic metabolites. Thus, the presence or absence of history of safe use (HOSU) of the candidate plant species for rootstocks is an important safety evaluation point. Food compositional analysis demonstrated that neither the heterografting nor hetero-transgrafting affected the properties of basic food components (moisture, protein, fat, ash, carbohydrate, and energy) in the non-GM tomato fruits (**Supplementary Table S18**).

We have previously reported that no marked food safety assessment concerns were detected in grafted crops, including GM tomato rootstocks expressing a recombinant β-glucuronidase protein, tobacco RdDM-inducing rootstock, and a GM potato scion expressing FT peptides^20^^,^^21^^,^^22^^)^. There have been many reports regarding the metabolic impacts on the scion edible parts of grafted crops^36^^,^^37^^,^^38^^)^ due to the reciprocal exchange of substances between scion and rootstock. Such substances include metabolites, plant hormones, peptides, mRNAs, and small RNAs^39^^)^. These results indicate that metabolites, including toxic secondary metabolites, may be transported from rootstocks and thus accumulate in the edible portions of scions. In other words, grafting itself has inherent food safety concerns. Most of the current reports refer only to grafting using non-GM plants, but there is no doubt that the use of GM plants for transgrafting will expand in the future. Rootstocks are selected to confer valuable traits such as biotic and/or abiotic stress resistance to commercial crop scions. In this study, we showed that toxic secondary metabolites (nicotine and its derivative) were in fact transported from rootstock to scion and that the grafting itself has significant impacts on the fruit quality of the scion parts. Similarly, proteins are transferred from the rootstock to the edible part of the scion. Importantly, the current study demonstrated that the effect of heterografting is greater than that of transgrafting with regard to the movement of rootstock proteins. A detailed safety evaluation should also be carried out for food production with the aid of heterografting using plants with no prior food experience. Food safety evaluation with regard to grafted plants can only be guaranteed on an individual basis, considering the intrinsic characteristics of the plants used for grafting and, if GM plants are used, what genetic modifications have been executed. Further investigations (i.e., the movement of gene products under biotic and abiotic stress conditions) are needed to clarify whether variable environmental conditions and growth periods may influence the qualities of the non-GM edible parts produced by such transgrafted plants.

## Supplementary materials

**Supplementary Figures:** 

**Supplementary Tables:** 
