# Supplementary material for: Multi-omics Analyses of Non-GM Tomato Scion Engrafted on GM Rootstocks
Source: Food Saf (Tokyo). 2023 Sep 6;11(3):41–53. doi: 10.14252/foodsafetyfscj.D-23-00005 (PMC10514396; doi:10.14252/foodsafetyfscj.D-23-00005)
Supplement: Supplementary file 1 [file foodsafetyfscj-11-41-s001.pdf]

Supplementary Figures

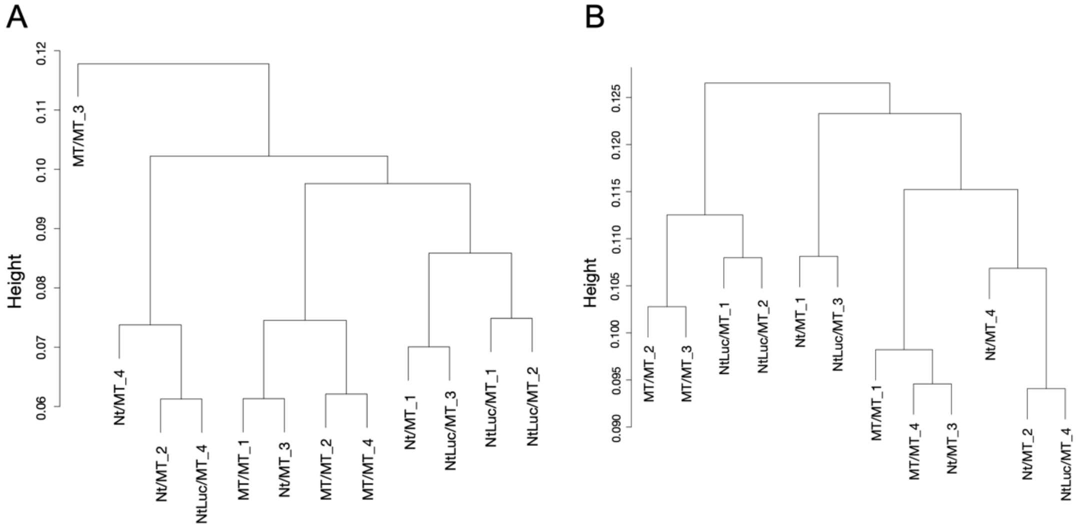

Supplementary Fig. S1 Hierarchical cluster analysis using Ward's method for TPM expression data. (A) Hierarchical cluster analysis using TPM data generated by alignment to tomato transcriptome data (ITAG4.0_cDNA.fasta). (B) Hierarchical cluster analysis using TPM data generated by alignment to tobacco transcriptome data (Nitab-v4.5_cDNA.Edwards2017.fasta).

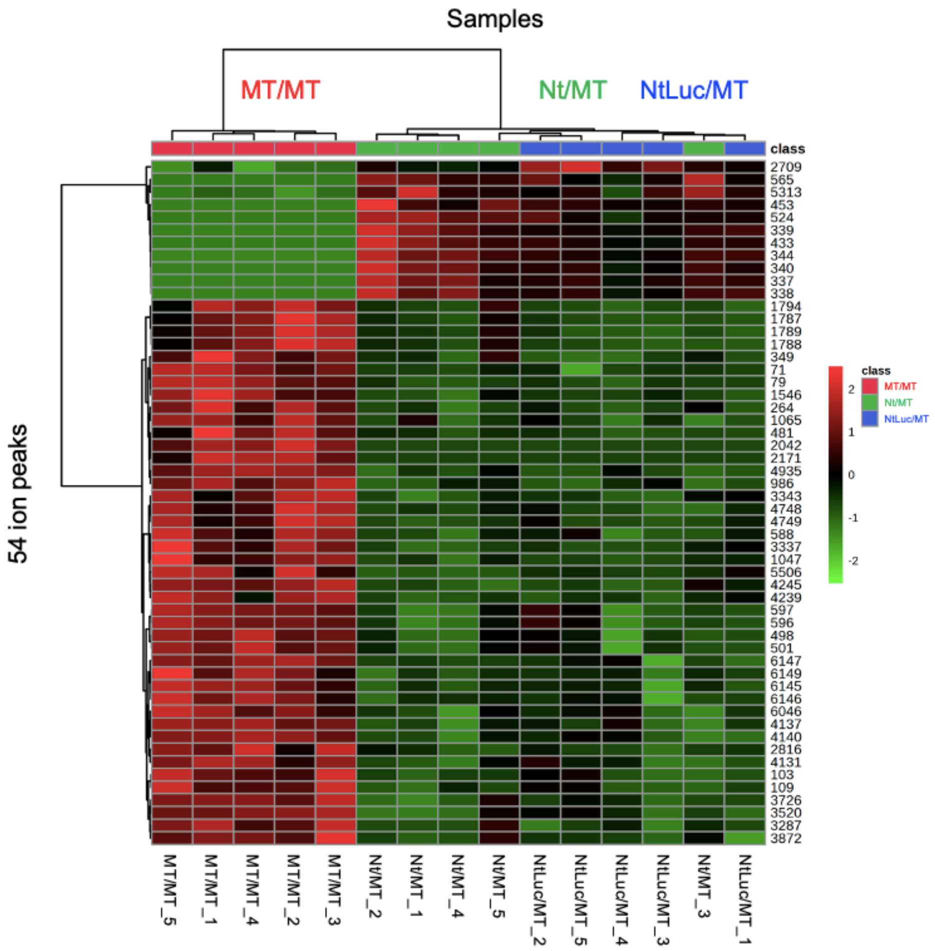

Supplementary Fig. S2 Hierarchal clustering analysis of metabolomic data of fruits from MT/MT (homograft), Nt/MT (heterograft), and NtLuc/MT (hetero-transgraft). Hierarchical clustering analysis using the auto-scaling ion peak intensity values of 54 statistically different ion peaks among MT/MT, Nt/MT, and NtLuc/MT (based on the result of one-way analysis of variance in Fig. 6B) was performed by Ward's method. The row displays the ion peak ID and the column displays the sample. Ion peaks with relatively high intensities are displayed in red, while ion peaks with relatively low intensities are displayed in green.

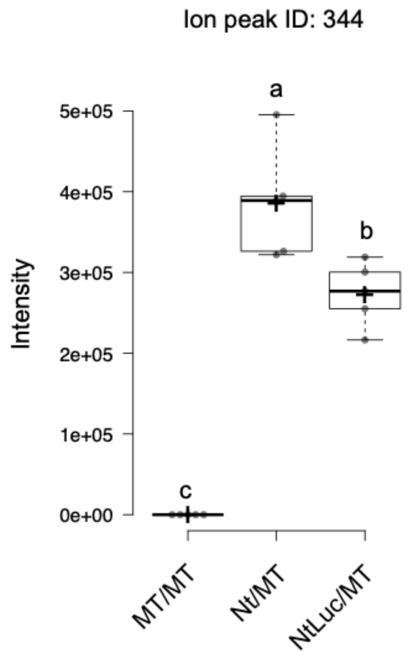

Supplementary Fig. S3 A comparison of ion peak intensities of ion peak ID 344 that showed statistically significant difference between Nt/MT (heterograft) and NtLuc/MT (hetero-transgraft). Tukey's honestly significant difference test was applied to the three groups. Different letters above the box plot indicate statistical differences at $p < 0.05$.
