# Supplementary material for: Multi-omics Analyses of Non-GM Tomato Scion Engrafted on GM Rootstocks
Source: Food Saf (Tokyo). 2023 Sep 6;11(3):41–53. doi: 10.14252/foodsafetyfscj.D-23-00005 (PMC10514396; doi:10.14252/foodsafetyfscj.D-23-00005)
Supplement: Supplementary file 2 [file foodsafetyfscj-11-41-s002.pdf]

Table S1. Alignment ratio for Tomato and Tobacco transcriptome data of each sample.

Sample	Reads	Alignment ratio for tomato transcript data (%)[a]	Alignment ratio for tobacco transcript data (%)[b]
MT/MT_1	11,450,282	81.35	22.98
MT/MT_2	10,667,612	83.09	22.65
MT/MT_3	9,944,285	82.46	22.07
MT/MT_4	12,244,267	81.77	21.62
Nt/MT_1	9,041,700	79.64	21.08
Nt/MT_2	9,906,425	82.30	23.64
Nt/MT_3	10,619,151	80.67	22.40
Nt/MT_4	8,295,937	80.78	21.56
NtLuc/MT_1	9,595,029	81.00	22.20
NtLuc/MT_2	9,578,594	80.48	21.28
NtLuc/MT_3	9,353,356	80.97	22.49
NtLuc/MT_4	10,090,871	82.58	23.20

[a]ITAG4.0_cDNA.fasta was used as tomato (*Solanum lycopersicum*) transcriptome data.
[b]Nitab_v4.5_Edwards2017_cDNA.fasta was used as tobacco (*Nicotiana tabacum*) transcriptome data.

Table S2. List of DEGs showing FDR < 0.05 in the comparison of Nt/MT and MT/MT.

**Gene**	**logFC: (Nt/MT) / (MT/MT)**	**logCPM**	**PValue**	**FDR**	**Description**
Solyc01g095140.4	3.441219592	7.97085634	5.15E-11	1.30E-06	Desiccation protectant protein Lea14-like protein (AHRD V3.3 *** A0A2G3BT76_CAPCH)
Solyc04g078880.3	2.481052799	4.22369048	1.73E-10	2.19E-06	cold regulated protein 27 (AHRD V3.3 *** AT5G42900.2)
Solyc03g019690.1	-5.599793064	1.87924673	8.05E-10	5.09E-06	Serine protease inhibitor 1 (AHRD V3.3 *** A0A2G2XWH1_CAPAN)
Solyc12g044950.3	-7.424526392	0.61422955	6.97E-10	5.09E-06	lipid desaturase
Solyc05g007520.1	5.725992806	0.41828363	3.30E-09	1.39E-05	Ubiquitin carboxyl-terminal hydrolase 46 (AHRD V3.3 *-* A0A2G3AUZ3_CAPCH)
Solyc01g108100.3	2.117889069	5.01678978	3.23E-09	1.39E-05	cold regulated protein 27 (AHRD V3.3 *** AT5G42900.2)
Solyc04g071070.2	-2.781125904	4.1384308	4.53E-09	1.64E-05	Unknown protein
Solyc10g055810.2	-3.156435203	5.41604415	5.61E-09	1.77E-05	chitinase Z15140
Solyc09g097760.3	-3.804166477	3.04321135	6.47E-09	1.82E-05	Glycine-rich protein (AHRD V3.3 *-* A0A2G2W2G9_CAPBA)
Solyc04g064690.4	6.887055598	3.79803212	1.05E-08	2.42E-05	Peroxidase (AHRD V3.3 *** K4BT60_SOLLC)
Solyc09g007010.1	-4.188090266	6.00343431	1.05E-08	2.42E-05	Pathogenesis-related protein 1 (AHRD V3.3 *** Q75QH2_CAPCH)
Solyc06g073080.4	3.497150983	2.21578507	1.58E-08	2.84E-05	2-oxoglutarate (2OG) and Fe(II)-dependent oxygenase superfamily protein (AHRD V3.3 *** A0A2U1QGX7_ARTAN)
Solyc02g085910.4	2.775998488	5.88654189	1.58E-08	2.84E-05	LOB domain-containing protein (AHRD V3.3 *** A0A2U1NKN8_ARTAN)
Solyc02g014860.3	2.080596185	6.25586557	1.71E-08	2.84E-05	Chaperone protein DnaJ (AHRD V3.3 *** A0A1J3ID64_NOCCA)
Solyc01g102610.3	-2.549735014	6.35151724	1.66E-08	2.84E-05	Ferric reduction oxidase 6 (AHRD V3.3 *** A0A2G3BUN6_CAPCH)
Solyc08g080650.3	-2.85834168	5.78819991	1.80E-08	2.84E-05	PATHOGENESIS RELATED PROTEIN P23
Solyc04g081960.1	2.49778639	3.38122334	2.47E-08	3.67E-05	Syringolide-induced protein 14-1-1 (AHRD V3.3 *** A0A2K3M7L2_TRIPR)
Solyc02g081030.4	2.466362894	7.51420021	3.43E-08	4.27E-05	ERD (Early-responsive to dehydration stress) family protein (AHRD V3.3 *-* A0A2U1NFB6_ARTAN)
Solyc04g082200.2	2.167775147	10.9619072	3.12E-08	4.27E-05	dehydrin
Solyc10g006860.4	-1.710202066	5.92979411	3.55E-08	4.27E-05	NAD(P)-binding Rossmann-fold superfamily protein (AHRD V3.3 *** F4IPI3_ARATH)
Solyc09g006005.1	-4.041918565	3.94546126	3.47E-08	4.27E-05	Pathogenesis-related protein 1 (AHRD V3.3 *** Q75QH2_CAPCH)
Solyc11g012360.2	-2.259971442	3.28124765	4.31E-08	4.95E-05	Tonoplast dicarboxylate transporter (AHRD V3.3 *** A0A2G3AZI6_CAPCH)
Solyc03g025670.3	-3.166825844	1.81431699	7.08E-08	7.78E-05	PAR1 (AHRD V3.3 *** A0A2U1P9T7_ARTAN)
Solyc10g017970.1	-2.527732953	6.20008274	1.32E-07	0.00013607	Chitinase (AHRD V3.3 *-* B8QVJ5_ZEAMP)
Solyc12g100270.2	-4.558475779	2.39885204	1.35E-07	0.00013607	Fatty acid hydroxylase superfamily (AHRD V3.3 *** A0A2U1L8N4_ARTAN)
Solyc05g053080.2	2.999280683	6.34118765	1.72E-07	0.00015003	Unknown protein
Solyc07g064410.1	-2.058262444	4.19998802	1.71E-07	0.00015003	Fatty acid desaturase 4, chloroplastic (AHRD V3.3 *** A0A1U8FLY9_CAPAN)
Solyc06g082240.2	-4.158777591	1.69267417	1.65E-07	0.00015003	Laccase (AHRD V3.3 *** K4CA83_SOLLC)
Solyc01g106920.4	-4.822278763	0.48293899	1.57E-07	0.00015003	Nucleobase-ascorbate transporter 8 (AHRD V3.3 *** A0A1U8DZ27_CAPAN)
Solyc09g009530.4	1.60557658	3.91017283	2.44E-07	0.00019905	Alpha/beta-Hydrolases superfamily protein (AHRD V3.3 *** Q9SJM9_ARATH)
Solyc04g074840.3	-2.721138989	7.77367001	2.43E-07	0.00019905	Protein DETOXIFICATION (AHRD V3.3 *** A0A218X5P1_PUNGR)
Solyc10g017980.1	-2.694514337	5.83896705	2.90E-07	0.00022932	Chitinase (AHRD V3.3 *-* B8QVJ5_ZEAMP)
Solyc10g055800.2	-2.632811945	5.08229902	3.56E-07	0.0002731	Chitinase (AHRD V3.3 *** B9VRK7_CAPAN)

Solyc06g076350.3	2.368642619	4.03756843	3.86E-07	0.00027851	LePCL1
Solyc06g074820.3	-1.977132983	3.28420111	3.81E-07	0.00027851	tonoplast intrinsic protein 1.1
Solyc12g087950.1	2.219807102	4.39202751	4.27E-07	0.00030013	AT-hook motif nuclear-localized protein (AHRD V3.3 *** A0A1U8GD78_CAPAN)
Solyc03g080190.3	2.619460989	3.48712947	4.83E-07	0.00033004	2-oxoglutarate (2OG) and Fe(II)-dependent oxygenase superfamily protein (AHRD V3.3 *** A0A2U1QGX7_ARTAN)
Solyc09g010210.3	-1.926784603	8.75961602	5.29E-07	0.00035217	endo-1,4-beta-glucanase precursor (Cel2)
Solyc06g051400.3	-1.583233345	7.14101966	5.66E-07	0.00036711	omega-3 fatty acid desaturase
Solyc02g085400.3	2.638029447	3.64952766	7.26E-07	0.00045918	Sulfite exporter TauE/SafE family protein (AHRD V3.3 *** A0A2U1KY14_ARTAN)
Solyc06g068990.4	3.9277967	1.06146468	8.31E-07	0.0004992	Mitogen-activated protein kinase (AHRD V3.3 *** A0A2G2WLN1_CAPBA)
Solyc12g005910.2	1.620942909	5.74505496	8.69E-07	0.0004992	B-cell receptor-associated 31-like (AHRD V3.3 *** A0A200QH03_9MAGN)
Solyc04g071900.4	-2.644206117	2.78004968	8.36E-07	0.0004992	Peroxidase (AHRD V3.3 *** K4BTH7_SOLLC)
Solyc10g079860.2	-4.207516429	2.90479745	8.54E-07	0.0004992	LEQB L.esculentum TomQ'b beta(1,3)glucanase
Solyc10g081170.2	1.681759994	8.94077695	9.56E-07	0.00052694	Calmodulin 2
Solyc05g024010.3	1.449029099	4.75220494	9.59E-07	0.00052694	Zinc finger protein CONSTANS-LIKE 15 (AHRD V3.3 *** A0A2G2VMU9_CAPBA)
Solyc12g100250.3	-4.785290122	0.90843006	1.14E-06	0.00061061	Fatty acid desaturase (AHRD V3.3 *** A0A200QRI1_9MAGN)
Solyc01g100000.3	2.320711851	3.02993239	1.33E-06	0.00070028	F-box domain, Phloem protein 2-like protein (AHRD V3.3 *** A0A2U1MNC1_ARTAN)
Solyc02g090210.3	-4.535708544	0.25893477	1.46E-06	0.00075469	GDSL esterase/lipase (AHRD V3.3 *** A0A2G3D8U2_CAPCH)
Solyc08g066705.1	-1.638878277	5.73655871	1.54E-06	0.00077951	Gag-pol polyprotein (AHRD V3.3 *** E6Y5Q4_SOLLC)
Solyc04g083140.2	-4.682749043	0.77159149	1.67E-06	0.00082557	Cytochrome (AHRD V3.3 *** A0A1U8F0H8_CAPAN)
Solyc05g053070.4	2.954364549	7.52974948	1.81E-06	0.00086546	Unknown protein
Solyc04g015750.3	-1.798180501	8.22107226	1.79E-06	0.00086546	CobN/magnesium chelatase (AHRD V3.3 *** A0A200PZ28_9MAGN)
Solyc01g088090.3	-1.562549128	8.17899916	2.07E-06	0.00096958	Pheophytinase, chloroplastic (AHRD V3.3 *** A0A2G3C5T3_CAPCH)
Solyc07g007750.3	-1.80233388	4.89476561	2.12E-06	0.00097494	Defensin protein (AHRD V3.3 *** B1N678_SOLLC),Pfam:PF00304
Solyc04g074020.2	-4.097794052	0.31929818	2.32E-06	0.00104626	Receptor protein kinase, putative (AHRD V3.3 *** A0A061FG24_THECC)
Solyc07g062700.3	1.691346213	9.50962619	2.41E-06	0.00106788	Calcium-binding EF-hand family protein (AHRD V3.3 *** A0A2U1MZJ3_ARTAN)
Solyc06g005680.4	3.537268573	1.61182244	2.69E-06	0.00117335	Two-component response regulator (AHRD V3.3 *-* C0HE02_MAIZE)
Solyc08g083250.4	-1.507979335	5.95157686	2.98E-06	0.00127608	Purple acid phosphatase (AHRD V3.3 *** A0A2G3AD35_CAPAN)
Solyc07g009510.1	-5.552230207	1.90623023	3.89E-06	0.00163776	Chitinase 12 (AHRD V3.3 *** A0A2G2VG15_CAPBA)
Solyc07g063320.3	2.267413186	3.50079072	4.54E-06	0.00185161	LanC-like protein GCR2 (AHRD V3.3 *** A0A2G3C294_CAPCH)
Solyc08g075370.3	1.709219814	7.94762568	4.49E-06	0.00185161	Unknown protein
Solyc11g005240.1	3.62491992	-0.2077815	5.45E-06	0.00214576	protein SENSITIVITY TO RED LIGHT REDUCED 1-like (AHRD V3.3 *-* A0A2I4GZX1_9ROSI)
Solyc07g009530.1	-2.948316105	2.1277518	5.52E-06	0.00214576	Chitinase (AHRD V3.3 *-* B8QVH4_ZEAMP)
Solyc12g049030.1	-4.576052129	1.73723005	5.49E-06	0.00214576	Fatty acid desaturase (AHRD V3.3 *** A0A200QRI1_9MAGN)
Solyc05g050010.3	-1.602227595	6.86799145	5.76E-06	0.00220481	1-aminocyclopropane-1-carboxylic acid synthase-4
Solyc04g081650.3	2.194973687	2.19784526	6.34E-06	0.00239394	Cyclin (AHRD V3.3 *-* A0A200PQW1_9MAGN)
Solyc01g060020.4	-8.959775487	1.97707953	8.27E-06	0.00307545	beta-1,3-glucanase TOMB13GLUB
Solyc10g076710.3	-2.209255417	1.5161055	9.28E-06	0.00339947	Phosphoinositide phospholipase C (AHRD V3.3 *** O49950_SOLTU)

Solyc07g064160.3	1.820074423	7.47468071	1.06E-05	0.00351808	Thiamine thiazole synthase, chloroplastic (AHRD V3.3 *** A0A2G2WFN7_CAPBA),Pfam:PF01946
Solyc10g081570.3	1.668629748	9.08076149	1.05E-05	0.00351808	Marmande
Solyc05g051900.3	1.661449269	2.84790325	1.05E-05	0.00351808	Major facilitator superfamily (AHRD V3.3 *** A0A200QUR2_9MAGN)
Solyc09g097960.3	1.630665904	4.32940061	1.02E-05	0.00351808	NAD(P)-linked oxidoreductase, aldo/keto reductase family protein (AHRD V3.3 *** A0A1Y1HY98_KLENI)
Solyc01g006680.4	1.537600532	4.72912268	1.00E-05	0.00351808	2-oxoglutarate (2OG) and Fe(II)-dependent oxygenase superfamily protein (AHRD V3.3 *** A0A2U1NBL4_ARTAN)
Solyc08g079870.3	-2.545822403	1.26645225	1.05E-05	0.00351808	subtilisin
Solyc03g020030.3	-5.288879439	0.24415019	1.06E-05	0.00351808	Proteinase inhibitor type-2 (AHRD V3.3 *** A0A2G3A0T9_CAPAN)
Solyc11g069330.1	-1.108283192	5.49123281	1.11E-05	0.00364257	Peptide upstream protein (AHRD V3.3 *** Q9LFH7_ARATH)
Solyc05g052280.3	-2.997841481	0.55329509	1.23E-05	0.00399044	Peroxidase (AHRD V3.3 *** K4C1Q9_SOLLC)
Solyc02g068430.4	1.175573406	7.2705917	1.52E-05	0.00472648	Choline-phosphate cytidylyltransferase (AHRD V3.3 *** A0A2I0V8H3_9ASPA)
Solyc03g007370.3	-1.490917652	5.65849783	1.53E-05	0.00472648	Sigma factor (AHRD V3.3 *** A0A0G2STU5_9ROSI)
Solyc10g075150.2	-2.425110336	4.91504839	1.49E-05	0.00472648	Non-specific lipid-transfer protein (AHRD V3.3 *** A0A2G2YKI8_CAPAN)
Solyc07g005100.4	-2.549765974	2.02812779	1.53E-05	0.00472648	Chitinase/lysozyme (AHRD V3.3 *** Q43591_TOBAC)
Solyc06g084770.2	-1.253110323	6.02594618	1.56E-05	0.0047472	cytochrome P450 CYP72A219 (AHRD V3.3 --* XP_004244272.1)
Solyc03g006700.3	-3.094262897	0.32497192	1.62E-05	0.0048634	Peroxidase (AHRD V3.3 *** K4BE93_SOLLC)
Solyc01g079610.3	1.519121103	4.65398038	1.67E-05	0.00495843	DnaJ protein ERDJ3B (AHRD V3.3 *** A0A2G2Z6I0_CAPAN)
Solyc11g011210.2	-1.283491579	6.55888036	1.83E-05	0.00537732	Gibberellin-regulated protein 12 (AHRD V3.3 *** A0A1U8GMV5_CAPAN)
Solyc06g068230.4	-1.492897069	5.66706626	1.86E-05	0.00541373	Tetratricopeptide repeat (TPR)-like superfamily protein (AHRD V3.3 *** A0A2U1MYN5_ARTAN)
Solyc04g054190.3	-1.752238256	7.13044098	1.91E-05	0.0054816	Protein kinase superfamily protein (AHRD V3.3 *** A0A2U1MTH5_ARTAN)
Solyc09g091580.3	-1.516544719	7.23670588	1.98E-05	0.00560949	Protein kinase domain (AHRD V3.3 *** A0A200QUN4_9MAGN)
Solyc05g053610.2	-4.904323904	1.98898684	2.00E-05	0.00560949	Pleiotropic drug resistance protein (AHRD V3.3 *** C8CA13_CUCSA)
Solyc04g072033.1	-1.332539866	5.67584158	2.09E-05	0.00581219	NAD(P)-binding Rossmann-fold superfamily protein (AHRD V3.3 *** Q9SZ91_ARATH)
Solyc05g011890.1	-1.66698348	4.09696267	2.23E-05	0.00614211	Sulfotransferase (AHRD V3.3 *** A0A2G3B6Y2_CAPCH)
Solyc10g085240.1	-2.503104968	3.4757047	2.27E-05	0.00617947	UDP-glycosyltransferase 76E1 (AHRD V3.3 *** U76E1_SOLLC)
Solyc06g071500.3	-1.383656331	5.44981976	2.30E-05	0.00618097	Boron transporter 1 (AHRD V3.3 *** A0A2G2WM36_CAPBA)
Solyc11g072480.2	2.548834916	2.69858886	2.36E-05	0.00628131	Tetraspanin-3 (AHRD V3.3 *** A0A2G3BA66_CAPCH)
Solyc03g115770.3	1.940465901	5.63561653	2.42E-05	0.00629773	Two-component response regulator-like APRR5 (AHRD V3.3 *** A0A2G3CZB4_CAPCH)
Solyc10g055820.3	-3.949631627	1.64897171	2.40E-05	0.00629773	Chitinase (AHRD V3.3 *** B9VRK7_CAPAN)
Solyc06g008920.3	-1.388416361	6.42366276	2.47E-05	0.00636428	AMP-dependent synthetase/ligase (AHRD V3.3 *** A0A200QNF3_9MAGN)
Solyc04g076550.3	-1.074479147	4.61805912	2.54E-05	0.00649644	HYPERSENSITIVE TO PI STARVATION 4 (AHRD V3.3 *-* AT1G58250.1)
Solyc06g050440.3	-3.259069895	1.98836136	2.82E-05	0.00712632	Peroxidase (AHRD V3.3 *** K4C5I8_SOLLC)
Solyc06g036110.1	1.222696457	5.53950832	2.91E-05	0.00727442	Calcium-dependent lipid-binding (CaLB domain) family protein (AHRD V3.3 *** A0A2U1LCI8_ARTAN)
Solyc03g112170.1	2.82813108	0.24024905	3.01E-05	0.00735935	pectinesterase inhibitor-like (AHRD V3.3 *** A0A2G3A1B6_CAPAN)
Solyc09g082860.3	1.36794419	5.43884623	3.03E-05	0.00735935	ATP sulfurylase (AHRD V3.3 *** Q8SAG1_SOYBN)

Solyc02g089350.3	-1.52562391	3.84672841	3.06E-05	0.00735935	Gibberellin regulated protein (AHRD V3.3 *** A0A2U1PE48_ARTAN)
Solyc09g090990.2	-3.62941108	1.60222629	3.03E-05	0.00735935	Major allergen Pru ar 1 (AHRD V3.3 *** Q5GMN2_CAPCH)
Solyc01g100370.3	1.783780065	8.73630898	3.19E-05	0.00760918	Adenine nucleotide alpha hydrolases-like superfamily protein (AHRD V3.3 *** A0A2U1PDN0_ARTAN)
Solyc04g081240.2	1.381005697	5.43605559	3.28E-05	0.00773919	Auxin Response Factor 5
Solyc10g076660.2	2.805824299	1.44260479	3.31E-05	0.00774268	2-oxoglutarate (2OG) and Fe(II)-dependent oxygenase superfamily protein (AHRD V3.3 *** F4J670_ARATH)
Solyc08g016080.3	-1.375580669	6.69482683	3.64E-05	0.00843492	High chlorophyll fluorescence phenotype 173 (AHRD V3.3 *** Q8W4D6_ARATH)
Solyc08g078870.3	2.658819194	3.6888428	3.70E-05	0.00847547	Bifunctional inhibitor/lipid-transfer protein/seed storage 2S albumin superfamily protein (AHRD V3.3 *** A0A2U1PU47_ARTAN)
Solyc06g063060.3	1.314496537	3.67122034	3.72E-05	0.00847547	Dormancy/auxin associated protein (AHRD V3.3 *-* A0A2K3JP12_TRIPR)
Solyc09g092580.4	-3.378238851	0.30161399	3.83E-05	0.00864689	Cytochrome (AHRD V3.3 *** A0A2G2ZTD7_CAPAN)
Solyc02g077110.3	2.111387038	3.40177092	3.94E-05	0.00876248	Phospholipase A1-II 1 (AHRD V3.3 *** A0A2G2XE87_CAPBA)
Solyc04g079940.3	1.310592037	4.58526374	3.95E-05	0.00876248	NAC domain-containing protein (AHRD V3.3 *** A0A2U1N781_ARTAN)
Solyc12g006240.2	1.853288884	3.03236554	3.99E-05	0.00878171	Zinc finger protein CONSTANS-LIKE 9 (AHRD V3.3 *** A0A2G2YTW9_CAPAN)
Solyc07g061990.3	-1.636342603	7.41931679	4.07E-05	0.00886966	Solanesyl diphosphate synthase (AHRD V3.3 *** Q1W5D1_HEVBR)
Solyc06g068960.1	2.882212711	0.49129429	4.17E-05	0.00894128	Calcium-binding allergen Ole e 8 (AHRD V3.3 *** A0A2G2WLM3_CAPBA)
Solyc12g088460.3	-1.467698569	6.56597513	4.17E-05	0.00894128	Cytochrome P450 (AHRD V3.3 *** A0A200Q0W8_9MAGN)
Solyc09g008830.3	2.754257974	5.72844174	4.44E-05	0.00944333	Sequence-specific DNA binding transcription factor (AHRD V3.3 *** A0A2U1LFW8_ARTAN)
Solyc03g078090.4	2.989891399	0.0194565	4.74E-05	0.00999092	Pectinesterase (AHRD V3.3 *** A0A2I4GT13_9ROSI)
Solyc12g044230.2	2.179746491	5.28144878	4.82E-05	0.01007267	Sulfite exporter TauE/SafE family protein (AHRD V3.3 *** A0A2U1KY14_ARTAN)
Solyc11g006300.2	-2.714900943	4.8018016	4.90E-05	0.01014879	3-oxo-5-alpha-steroid 4-dehydrogenase (AHRD V3.3 *** A0A200PYH0_9MAGN)
Solyc01g088560.3	1.10719568	7.03661837	4.99E-05	0.01017374	Rab family GTPase (AHRD V3.3 *** A0A1Y1IFP9_KLENI)
Solyc01g006290.4	-1.551887691	4.40230736	4.99E-05	0.01017374	Peroxidase (AHRD V3.3 *** K4ASJ5_SOLLC)
Solyc07g064720.3	2.01167152	3.22693705	5.48E-05	0.01108032	Gdsl esterase/lipase (AHRD V3.3 *** A0A2P4JQK3_QUESU)
Solyc01g106790.3	-1.647120325	2.95701591	5.52E-05	0.01108032	Tubulin alpha-6 chain, putative (AHRD V3.3 *** Q8LED1_ARATH)
Solyc02g079490.3	-1.687400389	7.84591754	5.64E-05	0.01123509	HXXXD-type acyl-transferase family protein (AHRD V3.3 *** Q9MAP9_ARATH)
Solyc10g085420.3	1.640990023	4.92923388	5.77E-05	0.01140387	Protein LURP-one-related 15 (AHRD V3.3 *** A0A2G2VU46_CAPBA)
Solyc01g059965.1	-3.512892938	5.27029223	6.00E-05	0.0117565	Glucan endo-1,3-beta-glucosidase B (AHRD V3.3 *** E13B_SOLLC)
Solyc03g116590.3	0.970360425	7.65145301	6.37E-05	0.01238647	Embryo-specific protein (AHRD V3.3 *** A0A2K3LFD9_TRIPR)
Solyc12g098920.2	-1.491202333	4.9102532	6.52E-05	0.01258049	Guanosine-3'5'-bis(Diphosphate) 3'-pyrophosphohydrolase (AHRD V3.3 *** E5GC47_CUCME)
Solyc12g008940.2	1.094868857	7.56998018	6.62E-05	0.01267762	Nucleosome assembly protein family (AHRD V3.3 *** A9TVZ4_PHYPA)
Solyc02g063250.3	1.714270614	3.2616093	7.04E-05	0.01331987	Peptide methionine sulfoxide reductase MsrA (AHRD V3.3 *** A0A1D8JN47_9ENTR)
Solyc04g072020.3	1.48968728	4.75818274	7.06E-05	0.01331987	Choline/Ethanolamine kinase (AHRD V3.3 *** A0A200QCA0_9MAGN),Pfam:PF01633
Solyc01g080460.3	-1.326069767	10.8482145	7.18E-05	0.0134562	Pyruvate, phosphate dikinase (AHRD V3.3 *** A0A0M8KRU1_NICAT)
Solyc12g042793.1	3.653581711	0.47685088	7.31E-05	0.01349203	coiled-coil domain-containing protein 18 (AHRD V3.3 *-* XP_010325663.1)
Solyc09g082690.3	-1.945474414	10.1651046	7.27E-05	0.01349203	superoxide dismutase
Solyc10g086180.2	-1.363457835	5.82870384	7.43E-05	0.01362075	Phenylalanine ammonia-lyase (AHRD V3.3 *** A0A2G2VU16_CAPBA)

Solyc01g095970.3	1.282575735	5.66234221	7.58E-05	0.01369128	Dynamin-like protein (AHRD V3.3 *** F4K015_ARATH)
Solyc05g052950.4	-2.533385666	5.2910646	7.58E-05	0.01369128	Regulator of chromosome condensation (RCC1) family protein (AHRD V3.3 *** Q9M2S1_ARATH)
Solyc05g009340.1	4.593174142	1.24975583	7.85E-05	0.01398376	Unknown protein
Solyc01g005300.4	1.080283626	7.05685576	7.83E-05	0.01398376	Adagio-like protein 1 (AHRD V3.3 *** A0A1U8H9V5_CAPAN)
Solyc12g099980.3	-2.812255782	0.11108479	8.07E-05	0.01426092	receptor-like protein 12 (AHRD V3.3 *** A0A1U8F785_CAPAN)
Solyc09g008175.1	1.549590239	4.42182352	8.34E-05	0.01463707	SAUR-like auxin-responsive protein family (AHRD V3.3 *** A0A2U1NT85_ARTAN)
Solyc04g150103.1	-1.550103702	2.26426955	8.58E-05	0.01496513	Sn-1 protein (AHRD V3.3 *-* Q42393_CAPAN)
Solyc05g007950.4	2.773924032	0.41633792	8.76E-05	0.01516482	LERNALE L.esculentum ribonuclease le
Solyc03g020060.3	-4.129927638	-0.5814659	8.86E-05	0.01523811	Proteinase inhibitor type-2 (AHRD V3.3 *** A0A2G2V3L6_CAPBA)
Solyc05g056477.1	-2.117809444	3.41028587	9.06E-05	0.01536855	NAC domain-containing protein (AHRD V3.3 *** A0A2K3LLY1_TRIPR)
Solyc04g040130.1	-4.4960874	1.24943827	9.01E-05	0.01536855	Fatty acid desaturase (AHRD V3.3 *** E7CCD0_CAMMC)
Solyc09g083390.3	-1.368029857	4.23095784	9.19E-05	0.01548902	zinc knuckle (CCHC-type) family protein (AHRD V3.3 *-* AT5G43630.4)
Solyc01g111520.3	1.085897374	7.12355461	9.30E-05	0.01557519	Calcium-dependent lipid-binding (CaLB domain) family protein (AHRD V3.3 *** A0A1P8BD17_ARATH)
Solyc09g075300.3	1.587587071	2.96518773	9.65E-05	0.01603013	Epoxide hydrolase 2 (AHRD V3.3 *** B6T857_MAIZE)
Solyc08g075210.2	-1.174354452	5.97327261	9.83E-05	0.01603013	HXXXD-type acyl-transferase family protein (AHRD V3.3 *** F4JBC7_ARATH)
Solyc03g115980.1	-1.244017648	4.67577685	9.72E-05	0.01603013	Geranylgeranyl diphosphate reductase, chloroplastic (AHRD V3.3 *** A0A2G2WR46_CAPBA)
Solyc01g097280.2	-3.257650567	-0.0075954	9.79E-05	0.01603013	Pathogenesis-related protein 4b (AHRD V3.3 *** A0A0H3TYE5_CAPAN)
Solyc01g105350.3	-2.040868835	2.11827506	9.96E-05	0.01613806	Glycosyltransferase (AHRD V3.3 *** A0A1Q3B719_CEPFO)
Solyc07g053030.4	2.712041002	0.11663175	0.00010078	0.01623102	Auxin-responsive GH3 family protein (AHRD V3.3 *-* A0A2U1LNK3_ARTAN)
Solyc07g009230.3	-3.230690861	1.9848814	0.00010517	0.01682982	Defensin-like protein 1 (AHRD V3.3 *** A0A2G2ZS54_CAPAN)
Solyc12g042910.2	-1.608326179	2.93112963	0.0001127	0.01792247	DNA photolyase (AHRD V3.3 *** A0A200PQM0_9MAGN)
Solyc07g061950.4	1.186222402	5.67759023	0.00011508	0.01818545	C2 domain-containing protein (AHRD V3.3 *** A0A2G2WER0_CAPBA)
Solyc02g081550.3	-1.67322803	6.16655885	0.00011734	0.01842858	LeftsH6FtsH protease
Solyc10g007110.3	-1.146802925	8.26676788	0.00012497	0.01950548	Tyrosine aminotransferase (AHRD V3.3 *** A0A1U8EFD5_CAPAN)
Solyc04g054258.1	-2.861205893	-0.0438577	0.00013035	0.02021962	Cytochrome p450 (AHRD V3.3 *** A0A2K3NDQ9_TRIPR)
Solyc08g074682.1	-4.840990211	1.51993039	0.00013247	0.02042423	Polyphenol oxidase (AHRD V3.3 *** Q41427_SOLTU)
Solyc08g014000.3	-1.065361414	11.3186299	0.00013668	0.02094441	lipoxygenase A
Solyc06g066800.2	1.65355469	5.35041272	0.00013837	0.02107671	Nucleotide-diphospho-sugar transferases superfamily protein (AHRD V3.3 *** AT1G64980.3)
Solyc05g050007.1	-1.336039293	4.67360118	0.00014405	0.0218097	Serine/threonine-protein phosphatase 7 long form-like protein (AHRD V3.3 *-* A0A1J3IZ43_NOCCA)
Solyc05g007880.4	-1.764137654	2.39788457	0.00014798	0.02227168	cyclic dof factor 2-like (AHRD V3.3 *** A0A2I4E3P6_9ROSI)
Solyc01g095530.2	-1.938584114	2.83484479	0.00014942	0.02235573	hypothetical protein (AHRD V3.3 *-* AT5G41761.1)
Solyc00g500064.1	-0.903811837	5.39628837	0.00015122	0.02249187	Ycf15 (AHRD V3.3 *-* A0A346JLD3_9ASTE)
Solyc02g071860.4	-1.225999184	5.65751622	0.00015813	0.02338144	LRR_1 domain-containing protein/Pkinase_Tyr domain-containing protein/Malectin domain-containing protein (AHRD V3.3 *** A0A1Q3CJY9_CEPFO)
Solyc01g007100.3	-1.485338332	4.1960235	0.00016136	0.02372087	Digalactosyldiacylglycerol synthase 1, chloroplastic (AHRD V3.3 *** A0A1U8EIN3_CAPAN)

Solyc03g031550.1	6.029307955	-0.5146801	0.00016582	0.02423567	Cysteine-rich receptor-like protein kinase 25 (AHRD V3.3 *-* A0A2G2WWP2_CAPBA)
Solyc11g011170.2	-1.175754515	6.32681134	0.00016836	0.02446477	Senescence-associated family protein (AHRD V3.3 *** A0A2U1QIG8_ARTAN)
Solyc04g071150.3	-1.368082868	8.38009449	0.00017536	0.02533637	Cytochrome P450 (AHRD V3.3 *** A0A2U1NTV8_ARTAN)
Solyc05g051750.3	-1.893859413	1.96138643	0.00017897	0.02571117	TOMPRORNA prosystemin
Solyc07g008240.3	2.304918064	3.86394742	0.00018048	0.02578167	Non-symbiotic hemoglobin like (AHRD V3.3 *** A0A2R6R1Q2_ACTCH)
Solyc06g076140.4	-0.959219056	9.18644973	0.00018158	0.02579319	Metallothionein-like protein (AHRD V3.3 *-* A8DUB1_SOLLC)
Solyc02g089520.2	1.47204129	4.95221092	0.00018472	0.02609354	Zinc finger protein CONSTANS-LIKE 2 (AHRD V3.3 *** A0A2G3ABV8_CAPAN)
Solyc07g056708.1	2.342630065	0.87501604	0.00018749	0.02633693	DUF239 domain-containing protein/DUF4409 domain-containing protein (AHRD V3.3 *** A0A1Q3CSL7_CEPFO)
Solyc07g042500.4	-0.997057654	7.28195142	0.00019232	0.02686654	alpha/beta-Hydrolases superfamily protein (AHRD V3.3 *** AT2G03140.9)
Solyc12g096310.2	-1.196522813	6.03344849	0.0001961	0.02724443	plant/protein (Protein of unknown function%2C DUF599) (AHRD V3.3 *** AT5G10580.4)
Solyc12g056675.1	-3.420612213	4.70740895	0.00019984	0.0276118	Alpha/beta-hydrolases superfamily protein (AHRD V3.3 *** A0A2U1LX98_ARTAN)
Solyc05g013750.3	1.382111383	2.68734804	0.00020303	0.02789952	Protein-tyrosine-phosphatase MKP1 (AHRD V3.3 *** A0A2G3B907_CAPCH)
Solyc01g103650.3	-2.439546689	1.49021154	0.00020451	0.0279516	Alpha/beta-Hydrolases superfamily protein (AHRD V3.3 *** Q9XID7_ARATH)
Solyc07g043230.3	1.971143095	2.70778881	0.00020939	0.02846416	Zinc transporter protein (AHRD V3.3 *** A5BDR3_VITVI)
Solyc07g044980.3	1.288812948	5.24014717	0.00021304	0.02880555	NIM1-like protein 2
Solyc03g111930.4	2.069939187	2.4851247	0.0002184	0.02937302	Cytochrome P450 71A6 (AHRD V3.3 *** A0A2G2ZZC6_CAPAN)
Solyc11g068940.1	3.031906149	0.60725422	0.00022138	0.02961666	RING-type E3 ubiquitin transferase (AHRD V3.3 *** A0A2G2VPS7_CAPBA)
Solyc12g044954.1	-4.234844287	1.20680913	0.00022356	0.02975135	Cytochrome (AHRD V3.3 *** A0A2G2Y725_CAPAN)
Solyc04g051360.3	2.772486412	1.8799517	0.0002254	0.02983943	Ethylene Response Factor D.1
Solyc01g094370.3	0.992881752	6.45472896	0.00022817	0.03004835	remorin 2
Solyc02g081980.3	2.304794034	0.55347278	0.00023883	0.03128983	Apyrase (AHRD V3.3 *** A0A2G2XFE6_CAPBA)
Solyc09g065440.4	-3.235963681	-0.288762	0.00024074	0.03137748	Bifunctional inhibitor/lipid-transfer protein/seed storage 2S albumin superfamily protein (AHRD V3.3 *-* A0A2U1NA97_ARTAN)
Solyc10g083940.1	-1.357983361	5.75395893	0.00024339	0.03155958	Major facilitator superfamily (AHRD V3.3 *** A0A2U1KPS1_ARTAN)
Solyc05g026245.1	3.760452755	0.78758311	0.00024593	0.03172562	Retrovirus-related Pol polyprotein from transposon TNT 1-94 (AHRD V3.3 *** A0A2I0VN15_9ASPA)
Solyc08g077060.3	3.184730003	1.72130594	0.00025016	0.03210807	Zinc finger, LSD1-type (AHRD V3.3 *** A0A2U1LIJ4_ARTAN)
Solyc07g049135.1	-1.433021683	13.1434964	0.00025707	0.03282807	Fruit-specific protein (AHRD V3.3 *** FSPM_SOLLC)
Solyc11g068430.3	1.431016661	6.14462796	0.00026041	0.03308809	Ferredoxin (AHRD V3.3 *** K4DA01_SOLLC)
Solyc06g075660.4	1.926757271	1.52464408	0.00026398	0.03337305	MYB transcription factor (AHRD V3.3 *-* B4FPU3_MAIZE)
Solyc03g113130.3	1.833974651	2.35594253	0.00027308	0.03369379	plant cysteine oxidase 2-like (AHRD V3.3 *** A0A2G3A1M0_CAPAN)
Solyc10g084770.3	1.386249845	2.65242162	0.00027085	0.03369379	Protein kinase APK1B, chloroplastic (AHRD V3.3 *** A0A1U8E7T5_CAPAN)
Solyc06g071990.4	1.146941602	4.95229872	0.00027451	0.03369379	P-loop containing nucleoside triphosphate hydrolases superfamily protein (AHRD V3.3 *-* A0A2U1N2C4_ARTAN)
Solyc09g008010.3	1.10225142	6.0450536	0.00027072	0.03369379	Protein kinase (AHRD V3.3 *** Q9M7J5_ELYEL)
Solyc11g006290.2	-1.839365608	2.63394419	0.0002718	0.03369379	3-oxo-5-alpha-steroid 4-dehydrogenase (AHRD V3.3 *** A0A200PYH0_9MAGN)
Solyc01g105770.2	-3.389336967	1.27366584	0.0002741	0.03369379	IgA FC receptor (AHRD V3.3 *-* A0A1J3K238_NOCCA)

Solyc10g050980.1	2.313982258	0.28443716	0.00027654	0.03377873	Unknown protein
Solyc04g082140.3	1.591285218	5.3052355	0.0002861	0.03477961	pectinesterase
Solyc01g106620.2	-3.579418331	5.0128744	0.00028799	0.03484144	pathogenesis-related protein 1-like (AHRD V3.3 *** A0A2I4HRD5_9ROSI)
Solyc12g009730.3	-5.395981299	-0.8558344	0.00030281	0.03645942	receptor-like protein 12 (AHRD V3.3 *** A0A1S4DP57_TOBAC)
Solyc01g098490.3	-1.11694604	3.88755841	0.00030461	0.03650278	Sugar facilitator protein 1
Solyc03g115370.3	0.930534942	7.37696936	0.000307	0.0366154	Diacylglycerol kinase (AHRD V3.3 *** A0A1U8G5K6_CAPAN)
Solyc02g089630.3	2.135025972	5.80127552	0.00031087	0.03675892	Proline dehydrogenase (AHRD V3.3 *** A0A2G2XIU2_CAPBA)
Solyc03g093510.2	1.416631769	5.07156189	0.00031111	0.03675892	Alpha/beta-Hydrolases superfamily protein (AHRD V3.3 *** A0A2U1NKR4_ARTAN)
Solyc09g008280.2	1.310311306	8.59195065	0.00031993	0.0369689	S-adenosyl-L-methionine synthetase Z24743
Solyc02g092000.3	1.285777328	5.18368502	0.0003202	0.0369689	transmembrane protein (AHRD V3.3 *** AT3G49720.3)
Solyc03g007310.3	1.260367203	3.64856142	0.00031595	0.0369689	Abscisic acid receptor PYL8 (AHRD V3.3 *** A0A2G3CWW8_CAPCH)
Solyc08g075870.3	1.062347626	8.44399945	0.00031562	0.0369689	S-adenosyl-L-methionine-dependent methyltransferases superfamily protein (AHRD V3.3 *** A0A2U1NU97_ARTAN)
Solyc08g081190.3	-1.084236303	10.3823987	0.00031853	0.0369689	plasma membrane intrinsic protein 1.5
Solyc03g114450.3	-1.269623668	5.31797816	0.00033298	0.03826952	Calcium sensing receptor, chloroplastic (AHRD V3.3 *** A0A2G2WRE3_CAPBA)
Solyc02g077040.4	0.960233496	9.5098616	0.00033705	0.03856268	phytophthora-inhibited protease 1
Solyc07g063520.3	1.176148618	6.24418098	0.00033863	0.03856928	Organic solute transporter ostalpha protein (DUF300) (AHRD V3.3 *** O65422_ARATH)
Solyc06g009270.3	1.469974216	4.64294738	0.00034533	0.03898025	Diacylglycerol kinase (AHRD V3.3 *** A0A2G3ATC8_CAPCH)
Solyc04g072450.3	1.01375013	4.23654501	0.00034382	0.03898025	acidic leucine-rich nuclear phosphoprotein 32 family member B (AHRD V3.3 *-* XP_010320137.1)
Solyc03g097270.3	0.948777085	7.4853627	0.00035095	0.03943846	cystatin 9
Solyc05g024260.3	-0.912338639	8.32870228	0.00035713	0.03980095	Bidirectional sugar transporter SWEET (AHRD V3.3 *** A0A2G3C5C6_CAPCH)
Solyc12g044440.2	-4.102059848	0.29305836	0.00035732	0.03980095	Zinc finger protein (AHRD V3.3 *-* A0A200QX85_9MAGN)
Solyc04g082010.1	-1.401628001	5.36897484	0.00035917	0.03983132	pre-plastocyanin
Solyc02g063220.3	1.718048377	2.8119095	0.00036426	0.04008229	Mannose-6-phosphate isomerase (AHRD V3.3 *** A0A2G2XBW4_CAPBA)
Solyc01g105767.1	-4.247744606	0.42523022	0.0003646	0.04008229	hydroxyproline-rich glycoprotein family protein (AHRD V3.3 *-* AT5G09530.1)
Solyc12g006850.2	1.8137441	4.73613783	0.00037237	0.04064642	LELKT1GEN L.esculentum potassium channel
Solyc03g120420.3	-1.10434905	8.13447426	0.00037295	0.04064642	Unknown protein
Solyc01g095150.3	1.047200643	8.76710284	0.00038349	0.04161594	late embryogenesis-like protein
Solyc01g109660.2	0.990063071	10.1708059	0.0003913	0.0422423	meloidogyne-induced giant cell protein DB275
Solyc08g079900.3	-5.004910593	-0.0092057	0.0003926	0.0422423	Subtilisin-like protease (AHRD V3.3 *** Q9LWA4_SOLLC)
Solyc06g009890.2	2.436053103	0.36011313	0.00040397	0.04321693	Unknown protein
Solyc02g090120.1	2.329533724	4.16125203	0.00040749	0.04321693	hypothetical protein (AHRD V3.3 -** AT5G66985.1)
Solyc10g086690.3	1.438356515	5.42842323	0.00040862	0.04321693	Phosphatidylinositol:ceramide inositolphosphotransferase (AHRD V3.3 *-* A0A2G3BFT1_CAPCH)
Solyc09g010800.5	-0.893743983	12.443239	0.00041021	0.04321693	Metallothionein-like protein type 2 (AHRD V3.3 *** A0A2G2VXD7_CAPBA)
Solyc04g051800.3	-1.370754647	8.59854822	0.0004072	0.04321693	ABC transporter-like (AHRD V3.3 *** A0A200QC97_9MAGN)
Solyc06g083310.3	1.089228835	7.6794874	0.00042649	0.04456121	Hexosyltransferase (AHRD V3.3 *** A0A1U8H237_CAPAN)

Solyc05g013510.4	-1.434715762	5.70978111	0.00042623	0.04456121	Phosphate transporter (AHRD V3.3 *** Q6J2Q8_SOLTU)
Solyc10g050325.2	4.254273805	0.63759632	0.0004306	0.04462943	Kinesin-like calmodulin-binding protein (AHRD V3.3 *-* A0A2G3A688_CAPAN)
Solyc01g087970.3	1.738617095	0.97977288	0.00043067	0.04462943	Carboxypeptidase (AHRD V3.3 *** A0A2G2XQG5_CAPBA)
Solyc12g096940.3	5.35681128	-0.94705	0.00043444	0.0448361	CCT motif family protein (AHRD V3.3 *** A0A2U1QLU6_ARTAN)
Solyc07g062600.4	1.124453083	6.16130726	0.00044094	0.04532149	Acyl-CoA N-acyltransferase with RING/FYVE/PHD-type zinc finger protein (AHRD V3.3 *-* A0A1I9LNP0_ARATH)
Solyc08g006770.3	1.743455647	5.90954496	0.0004682	0.04773554	2-oxoglutarate (2OG) and Fe(II)-dependent oxygenase superfamily protein (AHRD V3.3 *** A0A2U1MCV6_ARTAN)
Solyc06g068090.3	1.133716838	8.04605475	0.00046723	0.04773554	phospholipase PLDa1
Solyc07g007160.3	-1.387303773	3.82270408	0.0004701	0.04773661	RING/U-box superfamily protein (AHRD V3.3 *-* A0A1P8AQV3_ARATH)
Solyc01g005140.3	1.101754718	6.39549947	0.0004801	0.04855745	Cytochrome b561/ferric reductase transmembrane (AHRD V3.3 *** A0A2U1MNP8_ARTAN)
Solyc10g085230.2	-0.993072038	10.5968747	0.00048736	0.04909535	ripening-related mRNA 1b
Solyc03g111170.3	1.280081956	4.71584017	0.00049482	0.04948051	4-coumarate-CoA ligase (AHRD V3.3 *** E5GBV5_CUCME)
Solyc06g072710.3	-1.786038882	1.86013785	0.0004951	0.04948051	RNA polymerase sigma factor sigA (AHRD V3.3 *** A0A2G2ZD00_CAPAN)
Solyc02g091630.1	1.450661601	2.5404368	0.00050113	0.04988595	DNA mismatch repair protein MLH3 (AHRD V3.3 *-* A0A2G2XFY3_CAPBA)
Solyc03g078150.3	-1.325277571	3.45380048	0.00050521	0.04993057	Amino acid transporter family protein (AHRD V3.3 *** S8E0T7_9LAMI)
Solyc09g060100.3	-1.601538607	1.95202585	0.00050553	0.04993057	transmembrane protein (AHRD V3.3 *** AT4G13150.1)

Table S3. List of DEGs showing FDR < 0.05 in the comparison of NtLuc/MT and MT/MT.

Gene	logFC: (NtLuc/MT) / (MT/MT)	logCPM	PValue	FDR	Description
Solyc04g078880.3	2.250773279	4.02530056	3.92E-13	4.95E-09	cold regulated protein 27 (AHRD V3.3 *** AT5G42900.2)
Solyc01g095140.4	3.602261635	8.11864867	2.71E-13	4.95E-09	Desiccation protectant protein Lea14-like protein (AHRD V3.3 *** A0A2G3BT76_CAPCH)
Solyc10g055810.2	-3.793829446	5.35081871	5.88E-12	4.96E-08	chitinase Z15140
Solyc04g064690.4	6.388157804	3.31924724	1.01E-11	6.39E-08	Peroxidase (AHRD V3.3 *** K4BT60_SOLLC)
Solyc02g081030.4	2.899339743	7.8851557	2.88E-11	1.46E-07	ERD (Early-responsive to dehydration stress) family protein (AHRD V3.3 *-* A0A2U1NFB6_ARTAN)
Solyc04g082200.2	2.085263418	10.8903961	1.47E-10	6.18E-07	dehydrin
Solyc10g055800.2	-3.050067643	5.02303855	1.73E-10	6.24E-07	Chitinase (AHRD V3.3 *** B9VRK7_CAPAN)
Solyc08g066705.1	-2.142039299	5.62378973	2.34E-10	7.40E-07	Gag-pol polyprotein (AHRD V3.3 *** E6Y5Q4_SOLLC)
Solyc02g014860.3	2.323002819	6.44521407	4.61E-10	1.29E-06	Chaperone protein DnaJ (AHRD V3.3 *** A0A1J3ID64_NOCCA)
Solyc08g080650.3	-3.315060601	5.73292628	1.44E-09	3.03E-06	PATHOGENESIS RELATED PROTEIN P23
Solyc02g085400.3	2.588599577	3.60381527	1.25E-09	3.03E-06	Sulfite exporter TauE/SafE family protein (AHRD V3.3 *** A0A2U1KY14_ARTAN)
Solyc05g053080.2	2.924704015	6.27225079	1.34E-09	3.03E-06	Unknown protein
Solyc05g024010.3	1.665940852	4.90577472	1.96E-09	3.80E-06	Zinc finger protein CONSTANS-LIKE 15 (AHRD V3.3 *** A0A2G2VMU9_CAPBA)
Solyc12g100270.2	-5.225116611	2.35561798	4.61E-09	8.32E-06	Fatty acid hydroxylase superfamily (AHRD V3.3 *** A0A2U1L8N4_ARTAN)
Solyc09g075300.3	2.002571786	3.27174464	5.76E-09	9.70E-06	Epoxide hydrolase 2 (AHRD V3.3 *** B6T857_MAIZE)
Solyc05g053070.4	2.856712345	7.44090976	7.18E-09	1.13E-05	Unknown protein
Solyc03g019690.1	-4.634655114	1.89074205	8.65E-09	1.29E-05	Serine protease inhibitor 1 (AHRD V3.3 *** A0A2G2XWH1_CAPAN)
Solyc03g080190.3	3.65187658	4.40074106	9.15E-09	1.29E-05	2-oxoglutarate (2OG) and Fe(II)-dependent oxygenase superfamily protein (AHRD V3.3 *** A0A2U1QGX7_ARTAN)
Solyc04g072020.3	1.880571376	5.05079524	1.19E-08	1.58E-05	Choline/Ethanolamine kinase (AHRD V3.3 *** A0A200QCA0_9MAGN),Pfam:PF01633
Solyc12g056678.1	-7.066687023	0.31227182	1.29E-08	1.63E-05	Alpha/beta-hydrolases superfamily protein (AHRD V3.3 *** A0A2U1LX98_ARTAN)
Solyc06g076350.3	2.713800452	4.32704583	1.58E-08	1.90E-05	LePCL1
Solyc02g064620.2	8.311959648	1.3313403	1.68E-08	1.94E-05	ARM repeat superfamily protein (AHRD V3.3 *** A0A2U1MQ52_ARTAN)
Solyc12g044950.3	-5.026833776	0.63038846	3.25E-08	3.57E-05	lipid desaturase
Solyc12g150125.1	-6.690236228	1.41116738	3.51E-08	3.65E-05	Alpha/beta-hydrolases superfamily protein (AHRD V3.3 *** A0A2U1LX98_ARTAN)
Solyc01g059965.1	-4.289115413	5.21222356	3.67E-08	3.65E-05	Glucan endo-1,3-beta-glucosidase B (AHRD V3.3 *** E13B_SOLLC)
Solyc01g079610.3	1.449336974	4.59506842	3.75E-08	3.65E-05	DnaJ protein ERDJ3B (AHRD V3.3 *** A0A2G2Z6I0_CAPAN)
Solyc04g081960.1	3.5711609	4.31258986	4.15E-08	3.89E-05	Syringolide-induced protein 14-1-1 (AHRD V3.3 *** A0A2K3M7L2_TRIPR)
Solyc09g007010.1	-3.538152773	6.03313148	4.64E-08	4.19E-05	Pathogenesis-related protein 1 (AHRD V3.3 *** Q75QH2_CAPCH)
Solyc07g064160.3	1.97900669	7.59462566	5.09E-08	4.44E-05	Thiamine thiazole synthase, chloroplastic (AHRD V3.3 *** A0A2G2WFN7_CAPBA),Pfam:PF01946
Solyc10g079860.2	-4.930698679	2.85683328	7.27E-08	5.88E-05	LEQB L.esculentum TomQ'b beta(1,3)glucanase
Solyc10g017980.1	-2.861857374	5.80946511	7.36E-08	5.88E-05	Chitinase (AHRD V3.3 *-* B8QVJ5_ZEAMP)
Solyc12g087950.1	2.534008638	4.64969464	7.45E-08	5.88E-05	AT-hook motif nuclear-localized protein (AHRD V3.3 *** A0A1U8GD78_CAPAN)

Solyc06g073080.4	3.31217654	2.05683467	8.19E-08	6.28E-05	2-oxoglutarate (2OG) and Fe(II)-dependent oxygenase superfamily protein (AHRD V3.3 *** A0A2U1QGX7_ARTAN)
Solyc10g075150.2	-2.569027778	4.88398052	1.18E-07	8.77E-05	Non-specific lipid-transfer protein (AHRD V3.3 *** A0A2G2YKI8_CAPAN)
Solyc09g006005.1	-3.353754435	3.98170187	1.32E-07	9.47E-05	Pathogenesis-related protein 1 (AHRD V3.3 *** Q75QH2_CAPCH)
Solyc10g081170.2	1.777665169	9.00890758	1.35E-07	9.47E-05	Calmodulin 2
Solyc00g500191.1	-4.327903197	0.37438177	1.44E-07	9.83E-05	Protein TIC 214 (AHRD V3.3 *-* TI214_SOLTU)
Solyc10g017970.1	-2.748311969	6.16132068	1.91E-07	0.0001269	Chitinase (AHRD V3.3 *-* B8QVJ5_ZEAMP)
Solyc03g115770.3	1.843346952	5.55790394	2.51E-07	0.00016267	Two-component response regulator-like APRR5 (AHRD V3.3 *** A0A2G3CZB4_CAPCH)
Solyc10g055820.3	-4.526587747	1.61058752	3.19E-07	0.00019677	Chitinase (AHRD V3.3 *** B9VRK7_CAPAN)
Solyc11g066320.2	1.236979984	4.7449139	3.14E-07	0.00019677	Exostosin family protein (AHRD V3.3 *** A0A2U1P7C1_ARTAN)
Solyc04g071900.4	-2.834120182	2.75065178	3.43E-07	0.00020675	Peroxidase (AHRD V3.3 *** K4BTH7_SOLLC)
Solyc04g040130.1	-6.472192631	1.18373502	4.60E-07	0.00027053	Fatty acid desaturase (AHRD V3.3 *** E7CCD0_CAMMC)
Solyc10g076710.3	-2.515579381	1.46166837	4.74E-07	0.00027219	Phosphoinositide phospholipase C (AHRD V3.3 *** O49950_SOLTU)
Solyc01g106920.4	-4.363227726	0.49027161	5.15E-07	0.00028594	Nucleobase-ascorbate transporter 8 (AHRD V3.3 *** A0A1U8DZ27_CAPAN)
Solyc06g005680.4	4.398197361	2.39384655	5.20E-07	0.00028594	Two-component response regulator (AHRD V3.3 *-* C0HE02_MAIZE)
Solyc08g082210.4	2.218133662	4.39708156	5.89E-07	0.00031689	Ethylene-responsive transcription factor (AHRD V3.3 *** A0A2G3ADG6_CAPAN)
Solyc01g081250.3	-2.804749481	3.12319939	6.52E-07	0.00034336	Glutathione S-transferase (AHRD V3.3 *** A0A200PVK3_9MAGN)
Solyc08g078870.3	2.361590192	3.43437835	6.95E-07	0.00035848	Bifunctional inhibitor/lipid-transfer protein/seed storage 2S albumin superfamily protein (AHRD V3.3 *** A0A2U1PU47_ARTAN)
Solyc01g094370.3	1.331314736	6.68112984	9.44E-07	0.00047729	remorin 2
Solyc12g056675.1	-5.447474996	4.58623912	1.09E-06	0.00054254	Alpha/beta-hydrolases superfamily protein (AHRD V3.3 *** A0A2U1LX98_ARTAN)
Solyc07g062700.3	1.933646493	9.69501933	1.18E-06	0.00057511	Calcium-binding EF-hand family protein (AHRD V3.3 *** A0A2U1MZJ3_ARTAN)
Solyc12g005910.2	1.734652227	5.82798669	1.22E-06	0.00058016	B-cell receptor-associated 31-like (AHRD V3.3 *** A0A200QH03_9MAGN)
Solyc08g075370.3	1.766152068	7.98911187	1.40E-06	0.00065612	Unknown protein
Solyc02g077110.3	2.090177881	3.38119585	1.50E-06	0.00069119	Phospholipase A1-II 1 (AHRD V3.3 *** A0A2G2XE87_CAPBA)
Solyc09g097760.3	-4.341219244	3.01623391	1.57E-06	0.00071012	Glycine-rich protein (AHRD V3.3 *-* A0A2G2W2G9_CAPBA)
Solyc07g063520.3	1.716411388	6.6296619	1.87E-06	0.00082791	Organic solute transporter ostalpha protein (DUF300) (AHRD V3.3 *** O65422_ARATH)
Solyc01g088560.3	1.35320205	7.20106085	2.06E-06	0.0008984	Rab family GTPase (AHRD V3.3 *** A0A1Y1IFP9_KLENI)
Solyc01g097340.3	-1.622536142	5.65760602	2.35E-06	0.00100595	GDP-mannose-3',5'-epimerase (AHRD V3.3 *** A5JPK5_VITVI)
Solyc04g051360.3	5.449668757	4.3308328	2.42E-06	0.00102021	Ethylene Response Factor D.1
Solyc01g105350.3	-2.281617045	2.06496009	2.49E-06	0.00103243	Glycosyltransferase (AHRD V3.3 *** A0A1Q3B719_CEPFO)
Solyc06g069760.3	-1.634354773	5.35085067	2.65E-06	0.00106197	Dof zinc finger protein (AHRD V3.3 *** A0A2K3MQ38_TRIPR)
Solyc01g095970.3	1.772579118	6.01702899	2.63E-06	0.00106197	Dynamin-like protein (AHRD V3.3 *** F4K015_ARATH)
Solyc05g012580.1	1.784456546	3.69976812	2.78E-06	0.00109946	Unknown protein
Solyc02g089350.3	-1.773866619	3.78307066	2.94E-06	0.00114558	Gibberellin regulated protein (AHRD V3.3 *** A0A2U1PE48_ARTAN)
Solyc12g100250.3	-4.213190821	0.90597714	3.16E-06	0.00120927	Fatty acid desaturase (AHRD V3.3 *** A0A200QRI1_9MAGN)
Solyc08g080500.3	1.343831324	7.11180532	3.30E-06	0.00124716	Rhomboid-like protein (AHRD V3.3 *** A0A1U8G8L0_CAPAN)

Solyc01g111520.3	1.120358568	7.13976341	3.60E-06	0.00133817	Calcium-dependent lipid-binding (CaLB domain) family protein (AHRD V3.3 *** A0A1P8BD17_ARATH)
Solyc02g068430.4	1.345685746	7.38394803	4.17E-06	0.00152647	Choline-phosphate cytidylyltransferase (AHRD V3.3 *** A0A2I0V8H3_9ASPA)
Solyc11g008720.3	-1.771559039	3.63377588	4.46E-06	0.00158777	Beta-glucosidase (AHRD V3.3 *** A0A2G2VEH4_CAPBA)
Solyc06g063060.3	1.710052354	3.9492964	4.42E-06	0.00158777	Dormancy/auxin associated protein (AHRD V3.3 *-* A0A2K3JP12_TRIPR)
Solyc07g043230.3	2.56273447	3.18237356	4.58E-06	0.0016073	Zinc transporter protein (AHRD V3.3 *** A5BDR3_VITVI)
Solyc07g006900.2	3.791172709	2.39106069	4.81E-06	0.00166579	SlPIN2
Solyc06g009270.3	1.841238505	4.91874822	4.89E-06	0.00167104	Diacylglycerol kinase (AHRD V3.3 *** A0A2G3ATC8_CAPCH)
Solyc08g068610.3	-4.009214348	0.58798149	5.15E-06	0.00173469	Serine decarboxylase (AHRD V3.3 *** A0A2G2W5V8_CAPBA)
Solyc12g098920.2	-1.487964201	4.8985748	5.31E-06	0.00174461	Guanosine-3'5'-bis(Diphosphate) 3'-pyrophosphohydrolase (AHRD V3.3 *** E5GC47_CUCME)
Solyc03g095780.3	4.68190311	0.56243331	5.30E-06	0.00174461	abscisic acid receptor PYL4-like (AHRD V3.3 *** A0A2I4EQL0_9ROSI)
Solyc09g008280.2	1.657922745	8.8406402	5.39E-06	0.00174804	S-adenosyl-L-methionine synthetase Z24743
Solyc09g098510.3	-2.557843752	3.64004694	5.53E-06	0.00176917	Extensin-3 (AHRD V3.3 *-* A0A2G2ZT41_CAPAN)
Solyc04g080300.4	-1.287622314	5.13091446	5.64E-06	0.00178333	Erythronate-4-phosphate dehydrogenase family protein (AHRD V3.3 *** Q8VYC6_ARATH)
Solyc07g009230.3	-4.055782548	1.93092826	6.29E-06	0.00187005	Defensin-like protein 1 (AHRD V3.3 *** A0A2G2ZS54_CAPAN)
Solyc10g085240.1	-2.93876033	3.41777969	6.16E-06	0.00187005	UDP-glycosyltransferase 76E1 (AHRD V3.3 *** U76E1_SOLLC)
Solyc02g062180.3	-1.351213711	4.97356527	6.02E-06	0.00187005	2-oxoglutarate (2OG) and Fe(II)-dependent oxygenase superfamily protein (AHRD V3.3 *-* A0A2U1N614_ARTAN)
Solyc01g108100.3	1.370741838	4.43791405	6.23E-06	0.00187005	cold regulated protein 27 (AHRD V3.3 *** AT5G42900.2)
Solyc05g009340.1	4.114452204	0.86510623	6.19E-06	0.00187005	Unknown protein
Solyc12g088230.3	1.126291132	7.73966214	6.96E-06	0.00202223	mitochondrial malate dehydrogenase
Solyc03g083620.1	1.214209838	8.59327185	6.92E-06	0.00202223	Unknown protein
Solyc05g012230.4	-2.818180935	1.58548429	7.37E-06	0.00211848	hypothetical protein (AHRD V3.3 *-* AT5G61040.1)
Solyc04g082210.3	1.218650046	4.85257531	7.51E-06	0.00213464	NADH-ubiquinone oxidoreductase chain (AHRD V3.3 *** AT1G76185.1)
Solyc09g090980.3	-1.975502877	5.79676768	7.70E-06	0.0021467	pathogenesis-related protein STH-2-like (AHRD V3.3 *** A0A1U7Y3H6_NICSY)
Solyc01g005140.3	1.272996755	6.50581602	7.73E-06	0.0021467	Cytochrome b561/ferric reductase transmembrane (AHRD V3.3 *** A0A2U1MNP8_ARTAN)
Solyc06g060340.3	-5.688473913	-0.6820663	8.11E-06	0.00222862	Photosystem II 22 kDa protein, chloroplastic (AHRD V3.3 *** A0A1U8GV91_CAPAN)
Solyc01g060020.4	-8.975471509	1.96816097	8.34E-06	0.00226874	beta-1,3-glucanase TOMB13GLUB
Solyc01g097770.4	-2.607680548	0.81869675	8.59E-06	0.00230857	phototropin 2
Solyc02g090310.1	1.66124468	5.76477137	8.67E-06	0.00230857	Dof zinc finger protein (AHRD V3.3 *** A0A2K3LFN5_TRIPR)
Solyc10g086180.2	-1.376297523	5.8151246	8.92E-06	0.00234861	Phenylalanine ammonia-lyase (AHRD V3.3 *** A0A2G2VU16_CAPBA)
Solyc09g063010.4	-1.565524449	5.27657337	1.00E-05	0.00260842	bHLH transcription factor 058
Solyc05g053610.2	-4.76470016	1.98409985	1.06E-05	0.0027197	Pleiotropic drug resistance protein (AHRD V3.3 *** C8CA13_CUCSA)
Solyc07g064720.3	2.821729535	3.89259583	1.06E-05	0.0027197	Gdsl esterase/lipase (AHRD V3.3 *** A0A2P4JQK3_QUESU)
Solyc03g115940.4	-1.784834518	2.94496053	1.13E-05	0.00285114	Dof zinc finger protein (AHRD V3.3 *** A0A2K3MQ38_TRIPR)
Solyc07g009530.1	-3.452752141	2.0727916	1.15E-05	0.00287271	Chitinase (AHRD V3.3 *-* B8QVH4_ZEAMP)
Solyc03g115370.3	1.183049452	7.54034649	1.16E-05	0.00287982	Diacylglycerol kinase (AHRD V3.3 *** A0A1U8G5K6_CAPAN)

Solyc09g090190.3	1.389941502	6.02902433	1.20E-05	0.00293804	S-adenosyl-L-methionine-dependent methyltransferases superfamily protein (AHRD V3.3 *** A0A2U1MED4_ARTAN)
Solyc10g085420.3	1.941703461	5.15375656	1.21E-05	0.00293804	Protein LURP-one-related 15 (AHRD V3.3 *** A0A2G2VU46_CAPBA)
Solyc10g076660.2	2.488434988	1.19178662	1.23E-05	0.00295266	2-oxoglutarate (2OG) and Fe(II)-dependent oxygenase superfamily protein (AHRD V3.3 *** F4J670_ARATH)
Solyc01g005300.4	1.047008248	7.02696152	1.27E-05	0.00302425	Adagio-like protein 1 (AHRD V3.3 *** A0A1U8H9V5_CAPAN)
Solyc06g036110.1	1.732909066	5.90749743	1.34E-05	0.00316755	Calcium-dependent lipid-binding (CaLB domain) family protein (AHRD V3.3 *** A0A2U1LCI8_ARTAN)
Solyc09g065440.4	-4.491634587	-0.3546603	1.42E-05	0.00333142	Bifunctional inhibitor/lipid-transfer protein/seed storage 2S albumin superfamily protein (AHRD V3.3 *-* A0A2U1NA97_ARTAN)
Solyc05g050010.3	-1.509525761	6.88170483	1.46E-05	0.00337615	1-aminocyclopropane-1-carboxylic acid synthase-4
Solyc04g015750.3	-1.66567647	8.24047527	1.50E-05	0.0034397	CobN/magnesium chelatase (AHRD V3.3 *** A0A200PZ28_9MAGN)
Solyc03g093510.2	2.205869011	5.67509742	1.51E-05	0.0034397	Alpha/beta-Hydrolases superfamily protein (AHRD V3.3 *** A0A2U1NKR4_ARTAN)
Solyc06g083310.3	1.305001309	7.82105945	1.52E-05	0.00344162	Hexosyltransferase (AHRD V3.3 *** A0A1U8H237_CAPAN)
Solyc06g051620.3	1.633160161	4.10992574	1.56E-05	0.0034807	Phosphoinositide phospholipase C (AHRD V3.3 *** O49951_SOLTU)
Solyc06g069470.3	1.175928538	5.41298234	1.74E-05	0.00382308	Glycerophosphodiester phosphodiesterase GDPD5 (AHRD V3.3 *** A0A2G2WM22_CAPBA)
Solyc12g009160.2	1.613988492	3.43462901	1.73E-05	0.00382308	Inter-alpha-trypsin inhibitor heavy chain-like protein (AHRD V3.3 *** Q8L798_ARATH)
Solyc06g068960.1	3.224922199	0.75599788	1.95E-05	0.004244	Calcium-binding allergen Ole e 8 (AHRD V3.3 *** A0A2G2WLM3_CAPBA)
Solyc04g083140.2	-4.297430185	0.76708365	1.97E-05	0.00426586	Cytochrome (AHRD V3.3 *** A0A1U8F0H8_CAPAN)
Solyc03g078090.4	4.60495248	1.30018592	2.06E-05	0.00441806	Pectinesterase (AHRD V3.3 *** A0A2I4GT13_9ROSI)
Solyc12g088460.3	-1.4501743	6.5580733	2.13E-05	0.00451636	Cytochrome P450 (AHRD V3.3 *** A0A200Q0W8_9MAGN)
Solyc02g089060.3	1.089437545	4.79286909	2.19E-05	0.00456833	Methyl esterase 11 (AHRD V3.3 *** A0A2U1LRP6_ARTAN)
Solyc05g052450.3	1.574644342	2.76095039	2.18E-05	0.00456833	Protein trichome birefringence-like 32 (AHRD V3.3 *** A0A1U8GQI0_CAPAN)
Solyc05g050007.1	-1.334608886	4.66674252	2.24E-05	0.00464172	Serine/threonine-protein phosphatase 7 long form-like protein (AHRD V3.3 *-* A0A1J3IZ43_NOCCA)
Solyc12g044954.1	-5.691787091	1.1381992	2.40E-05	0.00489747	Cytochrome (AHRD V3.3 *** A0A2G2Y725_CAPAN)
Solyc01g007220.4	1.201948584	8.17210677	2.40E-05	0.00489747	Late embryogenesis abundant (LEA) hydroxyproline-rich glycoprotein family (AHRD V3.3 *** Q9SVC8_ARATH)
Solyc01g102610.3	-2.101275456	6.41541335	2.49E-05	0.00504373	Ferric reduction oxidase 6 (AHRD V3.3 *** A0A2G3BUN6_CAPCH)
Solyc06g068090.3	1.456227789	8.26912424	2.61E-05	0.00523543	phospholipase PLDa1
Solyc09g090990.2	-3.558204531	1.59135738	2.71E-05	0.00539112	Major allergen Pru ar 1 (AHRD V3.3 *** Q5GMN2_CAPCH)
Solyc01g095530.2	-1.757049111	2.86767241	2.82E-05	0.00552726	hypothetical protein (AHRD V3.3 *-* AT5G41761.1)
Solyc04g008210.2	1.135371141	6.01294569	2.80E-05	0.00552726	ETAG-A3
Solyc07g061950.4	1.608495622	5.97642696	3.04E-05	0.00590879	C2 domain-containing protein (AHRD V3.3 *** A0A2G2WER0_CAPBA)
Solyc02g091250.1	1.893108603	3.93947914	3.09E-05	0.00597275	Mediator of RNA polymerase II transcription subunit (AHRD V3.3 *** Q9ZU37_ARATH)
Solyc03g116590.3	1.112443531	7.73901121	3.13E-05	0.00600085	Embryo-specific protein (AHRD V3.3 *** A0A2K3LFD9_TRIPR)
Solyc04g081650.3	2.101118288	2.11785806	3.20E-05	0.00608114	Cyclin (AHRD V3.3 *-* A0A200PQW1_9MAGN)
Solyc06g082240.2	-3.964155613	1.69994848	3.29E-05	0.00621155	Laccase (AHRD V3.3 *** K4CA83_SOLLC)

Solyc08g082590.3	-3.517725606	0.42821439	3.42E-05	0.00636828	Glutaredoxin (AHRD V3.3 *** A0A2U1KGP9_ARTAN)
Solyc04g016470.4	-1.742700168	4.58718965	3.43E-05	0.00636828	LEQA L.esculentum TomQ'a beta(1,3)glucanase
Solyc05g050770.4	-3.016530077	-0.1999263	3.46E-05	0.00638612	Serine carboxypeptidase (AHRD V3.3 *** A0A2K3LD91_TRIPR)
Solyc02g086900.4	1.35113351	5.2571518	3.49E-05	0.00638612	HD domain-containing metal-dependent phosphohydrolase family protein (AHRD V3.3 *** A0A2U1PYT8_ARTAN)
Solyc07g063620.3	1.216917042	7.09836402	3.52E-05	0.00640081	Membrane lipoprotein lipid attachment site-like protein, putative (DUF1223) (AHRD V3.3 *** O81838_ARATH)
Solyc01g106620.2	-3.909163795	4.98385004	3.61E-05	0.00649167	pathogenesis-related protein 1-like (AHRD V3.3 *** A0A2I4HRD5_9ROSI)
Solyc01g109660.2	1.112491884	10.2461125	3.62E-05	0.00649167	meloidogyne-induced giant cell protein DB275
Solyc09g009530.4	1.437608582	3.77814495	3.71E-05	0.00661007	Alpha/beta-Hydrolases superfamily protein (AHRD V3.3 *** Q9SJM9_ARATH)
Solyc04g071070.2	-2.861893783	4.12621509	3.80E-05	0.00666712	Unknown protein
Solyc05g053550.3	-1.514604166	7.97159242	3.78E-05	0.00666712	chalcone synthase 2
Solyc07g044960.1	3.089456713	3.8812641	4.07E-05	0.00709313	Xyloglucan galactosyltransferase KATAMARI1 (AHRD V3.3 *** A0A2K3L4H3_TRIPR)
Solyc09g091580.3	-1.450523283	7.2453823	4.12E-05	0.0071356	Protein kinase domain (AHRD V3.3 *** A0A200QUN4_9MAGN)
Solyc06g075660.4	3.130839261	2.49166579	4.27E-05	0.00734722	MYB transcription factor (AHRD V3.3 *-* B4FPU3_MAIZE)
Solyc08g075870.3	1.474559442	8.72703988	4.42E-05	0.00749342	S-adenosyl-L-methionine-dependent methyltransferases superfamily protein (AHRD V3.3 *** A0A2U1NU97_ARTAN)
Solyc06g066800.2	2.477203194	6.00921007	4.40E-05	0.00749342	Nucleotide-diphospho-sugar transferases superfamily protein (AHRD V3.3 *** AT1G64980.3)
Solyc04g082140.3	2.177446894	5.75733522	4.49E-05	0.00757385	pectinesterase
Solyc12g006850.2	1.90007326	4.80116939	4.90E-05	0.00820337	LELKT1GEN L.esculentum potassium channel
Solyc04g080040.3	-2.630257035	0.6014908	5.24E-05	0.00872103	Auxilin-related protein 2 (AHRD V3.3 *** A0A2G3CQ94_CAPCH)
Solyc04g072033.1	-1.30172454	5.67403862	5.40E-05	0.00891753	NAD(P)-binding Rossmann-fold superfamily protein (AHRD V3.3 *** Q9SZ91_ARATH)
Solyc06g060710.4	2.506614509	4.44106733	5.78E-05	0.00949626	Glyco_transf_8 domain-containing protein (AHRD V3.3 *** A0A1Q3D6P8_CEPFO)
Solyc03g020030.3	-4.534904762	0.24623956	6.22E-05	0.01007563	Proteinase inhibitor type-2 (AHRD V3.3 *** A0A2G3A0T9_CAPAN)
Solyc02g070530.4	2.271953842	1.18091597	6.19E-05	0.01007563	Potassium channel KAT1 (AHRD V3.3 *** A0A2G3ANB5_CAPAN)
Solyc05g053970.2	-6.13624362	-0.3865254	6.28E-05	0.01011892	Amino acid transporter (AHRD V3.3 *** A0A200QES7_9MAGN)
Solyc10g081570.3	1.841454909	9.21028707	6.56E-05	0.01049216	Marmande
Solyc07g063320.3	1.874173171	3.18289092	6.74E-05	0.01071735	LanC-like protein GCR2 (AHRD V3.3 *** A0A2G3C294_CAPCH)
Solyc02g092000.3	1.650376421	5.44252971	6.79E-05	0.0107312	transmembrane protein (AHRD V3.3 *** AT3G49720.3)
Solyc04g078640.3	1.094070286	6.12060648	6.84E-05	0.01074835	Ethylene-responsive transcription factor RAP2-1 (AHRD V3.3 *** A0A1U8GBU2_CAPAN)
Solyc07g053140.3	-2.218020996	5.58132872	7.31E-05	0.01126833	Zinc finger protein/CONSTANS-like protein (AHRD V3.3 *-* A0A2K3M573_TRIPR)
Solyc06g084770.2	-1.482331814	5.95266115	7.27E-05	0.01126833	cytochrome P450 CYP72A219 (AHRD V3.3 --* XP_004244272.1)
Solyc07g006500.3	1.057767628	6.77708149	7.26E-05	0.01126833	trehalose-6-phosphate synthase 1
Solyc06g066540.1	2.478668838	0.60341862	7.36E-05	0.01127987	Ethylene-responsive transcription factor (AHRD V3.3 *-* A0A2G2XMU8_CAPBA)
Solyc08g007830.1	3.633599047	2.85672803	7.60E-05	0.01157148	Dehydration-responsive element-binding protein 1E (AHRD V3.3 *** A0A1U8FGE9_CAPAN)
Solyc10g079370.3	1.118653907	6.3744338	7.68E-05	0.01163376	Transcription initiation factor IIB (AHRD V3.3 *** A0A1U8EBN6_CAPAN)
Solyc05g007880.4	-2.213670618	2.30137884	7.86E-05	0.01183109	cyclic dof factor 2-like (AHRD V3.3 *** A0A2I4E3P6_9ROSI)
Solyc09g091000.4	-3.076837883	3.02557477	8.06E-05	0.01206415	Pathogenesis-related protein STH-2 (AHRD V3.3 *** PRS2_SOLTU)

Solyc12g044230.2	2.007645362	5.13591279	8.28E-05	0.01231918	Sulfite exporter TauE/SafE family protein (AHRD V3.3 *** A0A2U1KY14_ARTAN)
Solyc01g088090.3	-1.388373013	8.21420999	8.42E-05	0.0123789	Pheophytinase, chloroplastic (AHRD V3.3 *** A0A2G3C5T3_CAPCH)
Solyc02g087110.3	-1.126120088	6.30553855	8.41E-05	0.0123789	Alpha-dioxygenase (AHRD V3.3 *** Q5GQ66_PEA)
Solyc04g040160.4	-2.505577808	0.50641238	8.65E-05	0.01264693	Protochlorophyllide-dependent translocon component 52, chloroplastic (AHRD V3.3 *** A0A2G2ZF87_CAPAN)
Solyc10g084600.2	-1.186280419	4.18932793	9.04E-05	0.01313576	Plant protein 1589 of Uncharacterized protein function (AHRD V3.3 *-* A0A061EZD3_THECC)
Solyc03g025850.3	0.904603416	7.61937662	9.28E-05	0.01340525	remorin 1
Solyc03g120710.2	0.97629552	6.35604935	9.50E-05	0.01364854	Late embryogenesis abundant protein (AHRD V3.3 *** A0A200PP54_9MAGN)
Solyc05g010330.4	-4.387789411	-0.4393417	9.64E-05	0.01377793	Peroxidase (AHRD V3.3 *** K4BXK6_SOLLC)
Solyc03g094020.3	4.284296664	0.25244106	9.78E-05	0.01388887	Alpha-glucosidase-like protein (AHRD V3.3 *** A0A2K3NWB8_TRIPR)
Solyc06g051400.3	-1.23992982	7.22412638	0.00010245	0.01447203	omega-3 fatty acid desaturase
Solyc08g013670.3	-3.011964336	1.05800762	0.00010571	0.0148496	Photosystem I reaction center subunit N, chloroplastic (AHRD V3.3 *** A0A1U8EZE3_CAPAN)
Solyc10g047320.2	1.472657984	3.93670279	0.0001084	0.01514334	Disease resistance protein (AHRD V3.3 *** A0A2U1N8H9_ARTAN)
Solyc09g008560.4	-3.417580638	-0.2113497	0.00011223	0.01559254	2-oxoglutarate (2OG) and Fe(II)-dependent oxygenase superfamily protein (AHRD V3.3 *** F4INZ9_ARATH)
Solyc05g054090.3	-1.926469888	2.27852887	0.00011349	0.01568087	induced stolen tip protein TUB8-like (AHRD V3.3 *** XP_004239829.1)
Solyc12g008940.2	1.108631468	7.57213925	0.00012067	0.0165823	Nucleosome assembly protein family (AHRD V3.3 *** A9TVZ4_PHYPA)
Solyc08g081610.4	6.099316265	0.74371856	0.00012377	0.01691661	WRKY family transcription factor (AHRD V3.3 *** A0A178V2E9_ARATH)
Solyc07g044980.3	1.130400795	5.12482264	0.00012572	0.01709005	NIM1-like protein 2
Solyc01g100000.3	2.001975169	2.77459545	0.0001269	0.01715836	F-box domain, Phloem protein 2-like protein (AHRD V3.3 *** A0A2U1MNC1_ARTAN)
Solyc07g049730.3	1.141225783	6.28000828	0.0001279	0.01720224	Transmembrane protein, putative (DUF1068) (AHRD V3.3 *** F4JGC5_ARATH)
Solyc02g083760.4	1.622218861	1.44155745	0.00012886	0.01723984	Thaumatin-like protein 1 (AHRD V3.3 *** A0A2G3D7H3_CAPCH)
Solyc02g086270.4	-1.700689168	2.18535627	0.00013143	0.01749043	Protein kinase family protein (AHRD V3.3 *** A0A2U1MH78_ARTAN)
Solyc01g095150.3	1.131971872	8.81681928	0.00013561	0.01795208	late embryogenesis-like protein
Solyc11g066860.2	-2.07566462	2.29533918	0.00013767	0.01813035	hypothetical protein (AHRD V3.3 *** AT2G27830.1)
Solyc08g075210.2	-1.11882076	5.98238989	0.00014032	0.01838375	HXXXD-type acyl-transferase family protein (AHRD V3.3 *** F4JBC7_ARATH)
Solyc03g117600.3	-1.484111407	3.00820811	0.00014139	0.01842742	Transferase (AHRD V3.3 *** A0A200QII6_9MAGN)
Solyc11g010100.2	0.995651702	5.42364632	0.00014736	0.01910736	Ras-related protein RABA4a (AHRD V3.3 *** A0A2G2Y594_CAPAN)
Solyc10g085230.2	-1.095554051	10.5530981	0.000151	0.0193924	ripening-related mRNA 1b
Solyc02g067180.3	-0.961942776	7.68967339	0.00015109	0.0193924	cystathionine gamma synthase
Solyc09g082690.3	-2.288903921	10.0896284	0.00015856	0.02004757	superoxide dismutase
Solyc12g013820.3	1.326028355	6.97382214	0.00015857	0.02004757	Ubiquitin-conjugating enzyme (AHRD V3.3 *-* A0A200QWS6_9MAGN)
Solyc07g062130.3	1.599773088	6.38547306	0.00015752	0.02004757	trifunctional UDP-glucose 4,6-dehydratase/UDP-4-keto-6-deoxy-D-glucose 3,5-epimerase/UDP-4-keto-L-rhamnose-reductase RHM1 (AHRD V3.3 *** A0A2I4GY66_9ROSI)
Solyc01g096620.4	-1.228399524	3.42135157	0.00015974	0.02009438	Cilia-and flagella-associated protein 20 (AHRD V3.3 *-* A0A2G2YYL7_CAPAN)
Solyc09g007770.3	-3.303430826	-0.3969454	0.00016295	0.0203545	plasma membrane intrinsic protein 2.1

Solyc04g081240.2	1.211919759	5.30965898	0.00016342	0.0203545	Auxin Response Factor 5
Solyc03g096545.1	-3.774638609	0.35473951	0.00016956	0.02101659	PLAT/LH2 domain-containing protein (AHRD V3.3 *** A0A2U1NEA8_ARTAN)
Solyc02g062610.3	-1.60983378	2.884797	0.0001729	0.02132573	Alpha/beta-Hydrolases superfamily protein (AHRD V3.3 *** O23227_ARATH)
Solyc01g081605.1	-1.117138271	5.82232412	0.00017803	0.02165883	Serine/threonine-protein phosphatase 7 long form-like protein (AHRD V3.3 *-* A0A1J3IZ43_NOCCA)
Solyc04g007130.1	1.118757275	5.17489403	0.00017775	0.02165883	hypothetical protein (AHRD V3.3 *** AT1G13360.1)
Solyc02g089500.4	1.281483764	4.40101007	0.00017817	0.02165883	Zinc finger protein CONSTANS-LIKE 2 (AHRD V3.3 *-* A0A2G3ABV8_CAPAN)
Solyc01g103650.3	-2.352990307	1.48827284	0.00018791	0.02273294	Alpha/beta-Hydrolases superfamily protein (AHRD V3.3 *** Q9XID7_ARATH)
Solyc06g068230.4	-1.442691036	5.67047238	0.00019037	0.02291703	Tetratricopeptide repeat (TPR)-like superfamily protein (AHRD V3.3 *** A0A2U1MYN5_ARTAN)
Solyc05g011890.1	-1.346174777	4.17029826	0.00019124	0.02291703	Sulfotransferase (AHRD V3.3 *** A0A2G3B6Y2_CAPCH)
Solyc12g049030.1	-4.029277357	1.74123847	0.00020565	0.02421165	Fatty acid desaturase (AHRD V3.3 *** A0A200QRI1_9MAGN)
Solyc09g091520.2	-2.332244818	0.16690193	0.00020478	0.02421165	60S acidic ribosomal protein P0 (AHRD V3.3 *** A0A2G3CRU5_CAPCH)
Solyc04g082420.3	0.895926984	7.52079102	0.00020408	0.02421165	BTB/POZ domain-containing protein (AHRD V3.3 *** A0A1U8GAB1_CAPAN)
Solyc09g007850.3	1.048871826	7.35364481	0.00020587	0.02421165	RNA-binding protein (AHRD V3.3 *** A0A2U1PI86_ARTAN)
Solyc11g072480.2	2.215645885	2.43581343	0.00021458	0.02511901	Tetraspanin-3 (AHRD V3.3 *** A0A2G3BA66_CAPCH)
Solyc04g081350.3	1.52493358	3.04712523	0.00021698	0.02528281	Transcription factor E2FC (AHRD V3.3 *** M5BC45_TOBAC)
Solyc05g051750.3	-1.753683933	1.97888888	0.00021943	0.02533492	TOMPRORNA prosystemin
Solyc10g086690.3	1.794235159	5.69594252	0.00021865	0.02533492	Phosphatidylinositol:ceramide inositolphosphotransferase (AHRD V3.3 *-* A0A2G3BFT1_CAPCH)
Solyc02g089510.3	1.401101215	5.29219414	0.00022226	0.02542886	Unknown protein
Solyc03g007710.3	2.053967442	2.13721052	0.00022127	0.02542886	membrane-associated kinase regulator (AHRD V3.3 *** AT5G52870.2)
Solyc10g006860.4	-1.221755289	6.04750213	0.00022784	0.02594981	NAD(P)-binding Rossmann-fold superfamily protein (AHRD V3.3 *** F4IPI3_ARATH)
Solyc07g052370.4	-5.73662871	-0.6662496	0.00023561	0.02652135	Cytochrome 71D7 (AHRD V3.3 *** A0A2G2WD66_CAPBA)
Solyc03g097270.3	1.000399065	7.51394889	0.00023915	0.02652135	cystatin 9
Solyc09g011660.3	1.209560326	5.16120052	0.00023681	0.02652135	Adenine nucleotide alpha hydrolases-like superfamily protein (AHRD V3.3 *** A0A2U1NYH2_ARTAN)
Solyc02g089520.2	1.286250869	4.81186117	0.00023465	0.02652135	Zinc finger protein CONSTANS-LIKE 2 (AHRD V3.3 *** A0A2G3ABV8_CAPAN)
Solyc09g065200.4	1.307418125	3.23865944	0.00023905	0.02652135	cyclinU3_1
Solyc02g078150.4	1.803279555	3.87968396	0.00023737	0.02652135	PDDEXK-like protein (AHRD V3.3 *** A0A2U1QIN2_ARTAN)
Solyc09g014350.3	-5.509485645	-0.7873482	0.0002427	0.02679815	Glycerol-3-phosphate acyltransferase, expressed (AHRD V3.3 *** D8L9N9_WHEAT)
Solyc11g005240.1	3.816690699	-0.0875199	0.00024688	0.02714045	protein SENSITIVITY TO RED LIGHT REDUCED 1-like (AHRD V3.3 *-* A0A2I4GZX1_9ROSI)
Solyc06g008920.3	-1.369045662	6.41888356	0.00026125	0.02859655	AMP-dependent synthetase/ligase (AHRD V3.3 *** A0A200QNF3_9MAGN)
Solyc04g074020.2	-2.757135021	0.39168423	0.00026278	0.02863955	Receptor protein kinase, putative (AHRD V3.3 *** A0A061FG24_THECC)
Solyc12g006050.2	2.282306751	2.12297025	0.00026461	0.02871532	Protein NRT1/ PTR FAMILY 6.4 (AHRD V3.3 *** A0A2G2X304_CAPBA)
Solyc04g150161.1	-2.258879331	5.27433173	0.00026719	0.02887112	Unknown protein
Solyc01g101180.4	-4.340067897	0.07603704	0.00027004	0.02893158	Terpene synthase (AHRD V3.3 *** G5CV44_SOLLC)
Solyc04g071990.3	1.096596416	7.0372545	0.00026998	0.02893158	Protein GIGANTEA (AHRD V3.3 *** A0A2G2ZPW3_CAPAN)

Solyc04g071580.3	1.047840866	4.11621533	0.00027148	0.0289639	Abscicic acid stress ripening 2
Solyc01g100200.3	-1.156640631	5.95848397	0.00027991	0.02950373	gras4
Solyc09g008010.3	1.177117371	6.0896953	0.00028004	0.02950373	Protein kinase (AHRD V3.3 *** Q9M7J5_ELYEL)
Solyc02g078320.3	1.292806967	3.39706006	0.000279	0.02950373	E3 ubiquitin-protein ligase RMA1H1-like (AHRD V3.3 *** A0A2I4ELD1_9ROSI)
Solyc03g083900.4	1.228018224	5.78884425	0.00028554	0.02995808	monocopper oxidase-like protein SKU5 (AHRD V3.3 *** A0A2I4GW38_9ROSI)
Solyc04g076610.3	1.50948692	2.9157032	0.00029365	0.03055483	Ribonuclease H (AHRD V3.3 *** A0A2K3N9F8_TRIPR)
Solyc03g111170.3	1.686339232	5.00428694	0.00029357	0.03055483	4-coumarate-CoA ligase (AHRD V3.3 *** E5GBV5_CUCME)
Solyc06g061070.3	-0.972525348	4.52297824	0.00029914	0.03099853	Glycine cleavage system H protein (AHRD V3.3 *** A0A1U8GUW6_CAPAN)
Solyc02g090210.3	-2.963695195	0.33880078	0.00030506	0.03148346	GDSL esterase/lipase (AHRD V3.3 *** A0A2G3D8U2_CAPCH)
Solyc09g059170.3	-2.721420411	-0.3795648	0.00031013	0.03183574	Glycosyltransferase (AHRD V3.3 *** A4GRT2_9SOLA)
Solyc04g051310.4	1.13790571	5.89636879	0.00031099	0.03183574	transmembrane protein (AHRD V3.3 *** AT5G16520.1)
Solyc10g085020.3	1.024961845	6.07262499	0.00032396	0.03302912	Tubulin beta chain (AHRD V3.3 *** A0A1U8EHY4_CAPAN)
Solyc09g089730.3	-1.26459863	3.60615647	0.0003287	0.03324447	2-oxoglutarate (2OG) and Fe(II)-dependent oxygenase superfamily protein (AHRD V3.3 *** A0A2U1NTU8_ARTAN)
Solyc12g009180.2	1.085697558	3.94483222	0.00032765	0.03324447	Cupin_5 domain-containing protein (AHRD V3.3 *** A0A1Q3BA35_CEPFO)
Solyc03g031700.4	-1.012998976	6.57095989	0.00033333	0.03344535	Fe-S cluster assembly protein SufB (AHRD V3.3 *** A0A1P8DZ57_9SPHI)
Solyc03g119520.4	0.963786711	5.75592359	0.00033289	0.03344535	BnaC05g48200D protein (AHRD V3.3 *** A0A078HZC2_BRANA)
Solyc00g500041.1	-6.675724683	0.0465918	0.0003376	0.03373996	NAD(P)H-quinone oxidoreductase subunit 1, chloroplastic (AHRD V3.3 *** A0A2G2XBN0_CAPBA)
Solyc11g068940.1	3.684666969	1.14142215	0.00034248	0.03409342	RING-type E3 ubiquitin transferase (AHRD V3.3 *** A0A2G2VPS7_CAPBA)
Solyc01g111260.2	0.90327817	6.68890903	0.00034466	0.03417513	1-phosphatidylinositol phosphodiesterase (AHRD V3.3 *** A0A1J3JKG9_NOCCA)
Solyc10g083940.1	-1.060274952	5.83448185	0.00035094	0.03466183	Major facilitator superfamily (AHRD V3.3 *** A0A2U1KPS1_ARTAN)
Solyc01g102290.3	0.970889398	8.15460894	0.00035337	0.03476613	Low temperature and salt responsive protein (AHRD V3.3 *** Q8H9B8_SOLTU)
Solyc11g011170.2	-1.116898967	6.33778604	0.00035993	0.03527464	Senescence-associated family protein (AHRD V3.3 *** A0A2U1QIG8_ARTAN)
Solyc12g006240.2	1.793300155	2.98527039	0.00036344	0.03548062	Zinc finger protein CONSTANS-LIKE 9 (AHRD V3.3 *** A0A2G2YTW9_CAPAN)
Solyc03g116700.4	2.771759743	2.87351143	0.00037012	0.03599434	Umecyanin (AHRD V3.3 *** A0A2G3B789_CAPCH)
Solyc07g009500.3	-2.115864119	6.46573051	0.00038424	0.03722436	Chitinase 12 (AHRD V3.3 *** A0A2G2VG15_CAPBA)
Solyc06g082940.3	-1.910323909	1.0436921	0.00039141	0.03729605	Photosystem I reaction center subunit XI protein (AHRD V3.3 *** A0A2U1MVK5_ARTAN)
Solyc01g080460.3	-1.27661234	10.8519534	0.0003865	0.03729605	Pyruvate, phosphate dikinase (AHRD V3.3 *** A0A0M8KRU1_NICAT)
Solyc07g042500.4	-1.000893726	7.27138354	0.00039148	0.03729605	alpha/beta-Hydrolases superfamily protein (AHRD V3.3 *** AT2G03140.9)
Solyc10g024410.2	0.849050153	6.52321647	0.00038877	0.03729605	Ribose-phosphate pyrophosphokinase (AHRD V3.3 *** A0A0U9HNH5_KLENI)
Solyc10g011660.3	1.353873445	2.7896386	0.00039236	0.03729605	Auxin-responsive GH3 family protein (AHRD V3.3 *** A0A2U1NZC8_ARTAN)
Solyc02g067890.3	1.152549829	3.81631136	0.00039458	0.0373673	S-adenosyl-L-methionine-dependent methyltransferases superfamily protein (AHRD V3.3 *** A0A2U1MED4_ARTAN)
Solyc02g089630.3	1.876831896	5.58788804	0.00039995	0.03773443	Proline dehydrogenase (AHRD V3.3 *** A0A2G2XIU2_CAPBA)
Solyc09g091510.3	-1.649367308	5.01564221	0.00040724	0.03827887	chalcone synthase 1
Solyc12g150132.1	-2.816003691	0.80513109	0.00041835	0.03917807	extensin-2-like (AHRD V3.3 *-* A0A1U8ESB3_CAPAN)
Solyc12g095800.2	1.067019849	5.11669963	0.00042315	0.03946442	Chaperone protein dnaJ 10 (AHRD V3.3 *** A0A2G2YA74_CAPAN)

Solyc03g119850.2	1.762564782	1.06977047	0.00042453	0.03946442	Protein decapping 5 (AHRD V3.3 *-* A0A2G3B6L4_CAPCH)
Solyc01g098720.3	-5.69363563	-0.6967849	0.0004322	0.03974561	Basic helix-loop-helix (BHLH) DNA-binding superfamily protein (AHRD V3.3 *** A0A2U1PAJ2_ARTAN)
Solyc07g065250.3	1.96203553	2.65535728	0.0004313	0.03974561	MAP kinase kinase kinase 60
Solyc02g081980.3	2.494482945	0.68262352	0.00043227	0.03974561	Apyrase (AHRD V3.3 *** A0A2G2XFE6_CAPBA)
Solyc04g072840.3	-1.063606957	3.996173	0.00043729	0.04006083	P-loop containing nucleoside triphosphate hydrolases superfamily protein (AHRD V3.3 *-* A0A2U1QEJ7_ARTAN)
Solyc09g092670.3	-1.79430157	0.9729319	0.00044372	0.040461	Cytochrome (AHRD V3.3 *** A0A1U8G4E5_CAPAN)
Solyc02g067230.3	-1.493091183	3.12967479	0.00044806	0.040461	Dof zinc finger protein3
Solyc04g079030.2	-1.430147478	2.30323793	0.00044802	0.040461	Glycosyltransferase (AHRD V3.3 *** V4NB36_EUTSA)
Solyc02g088630.4	1.813115314	6.6428616	0.00044619	0.040461	Hexosyltransferase (AHRD V3.3 *** A0A1U8FQ34_CAPAN)
Solyc09g008370.1	-1.646295057	2.80182567	0.00046702	0.04202345	Major facilitator superfamily (AHRD V3.3 *** A0A2U1PF99_ARTAN)
Solyc03g113430.3	1.71201775	3.30254331	0.00047433	0.04252962	Protein NRT1/ PTR FAMILY 2.9 (AHRD V3.3 *** A0A2G2WRK6_CAPBA)
Solyc06g082920.3	1.036443451	6.38119908	0.00049064	0.04383697	Receptor-like kinase (AHRD V3.3 *** D4QD70_DIACA)
Solyc06g072710.3	-1.668304643	1.87209083	0.00049966	0.04448463	RNA polymerase sigma factor sigA (AHRD V3.3 *** A0A2G2ZD00_CAPAN)
Solyc01g006290.4	-1.497864002	4.4033663	0.0005032	0.04448463	Peroxidase (AHRD V3.3 *** K4ASJ5_SOLLC)
Solyc12g013840.3	-1.044494504	5.13203197	0.00050493	0.04448463	Protein SPA1-RELATED 3 (AHRD V3.3 *** A0A2G2VZ51_CAPBA)
Solyc09g091990.3	1.804352386	3.53695121	0.00050462	0.04448463	Receptor like protein kinase S.2 (AHRD V3.3 *** A0A2G2ZTR0_CAPAN)
Solyc12g100240.1	-5.63417958	-0.7323794	0.00051081	0.04453727	Fatty acid desaturase (AHRD V3.3 *-* A0A200QRI1_9MAGN)
Solyc07g061790.3	-1.520117343	5.75776813	0.00051069	0.04453727	Heme-binding protein 2-like (AHRD V3.3 *** A0A2K3NGA7_TRIPR)
Solyc10g083630.2	-0.933125248	9.50597181	0.00050824	0.04453727	DNA demethylase 2
Solyc10g086760.2	1.29734556	3.7888347	0.00051972	0.0451584	Tubulin beta chain (AHRD V3.3 *** A0A1U8EHY4_CAPAN)
Solyc02g077040.4	1.185000958	9.65371951	0.00052853	0.04576632	phytophthora-inhibited protease 1
Solyc03g042560.3	-5.366729897	-0.8895218	0.00053747	0.04638167	Phenylalanine ammonia-lyase (AHRD V3.3 *** A0A2G2YQ27_CAPAN)
Solyc03g096840.4	1.088644749	4.19111785	0.00054839	0.04716316	hypothetical protein (AHRD V3.3 *** AT5G17165.1)
Solyc02g093860.3	2.44725561	3.10025651	0.00055981	0.04798276	Lysine histidine transporter-like 1 (AHRD V3.3 *** A0A1U8FZ64_CAPAN)
Solyc02g020940.3	-3.147322719	-0.3662751	0.00057021	0.04854443	Glyceraldehyde-3-phosphate dehydrogenase (AHRD V3.3 *** A0A2G3D2C2_CAPCH)
Solyc07g007560.3	-1.465954867	4.29912454	0.00057006	0.04854443	Phosphate transporter 45 (AHRD V3.3 *** A0A1P8BAK7_ARATH)
Solyc03g025670.3	-2.128153382	1.93636954	0.00057266	0.04858949	PAR1 (AHRD V3.3 *** A0A2U1P9T7_ARTAN)
Solyc06g075610.1	-1.49971185	1.61485296	0.00057926	0.04882195	Exocyst subunit Exo70 family protein (AHRD V3.3 *** A0A2G2WNK6_CAPBA)
Solyc04g048900.3	1.071192598	4.99596115	0.00057921	0.04882195	Calreticulin (AHRD V3.3 *** A0A2G3ASL1_CAPCH)
Solyc07g061990.3	-1.586799913	7.42184253	0.00058182	0.04887491	Solanesyl diphosphate synthase (AHRD V3.3 *** Q1W5D1_HEVBR)
Solyc10g084690.2	1.597856977	2.45976102	0.00059001	0.04939885	ADP-ribosylation factor GTPase-activating protein 1 (AHRD V3.3 *** A0A1U8EA26_CAPAN)
Solyc12g009020.2	0.842977732	5.29634045	0.00059246	0.04943992	MAP kinase kinase 1
Solyc07g063550.3	0.897179475	7.25269524	0.00059855	0.04971202	Arf GTPase activating protein (AHRD V3.3 *** A0A200RBV9_9MAGN)
Solyc06g082440.1	1.607953895	5.71166712	0.00059965	0.04971202	Non-specific serine/threonine protein kinase (AHRD V3.3 *** G4XMX3_SOLLC)
Solyc08g080290.4	2.886260473	2.96045735	0.00060298	0.04982468	Ethylene-responsive transcription factor (AHRD V3.3 *** A0A2G2XKF3_CAPBA)

* Solyc09g090980.3, showed by the light gray line, was found to be consistent with the tomato allergen Sola l 4.0201 from a blastp search.

Table S4. List of DEGs showing FDR < 0.05 in the comparison of NtLuc/MT and Nt/MT.

**Gene**	**logFC: (NtLuc/MT) / (Nt/MT)**	**logCPM**	**PValue**	**FDR**	**Description**
Solyc00g500332.1	-6.987291035	0.34667273	5.70E-11	9.07E-07	ATP synthase subunit alpha, chloroplastic (AHRD V3.3 *** I6N942_DATST)
Solyc00g500041.1	-7.209623007	0.52291974	7.17E-11	9.07E-07	NAD(P)H-quinone oxidoreductase subunit 1, chloroplastic (AHRD V3.3 *** A0A2G2XBN0_CAPBA)

Table S5. List of genes commonly detected as DEGs in the heterograft lines (Nt/MT and NtLuc/MT) and the homograft line (MT/MT).

Gene	logFC: (Nt/MT) / (MT/MT)	logFC: (NtLuc/MT) / (MT/MT)	Description
Solyc04g064690.4	6.887055598	6.388157804	Peroxidase (AHRD V3.3 *** K4BT60_SOLLC)
Solyc05g009340.1	4.593174142	4.114452204	Unknown protein
Solyc11g005240.1	3.62491992	3.816690699	protein SENSITIVITY TO RED LIGHT REDUCED 1-like (AHRD V3.3 *-* A0A2I4GZX1_9ROSI)
Solyc06g005680.4	3.537268573	4.398197361	Two-component response regulator (AHRD V3.3 *-* C0HE02_MAIZE)
Solyc06g073080.4	3.497150983	3.31217654	2-oxoglutarate (2OG) and Fe(II)-dependent oxygenase superfamily protein (AHRD V3.3 *** A0A2U1QGX7_ARTAN)
Solyc01g095140.4	3.441219592	3.602261635	Desiccation protectant protein Lea14-like protein (AHRD V3.3 *** A0A2G3BT76_CAPCH)
Solyc11g068940.1	3.031906149	3.684666969	RING-type E3 ubiquitin transferase (AHRD V3.3 *** A0A2G2VPS7_CAPBA)
Solyc05g053080.2	2.999280683	2.924704015	Unknown protein
Solyc03g078090.4	2.989891399	4.60495248	Pectinesterase (AHRD V3.3 *** A0A2I4GT13_9ROSI)
Solyc05g053070.4	2.954364549	2.856712345	Unknown protein
Solyc06g068960.1	2.882212711	3.224922199	Calcium-binding allergen Ole e 8 (AHRD V3.3 *** A0A2G2WLM3_CAPBA)
Solyc10g076660.2	2.805824299	2.488434988	2-oxoglutarate (2OG) and Fe(II)-dependent oxygenase superfamily protein (AHRD V3.3 *** F4J670_ARATH)
Solyc04g051360.3	2.772486412	5.449668757	Ethylene Response Factor D.1
Solyc08g078870.3	2.658819194	2.361590192	Bifunctional inhibitor/lipid-transfer protein/seed storage 2S albumin superfamily protein (AHRD V3.3 *** A0A2U1PU47_ARTAN)
Solyc02g085400.3	2.638029447	2.588599577	Sulfite exporter TauE/SafE family protein (AHRD V3.3 *** A0A2U1KY14_ARTAN)
Solyc03g080190.3	2.619460989	3.65187658	2-oxoglutarate (2OG) and Fe(II)-dependent oxygenase superfamily protein (AHRD V3.3 *** A0A2U1QGX7_ARTAN)
Solyc11g072480.2	2.548834916	2.215645885	Tetraspanin-3 (AHRD V3.3 *** A0A2G3BA66_CAPCH)
Solyc04g081960.1	2.49778639	3.5711609	Syringolide-induced protein 14-1-1 (AHRD V3.3 *** A0A2K3M7L2_TRIPR)
Solyc04g078880.3	2.481052799	2.250773279	cold regulated protein 27 (AHRD V3.3 *** AT5G42900.2)
Solyc02g081030.4	2.466362894	2.899339743	ERD (Early-responsive to dehydration stress) family protein (AHRD V3.3 *-* A0A2U1NFB6_ARTAN)
Solyc06g076350.3	2.368642619	2.713800452	LePCL1
Solyc01g100000.3	2.320711851	2.001975169	F-box domain, Phloem protein 2-like protein (AHRD V3.3 *** A0A2U1MNC1_ARTAN)
Solyc02g081980.3	2.304794034	2.494482945	Apyrase (AHRD V3.3 *** A0A2G2XFE6_CAPBA)
Solyc07g063320.3	2.267413186	1.874173171	LanC-like protein GCR2 (AHRD V3.3 *** A0A2G3C294_CAPCH)
Solyc12g087950.1	2.219807102	2.534008638	AT-hook motif nuclear-localized protein (AHRD V3.3 *** A0A1U8GD78_CAPAN)
Solyc04g081650.3	2.194973687	2.101118288	Cyclin (AHRD V3.3 *-* A0A200PQW1_9MAGN)
Solyc12g044230.2	2.179746491	2.007645362	Sulfite exporter TauE/SafE family protein (AHRD V3.3 *** A0A2U1KY14_ARTAN)
Solyc04g082200.2	2.167775147	2.085263418	dehydrin
Solyc02g089630.3	2.135025972	1.876831896	Proline dehydrogenase (AHRD V3.3 *** A0A2G2XIU2_CAPBA)
Solyc01g108100.3	2.117889069	1.370741838	cold regulated protein 27 (AHRD V3.3 *** AT5G42900.2)
Solyc02g077110.3	2.111387038	2.090177881	Phospholipase A1-II 1 (AHRD V3.3 *** A0A2G2XE87_CAPBA)
Solyc02g014860.3	2.080596185	2.323002819	Chaperone protein DnaJ (AHRD V3.3 *** A0A1J3ID64_NOCCA)

Solyc07g064720.3	2.01167152	2.821729535	Gdsl esterase/lipase (AHRD V3.3 *** A0A2P4JQK3_QUESU)
Solyc07g043230.3	1.971143095	2.56273447	Zinc transporter protein (AHRD V3.3 *** A5BDR3_VITVI)
Solyc03g115770.3	1.940465901	1.843346952	Two-component response regulator-like APRR5 (AHRD V3.3 *** A0A2G3CZB4_CAPCH)
Solyc06g075660.4	1.926757271	3.130839261	MYB transcription factor (AHRD V3.3 *-* B4FPU3_MAIZE)
Solyc12g006240.2	1.853288884	1.793300155	Zinc finger protein CONSTANS-LIKE 9 (AHRD V3.3 *** A0A2G2YTW9_CAPAN)
Solyc07g064160.3	1.820074423	1.97900669	Thiamine thiazole synthase, chloroplastic (AHRD V3.3 *** A0A2G2WFN7_CAPBA),Pfam:PF01946
Solyc12g006850.2	1.8137441	1.90007326	LELKT1GEN L.esculentum potassium channel
Solyc08g075370.3	1.709219814	1.766152068	Unknown protein
Solyc07g062700.3	1.691346213	1.933646493	Calcium-binding EF-hand family protein (AHRD V3.3 *** A0A2U1MZJ3_ARTAN)
Solyc10g081170.2	1.681759994	1.777665169	Calmodulin 2
Solyc10g081570.3	1.668629748	1.841454909	Marmande
Solyc06g066800.2	1.65355469	2.477203194	Nucleotide-diphospho-sugar transferases superfamily protein (AHRD V3.3 *** AT1G64980.3)
Solyc10g085420.3	1.640990023	1.941703461	Protein LURP-one-related 15 (AHRD V3.3 *** A0A2G2VU46_CAPBA)
Solyc12g005910.2	1.620942909	1.734652227	B-cell receptor-associated 31-like (AHRD V3.3 *** A0A200QH03_9MAGN)
Solyc09g009530.4	1.60557658	1.437608582	Alpha/beta-Hydrolases superfamily protein (AHRD V3.3 *** Q9SJM9_ARATH)
Solyc04g082140.3	1.591285218	2.177446894	pectinesterase
Solyc09g075300.3	1.587587071	2.002571786	Epoxide hydrolase 2 (AHRD V3.3 *** B6T857_MAIZE)
Solyc01g079610.3	1.519121103	1.449336974	DnaJ protein ERDJ3B (AHRD V3.3 *** A0A2G2Z6I0_CAPAN)
Solyc04g072020.3	1.48968728	1.880571376	Choline/Ethanolamine kinase (AHRD V3.3 *** A0A200QCA0_9MAGN),Pfam:PF01633
Solyc02g089520.2	1.47204129	1.286250869	Zinc finger protein CONSTANS-LIKE 2 (AHRD V3.3 *** A0A2G3ABV8_CAPAN)
Solyc06g009270.3	1.469974216	1.841238505	Diacylglycerol kinase (AHRD V3.3 *** A0A2G3ATC8_CAPCH)
Solyc05g024010.3	1.449029099	1.665940852	Zinc finger protein CONSTANS-LIKE 15 (AHRD V3.3 *** A0A2G2VMU9_CAPBA)
Solyc10g086690.3	1.438356515	1.794235159	Phosphatidylinositol:ceramide inositolphosphotransferase (AHRD V3.3 *-* A0A2G3BFT1_CAPCH)
Solyc03g093510.2	1.416631769	2.205869011	Alpha/beta-Hydrolases superfamily protein (AHRD V3.3 *** A0A2U1NKR4_ARTAN)
Solyc04g081240.2	1.381005697	1.211919759	Auxin Response Factor 5
Solyc06g063060.3	1.314496537	1.710052354	Dormancy/auxin associated protein (AHRD V3.3 *-* A0A2K3JP12_TRIPR)
Solyc09g008280.2	1.310311306	1.657922745	S-adenosyl-L-methionine synthetase Z24743
Solyc07g044980.3	1.288812948	1.130400795	NIM1-like protein 2
Solyc02g092000.3	1.285777328	1.650376421	transmembrane protein (AHRD V3.3 *** AT3G49720.3)
Solyc01g095970.3	1.282575735	1.772579118	Dynamin-like protein (AHRD V3.3 *** F4K015_ARATH)
Solyc03g111170.3	1.280081956	1.686339232	4-coumarate-CoA ligase (AHRD V3.3 *** E5GBV5_CUCME)
Solyc06g036110.1	1.222696457	1.732909066	Calcium-dependent lipid-binding (CaLB domain) family protein (AHRD V3.3 *** A0A2U1LCI8_ARTAN)
Solyc07g061950.4	1.186222402	1.608495622	C2 domain-containing protein (AHRD V3.3 *** A0A2G2WER0_CAPBA)
Solyc07g063520.3	1.176148618	1.716411388	Organic solute transporter ostalpha protein (DUF300) (AHRD V3.3 *** O65422_ARATH)
Solyc02g068430.4	1.175573406	1.345685746	Choline-phosphate cytidylyltransferase (AHRD V3.3 *** A0A2I0V8H3_9ASPA)
Solyc06g068090.3	1.133716838	1.456227789	phospholipase PLDa1
Solyc01g088560.3	1.10719568	1.35320205	Rab family GTPase (AHRD V3.3 *** A0A1Y1IFP9_KLENI)
Solyc09g008010.3	1.10225142	1.177117371	Protein kinase (AHRD V3.3 *** Q9M7J5_ELYEL)

Solyc01g005140.3	1.101754718	1.272996755	Cytochrome b561/ferric reductase transmembrane (AHRD V3.3 *** A0A2U1MNP8_ARTAN)
Solyc12g008940.2	1.094868857	1.108631468	Nucleosome assembly protein family (AHRD V3.3 *** A9TVZ4_PHYPA)
Solyc06g083310.3	1.089228835	1.305001309	Hexosyltransferase (AHRD V3.3 *** A0A1U8H237_CAPAN)
Solyc01g111520.3	1.085897374	1.120358568	Calcium-dependent lipid-binding (CaLB domain) family protein (AHRD V3.3 *** A0A1P8BD17_ARATH)
Solyc01g005300.4	1.080283626	1.047008248	Adagio-like protein 1 (AHRD V3.3 *** A0A1U8H9V5_CAPAN)
Solyc08g075870.3	1.062347626	1.474559442	S-adenosyl-L-methionine-dependent methyltransferases superfamily protein (AHRD V3.3 *** A0A2U1NU97_ARTAN)
Solyc01g095150.3	1.047200643	1.131971872	late embryogenesis-like protein
Solyc01g094370.3	0.992881752	1.331314736	remorin 2
Solyc01g109660.2	0.990063071	1.112491884	meloidogyne-induced giant cell protein DB275
Solyc03g116590.3	0.970360425	1.112443531	Embryo-specific protein (AHRD V3.3 *** A0A2K3LFD9_TRIPR)
Solyc02g077040.4	0.960233496	1.185000958	phytophthora-inhibited protease 1
Solyc03g097270.3	0.948777085	1.000399065	cystatin 9
Solyc03g115370.3	0.930534942	1.183049452	Diacylglycerol kinase (AHRD V3.3 *** A0A1U8G5K6_CAPAN)
Solyc10g085230.2	-0.993072038	-1.095554051	ripening-related mRNA 1b
Solyc07g042500.4	-0.997057654	-1.000893726	alpha/beta-Hydrolases superfamily protein (AHRD V3.3 *** AT2G03140.9)
Solyc08g075210.2	-1.174354452	-1.11882076	HXXXD-type acyl-transferase family protein (AHRD V3.3 *** F4JBC7_ARATH)
Solyc11g011170.2	-1.175754515	-1.116898967	Senescence-associated family protein (AHRD V3.3 *** A0A2U1QIG8_ARTAN)
Solyc06g084770.2	-1.253110323	-1.482331814	cytochrome P450 CYP72A219 (AHRD V3.3 --* XP_004244272.1)
Solyc01g080460.3	-1.326069767	-1.27661234	Pyruvate, phosphate dikinase (AHRD V3.3 *** A0A0M8KRU1_NICAT)
Solyc04g072033.1	-1.332539866	-1.30172454	NAD(P)-binding Rossmann-fold superfamily protein (AHRD V3.3 *** Q9SZ91_ARATH)
Solyc05g050007.1	-1.336039293	-1.334608886	Serine/threonine-protein phosphatase 7 long form-like protein (AHRD V3.3 *-* A0A1J3IZ43_NOCCA)
Solyc10g083940.1	-1.357983361	-1.060274952	Major facilitator superfamily (AHRD V3.3 *** A0A2U1KPS1_ARTAN)
Solyc10g086180.2	-1.363457835	-1.376297523	Phenylalanine ammonia-lyase (AHRD V3.3 *** A0A2G2VU16_CAPBA)
Solyc06g008920.3	-1.388416361	-1.369045662	AMP-dependent synthetase/ligase (AHRD V3.3 *** A0A200QNF3_9MAGN)
Solyc12g088460.3	-1.467698569	-1.4501743	Cytochrome P450 (AHRD V3.3 *** A0A200Q0W8_9MAGN)
Solyc12g098920.2	-1.491202333	-1.487964201	Guanosine-3'5'-bis(Diphosphate) 3'-pyrophosphohydrolase (AHRD V3.3 *** E5GC47_CUCME)
Solyc06g068230.4	-1.492897069	-1.442691036	Tetratricopeptide repeat (TPR)-like superfamily protein (AHRD V3.3 *** A0A2U1MYN5_ARTAN)
Solyc09g091580.3	-1.516544719	-1.450523283	Protein kinase domain (AHRD V3.3 *** A0A200QUN4_9MAGN)
Solyc02g089350.3	-1.52562391	-1.773866619	Gibberellin regulated protein (AHRD V3.3 *** A0A2U1PE48_ARTAN)
Solyc01g006290.4	-1.551887691	-1.497864002	Peroxidase (AHRD V3.3 *** K4ASJ5_SOLLC)
Solyc01g088090.3	-1.562549128	-1.388373013	Pheophytinase, chloroplastic (AHRD V3.3 *** A0A2G3C5T3_CAPCH)
Solyc06g051400.3	-1.583233345	-1.23992982	omega-3 fatty acid desaturase
Solyc05g050010.3	-1.602227595	-1.509525761	1-aminocyclopropane-1-carboxylic acid synthase-4
Solyc07g061990.3	-1.636342603	-1.586799913	Solanesyl diphosphate synthase (AHRD V3.3 *** Q1W5D1_HEVBR)
Solyc08g066705.1	-1.638878277	-2.142039299	Gag-pol polyprotein (AHRD V3.3 *** E6Y5Q4_SOLLC)
Solyc05g011890.1	-1.66698348	-1.346174777	Sulfotransferase (AHRD V3.3 *** A0A2G3B6Y2_CAPCH)
Solyc10g006860.4	-1.710202066	-1.221755289	NAD(P)-binding Rossmann-fold superfamily protein (AHRD V3.3 *** F4IPI3_ARATH)

Solyc05g007880.4	-1.764137654	-2.213670618	cyclic dof factor 2-like (AHRD V3.3 *** A0A2I4E3P6_9ROSI)
Solyc06g072710.3	-1.786038882	-1.668304643	RNA polymerase sigma factor sigA (AHRD V3.3 *** A0A2G2ZD00_CAPAN)
Solyc04g015750.3	-1.798180501	-1.66567647	CobN/magnesium chelatase (AHRD V3.3 *** A0A200PZ28_9MAGN)
Solyc05g051750.3	-1.893859413	-1.753683933	TOMPRORNA prosystemin
Solyc01g095530.2	-1.938584114	-1.757049111	hypothetical protein (AHRD V3.3 *-* AT5G41761.1)
Solyc09g082690.3	-1.945474414	-2.288903921	superoxide dismutase
Solyc01g105350.3	-2.040868835	-2.281617045	Glycosyltransferase (AHRD V3.3 *** A0A1Q3B719_CEPFO)
Solyc10g076710.3	-2.209255417	-2.515579381	Phosphoinositide phospholipase C (AHRD V3.3 *** O49950_SOLTU)
Solyc10g075150.2	-2.425110336	-2.569027778	Non-specific lipid-transfer protein (AHRD V3.3 *** A0A2G2YKI8_CAPAN)
Solyc01g103650.3	-2.439546689	-2.352990307	Alpha/beta-Hydrolases superfamily protein (AHRD V3.3 *** Q9XID7_ARATH)
Solyc10g085240.1	-2.503104968	-2.93876033	UDP-glycosyltransferase 76E1 (AHRD V3.3 *** U76E1_SOLLC)
Solyc10g017970.1	-2.527732953	-2.748311969	Chitinase (AHRD V3.3 *-* B8QVJ5_ZEAMP)
Solyc01g102610.3	-2.549735014	-2.101275456	Ferric reduction oxidase 6 (AHRD V3.3 *** A0A2G3BUN6_CAPCH)
Solyc10g055800.2	-2.632811945	-3.050067643	Chitinase (AHRD V3.3 *** B9VRK7_CAPAN)
Solyc04g071900.4	-2.644206117	-2.834120182	Peroxidase (AHRD V3.3 *** K4BTH7_SOLLC)
Solyc10g017980.1	-2.694514337	-2.861857374	Chitinase (AHRD V3.3 *-* B8QVJ5_ZEAMP)
Solyc04g071070.2	-2.781125904	-2.861893783	Unknown protein
Solyc08g080650.3	-2.85834168	-3.315060601	PATHOGENESIS RELATED PROTEIN P23
Solyc07g009530.1	-2.948316105	-3.452752141	Chitinase (AHRD V3.3 *-* B8QVH4_ZEAMP)
Solyc10g055810.2	-3.156435203	-3.793829446	chitinase Z15140
Solyc03g025670.3	-3.166825844	-2.128153382	PAR1 (AHRD V3.3 *** A0A2U1P9T7_ARTAN)
Solyc07g009230.3	-3.230690861	-4.055782548	Defensin-like protein 1 (AHRD V3.3 *** A0A2G2ZS54_CAPAN)
Solyc09g065440.4	-3.235963681	-4.491634587	Bifunctional inhibitor/lipid-transfer protein/seed storage 2S albumin superfamily protein (AHRD V3.3 *-* A0A2U1NA97_ARTAN)
Solyc12g056675.1	-3.420612213	-5.447474996	Alpha/beta-hydrolases superfamily protein (AHRD V3.3 *** A0A2U1LX98_ARTAN)
Solyc01g059965.1	-3.512892938	-4.289115413	Glucan endo-1,3-beta-glucosidase B (AHRD V3.3 *** E13B_SOLLC)
Solyc01g106620.2	-3.579418331	-3.909163795	pathogenesis-related protein 1-like (AHRD V3.3 *** A0A2I4HRD5_9ROSI)
* Solyc09g090990.2	-3.62941108	-3.558204531	Major allergen Pru ar 1 (AHRD V3.3 *** Q5GMN2_CAPCH)
Solyc09g097760.3	-3.804166477	-4.341219244	Glycine-rich protein (AHRD V3.3 *-* A0A2G2W2G9_CAPBA)
Solyc10g055820.3	-3.949631627	-4.526587747	Chitinase (AHRD V3.3 *** B9VRK7_CAPAN)
Solyc09g006005.1	-4.041918565	-3.353754435	Pathogenesis-related protein 1 (AHRD V3.3 *** Q75QH2_CAPCH)
Solyc04g074020.2	-4.097794052	-2.757135021	Receptor protein kinase, putative (AHRD V3.3 *** A0A061FG24_THECC)
Solyc06g082240.2	-4.158777591	-3.964155613	Laccase (AHRD V3.3 *** K4CA83_SOLLC)
Solyc09g007010.1	-4.188090266	-3.538152773	Pathogenesis-related protein 1 (AHRD V3.3 *** Q75QH2_CAPCH)
Solyc10g079860.2	-4.207516429	-4.930698679	LEQB L.esculentum TomQ'b beta(1,3)glucanase
Solyc12g044954.1	-4.234844287	-5.691787091	Cytochrome (AHRD V3.3 *** A0A2G2Y725_CAPAN)
Solyc04g040130.1	-4.4960874	-6.472192631	Fatty acid desaturase (AHRD V3.3 *** E7CCD0_CAMMC)
Solyc02g090210.3	-4.535708544	-2.963695195	GDSL esterase/lipase (AHRD V3.3 *** A0A2G3D8U2_CAPCH)

Solyc12g100270.2	-4.558475779	-5.225116611	Fatty acid hydroxylase superfamily (AHRD V3.3 *** A0A2U1L8N4_ARTAN)
Solyc12g049030.1	-4.576052129	-4.029277357	Fatty acid desaturase (AHRD V3.3 *** A0A200QRI1_9MAGN)
Solyc04g083140.2	-4.682749043	-4.297430185	Cytochrome (AHRD V3.3 *** A0A1U8F0H8_CAPAN)
Solyc12g100250.3	-4.785290122	-4.213190821	Fatty acid desaturase (AHRD V3.3 *** A0A200QRI1_9MAGN)
Solyc01g106920.4	-4.822278763	-4.363227726	Nucleobase-ascorbate transporter 8 (AHRD V3.3 *** A0A1U8DZ27_CAPAN)
Solyc05g053610.2	-4.904323904	-4.76470016	Pleiotropic drug resistance protein (AHRD V3.3 *** C8CA13_CUCSA)
Solyc03g020030.3	-5.288879439	-4.534904762	Proteinase inhibitor type-2 (AHRD V3.3 *** A0A2G3A0T9_CAPAN)
Solyc03g019690.1	-5.599793064	-4.634655114	Serine protease inhibitor 1 (AHRD V3.3 *** A0A2G2XWH1_CAPAN)
Solyc12g044950.3	-7.424526392	-5.026833776	lipid desaturase
Solyc01g060020.4	-8.959775487	-8.975471509	beta-1,3-glucanase TOMB13GLUB

* Solyc09g090990.2, showed by the light gray line, was found to be consistent with the tomato allergen Sola l 4.0101 from a blastp search.

Table S6. The gene commonly detected as a DEG in both transgrafts (NtLuc/MT) and non-transgrafts (MT/MT and Nt/MT).

**Gene**	**logFC: (NtLuc/MT) / (MT/MT)**	**logFC: (NtLuc/MT) / (Nt/MT)**	**Description**
Solyc00g500041.1	-6.675724683	-7.209623007	NAD(P)H-quinone oxidoreductase subunit 1, chloroplastic (AHRD V3.3 *** A0A2G2XBN0_CAPBA)

Table S7. The list of differentially abundanced proteins identifed by the comparison of Nt/MT and MT/MT.

Uniprot Accession	Description	Abundance Ratio: (Nt/MT) / (MT/MT)	Abundance Ratio Adj. P-Value: (Nt/MT) / (MT/MT)
A0A3Q7J2K1	Acylaminoacyl-peptidase OS=Solanum lycopersicum OX=4081 PE=3 SV=1	0.01	5.00126E-16
A0A494GA05	Uncharacterized protein OS=Solanum lycopersicum OX=4081 PE=3 SV=1	0.102	5.00126E-16
I3QHF0	Proteinase inhibitor II OS=Solanum lycopersicum OX=4081 PE=3 SV=1	0.146	5.00126E-16
A0A3Q7FEG3	Uncharacterized protein OS=Solanum lycopersicum OX=4081 GN=104646135 PE=3 SV=1	0.163	5.00126E-16
A0A3Q7I2A5	Uncharacterized protein OS=Solanum lycopersicum OX=4081 PE=3 SV=1	5.694	9.67081E-14
A0A1S3YUS5	aconitate hydratase, cytoplasmic-like isoform X1 OS=Nicotiana tabacum OX=4097 GN=LOC107779772 PE=3 SV=1	5.979	3.29957E-09
B2LW68	PR1 protein OS=Solanum lycopersicum OX=4081 PE=2 SV=1	0.336	5.54885E-09
K4D5L3	Uncharacterized protein OS=Solanum lycopersicum OX=4081 GN=101244731 PE=4 SV=1	8.655	2.95154E-08
A0A3Q7FSI4	Uncharacterized protein OS=Solanum lycopersicum OX=4081 PE=4 SV=1	0.304	3.9395E-08
A0A3Q7G5J4	AAI domain-containing protein OS=Solanum lycopersicum OX=4081 PE=4 SV=1	0.312	9.20569E-08
Q8GZP5	Allene oxide synthase 3 OS=Solanum lycopersicum OX=4081 GN=AOS3 PE=1 SV=1	3.422	1.4555E-07
Q9LEG1	Cathepsin D Inhibitor OS=Solanum lycopersicum OX=4081 GN=cathDInh PE=3 SV=1	0.321	2.53728E-07
K4BEV4	Tonoplast intrinsic protein 32 OS=Solanum lycopersicum OX=4081 GN=TIP3;2 PE=2 SV=1	0.399	2.44629E-06
A0A3Q7JYB7	AT-hook motif nuclear-localized protein OS=Solanum lycopersicum OX=4081 GN=101247708 PE=4 SV=1	7.501	2.50088E-06
A0A3Q7EII7	Non-specific lipid-transfer protein OS=Solanum lycopersicum OX=4081 GN=101265675 PE=3 SV=1	0.328	3.67257E-06
A0A3Q7I909	Uncharacterized protein OS=Solanum lycopersicum OX=4081 PE=4 SV=1	3.301	2.01942E-05
A0A3Q7FP33	Uncharacterized protein OS=Solanum lycopersicum OX=4081 PE=3 SV=1	0.407	2.2257E-05
A0A3Q7HR13	Phosphoribulokinase OS=Solanum lycopersicum OX=4081 GN=101255322 PE=3 SV=1	0.442	7.019E-05
A0A3Q7H035	Fe2OG dioxygenase domain-containing protein OS=Solanum lycopersicum OX=4081 GN=101244962 PE=3 SV=1	3.004	0.000147455
A0A1S3XQU9	Pectin acetylesterase OS=Nicotiana tabacum OX=4097 GN=LOC107767541 PE=3 SV=1	0.436	0.000399167
A0A1S3X2M1	Oleosin OS=Nicotiana tabacum OX=4097 GN=LOC107760666 PE=3 SV=1	0.449	0.000487249
A0A3Q7HC39	Protein kinase domain-containing protein OS=Solanum lycopersicum OX=4081 GN=101265560 PE=4 SV=1	5.285	0.000493231
A0A3Q7GFL6	SHSP domain-containing protein OS=Solanum lycopersicum OX=4081 PE=3 SV=1	2.834	0.000562836
O81536	Annexin OS=Solanum lycopersicum OX=4081 GN=AN34 PE=2 SV=1	0.447	0.000648799
A0A3Q7HHI5	Fe2OG dioxygenase domain-containing protein OS=Solanum lycopersicum OX=4081 PE=3 SV=1	3.228	0.001034262
A0A3Q7I868	Uncharacterized protein OS=Solanum lycopersicum OX=4081 GN=101247557 PE=3 SV=1	0.467	0.001505225
A0A3Q7J3H0	Uncharacterized protein OS=Solanum lycopersicum OX=4081 GN=101267061 PE=4 SV=1	0.449	0.001927132
A0A3Q7HQ32	Uncharacterized protein OS=Solanum lycopersicum OX=4081 GN=101245370 PE=3 SV=1	0.403	0.002051324
A0A3Q7EGN8	Uncharacterized protein OS=Solanum lycopersicum OX=4081 GN=101263243 PE=3 SV=1	0.449	0.002158847
A0A3Q7HJU0	Pectin acetylesterase OS=Solanum lycopersicum OX=4081 PE=3 SV=1	0.48	0.002484853

A0A3Q7GB69	Glyceraldehyde-3-phosphate dehydrogenase OS=Solanum lycopersicum OX=4081 GN=101264004 PE=3 SV=1	0.488	0.002715509
Q40129	Uncharacterized protein OS=Solanum lycopersicum OX=4081 GN=544001 PE=2 SV=1	0.49	0.003675728
Q9LS37	Small heat shock protein OS=Solanum lycopersicum OX=4081 GN=leer-sHSP PE=2 SV=1	2.28	0.006691567
O49877	CYP1 OS=Solanum lycopersicum OX=4081 GN=C14 PE=2 SV=1	2.115	0.011551026
A0A3Q7FR32	Uncharacterized protein OS=Solanum lycopersicum OX=4081 PE=3 SV=1	2.02	0.012407758
A0A3Q7GIW3	YTH domain-containing protein OS=Solanum lycopersicum OX=4081 GN=101248578 PE=4 SV=1	5.074	0.012806206
Q9XEX8	Remorin 1 OS=Solanum lycopersicum OX=4081 GN=rem-1 PE=2 SV=1	2.026	0.013264009
A0A1S4DPZ2	Proteasome subunit alpha type OS=Nicotiana tabacum OX=4097 GN=LOC107832196 PE=3 SV=1	0.48	0.022251399
A0A3Q7IQV1	HMA domain-containing protein OS=Solanum lycopersicum OX=4081 GN=101247578 PE=4 SV=1	2.106	0.022292643
A0A3Q7H040	Uncharacterized protein OS=Solanum lycopersicum OX=4081 PE=4 SV=1	2.512	0.02245043
Q672Q3	Wound/stress protein OS=Solanum lycopersicum OX=4081 GN=543932 PE=2 SV=1	0.463	0.024091235
A0A3Q7EHY8	DUF3700 domain-containing protein OS=Solanum lycopersicum OX=4081 GN=101253225 PE=4 SV=1	2.049	0.024739343
A0A3Q7J109	Uncharacterized protein OS=Solanum lycopersicum OX=4081 PE=3 SV=1	2.471	0.026225576
A0A3Q7E9H4	Ribosomal protein OS=Solanum lycopersicum OX=4081 PE=3 SV=1	2.026	0.027115421
A0A3Q7FF20	Uncharacterized protein OS=Solanum lycopersicum OX=4081 GN=101264892 PE=4 SV=1	2.249	0.029064241
A0A3Q7H3G0	Histone domain-containing protein OS=Solanum lycopersicum OX=4081 GN=101244086 PE=3 SV=1	0.494	0.030031805
A0A1S4AL04	ferredoxin--nitrite reductase, chloroplastic-like isoform X1 OS=Nicotiana tabacum OX=4097 GN=LOC107798816 PE=3 SV=1	0.403	0.031133022
A0A3Q7J2P9	Uncharacterized protein OS=Solanum lycopersicum OX=4081 GN=101246961 PE=3 SV=1	0.498	0.038341054

Table S8. The list of differentially abundanced proteins identified from the comparison of NtLuc/MT and MT/MT.

**Uniprot Accession**	**Description**	**Abundance Ratio: (NtLuc/MT) / (MT/MT)**	**Abundance Ratio Adj. P-Value: (NtLuc/MT) / (MT/MT)**
A0A3Q7IQ51	Carboxypeptidase OS=Solanum lycopersicum OX=4081 PE=3 SV=1	0.01	4.70335E-16
A0A3Q7H2H9	Uncharacterized protein OS=Solanum lycopersicum OX=4081 PE=3 SV=1	0.01	4.70335E-16
A0A494GA05	Uncharacterized protein OS=Solanum lycopersicum OX=4081 PE=3 SV=1	0.078	4.70335E-16
A0A3Q7FEG3	Uncharacterized protein OS=Solanum lycopersicum OX=4081 GN=104646135 PE=3 SV=1	0.229	5.9622E-13
A0A3Q7H2V7	Uncharacterized protein OS=Solanum lycopersicum OX=4081 PE=4 SV=1	0.215	3.10203E-11
A0A3Q7GVD6	Peroxidase OS=Solanum lycopersicum OX=4081 GN=101261825 PE=3 SV=1	0.25	1.73992E-09
I3QHF0	Proteinase inhibitor II OS=Solanum lycopersicum OX=4081 PE=3 SV=1	0.297	5.32602E-09
A0A3Q7G5J4	AAI domain-containing protein OS=Solanum lycopersicum OX=4081 PE=4 SV=1	0.299	5.82182E-09
A0A3Q7JDI8	PMEI domain-containing protein OS=Solanum lycopersicum OX=4081 GN=101268665 PE=4 SV=1	0.241	8.96208E-09
Q9LEG1	Cathepsin D Inhibitor OS=Solanum lycopersicum OX=4081 GN=cathDInh PE=3 SV=1	0.297	3.02712E-08
A0A1S4CDL2	protein CutA, chloroplastic-like OS=Nicotiana tabacum OX=4097 GN=LOC107817862 PE=3 SV=1	3.732	4.07212E-07
A0A3Q7FSI4	Uncharacterized protein OS=Solanum lycopersicum OX=4081 PE=4 SV=1	0.355	9.04272E-07
A0A3Q7IKF2	Uncharacterized protein OS=Solanum lycopersicum OX=4081 GN=544092 PE=3 SV=1	0.315	9.8764E-07
A0A3Q7IP06	Phosphopyruvate hydratase OS=Solanum lycopersicum OX=4081 PE=3 SV=1	0.3	1.91051E-06
A0A3Q7EII7	Non-specific lipid-transfer protein OS=Solanum lycopersicum OX=4081 GN=101265675 PE=3 SV=1	0.324	3.29781E-06
K4D1U9	Non-specific lipid-transfer protein OS=Solanum lycopersicum OX=4081 GN=101266080 PE=2 SV=1	3.242	5.45428E-06
B2LW68	PR1 protein OS=Solanum lycopersicum OX=4081 PE=2 SV=1	0.428	6.88998E-05
A0A3Q7FP33	Uncharacterized protein OS=Solanum lycopersicum OX=4081 PE=3 SV=1	0.424	7.46919E-05
A0A3Q7G5R7	PLAT domain-containing protein OS=Solanum lycopersicum OX=4081 GN=101262509 PE=4 SV=1	0.368	0.000101159
A0A3Q7G5V9	KOW domain-containing protein OS=Solanum lycopersicum OX=4081 GN=101267290 PE=3 SV=1	4.396	0.000119204
A0A3Q7INV1	Uncharacterized protein OS=Solanum lycopersicum OX=4081 PE=3 SV=1	0.386	0.000171464
A0A3Q7IIQ3	Chitin-binding type-1 domain-containing protein OS=Solanum lycopersicum OX=4081 PE=3 SV=1	0.407	0.000198913
A0A3Q7H521	Uncharacterized protein OS=Solanum lycopersicum OX=4081 GN=101252788 PE=4 SV=1	4.447	0.000267691
A0A1S3XJ54	NADH dehydrogenase [ubiquinone] 1 beta subcomplex subunit 10-A-like OS=Nicotiana tabacum OX=4097 GN=LOC107765821 PE=3 SV=1	0.391	0.000377497
A0A3Q7J2K1	Acylaminoacyl-peptidase OS=Solanum lycopersicum OX=4081 PE=3 SV=1	0.305	0.000383407
A0A3Q7G3K3	Gal_mutarotas_2 domain-containing protein OS=Solanum lycopersicum OX=4081 GN=543798 PE=3 SV=1	4.751	0.000504612
A0A1S3X2M1	Oleosin OS=Nicotiana tabacum OX=4097 GN=LOC107760666 PE=3 SV=1	0.488	0.000598931
A0A494GA45	SCP domain-containing protein OS=Solanum lycopersicum OX=4081 PE=4 SV=1	0.477	0.000913313
Q05538	Basic 30 kDa endochitinase OS=Solanum lycopersicum OX=4081 GN=CHI9 PE=1 SV=1	0.483	0.001178177
A0A3Q7GEE9	Glutamine--tRNA ligase OS=Solanum lycopersicum OX=4081 GN=101251979 PE=3 SV=1	3.73	0.00120631
A0A3Q7G893	RRM domain-containing protein OS=Solanum lycopersicum OX=4081 PE=4 SV=1	0.394	0.001848954
A0A3Q7F7C4	Peroxidase OS=Solanum lycopersicum OX=4081 PE=3 SV=1	0.364	0.002367769

A0A3Q7FMI6	Uncharacterized protein OS=Solanum lycopersicum OX=4081 GN=101250872 PE=4 SV=1	3.1	0.003381727
Q2QJT5	ASR4 OS=Solanum lycopersicum OX=4081 GN=Asr4 PE=2 SV=1	0.484	0.004377556
A0A3Q7JYB7	AT-hook motif nuclear-localized protein OS=Solanum lycopersicum OX=4081 GN=101247708 PE=4 SV=1	0.247	0.004788876
A0A3Q7HHI5	Fe2OG dioxygenase domain-containing protein OS=Solanum lycopersicum OX=4081 PE=3 SV=1	3.851	0.004880339
A0A3Q7IJL2	AAI domain-containing protein OS=Solanum lycopersicum OX=4081 PE=3 SV=1	0.486	0.00519641
A0A3Q7H035	Fe2OG dioxygenase domain-containing protein OS=Solanum lycopersicum OX=4081 GN=101244962 PE=3 SV=1	2.681	0.005286095
A0A3Q7IYJ2	Uncharacterized protein OS=Solanum lycopersicum OX=4081 GN=101267377 PE=4 SV=1	0.468	0.005354713
A0A3Q7EST9	Carboxypeptidase OS=Solanum lycopersicum OX=4081 PE=3 SV=1	0.259	0.007088981
A0A3Q7I593	Uncharacterized protein OS=Solanum lycopersicum OX=4081 GN=101266666 PE=3 SV=1	0.474	0.007571421
A0A3Q7IQV1	HMA domain-containing protein OS=Solanum lycopersicum OX=4081 GN=101247578 PE=4 SV=1	2.457	0.008645778
A0A3Q7G505	PLAT domain-containing protein OS=Solanum lycopersicum OX=4081 GN=101262805 PE=4 SV=1	0.497	0.009869626
A0A3Q7H093	Uncharacterized protein OS=Solanum lycopersicum OX=4081 PE=4 SV=1	2.903	0.020035697
A0A3Q7FV18	Uncharacterized protein OS=Solanum lycopersicum OX=4081 GN=101245630 PE=4 SV=1	3.2	0.020344301
A0A1S4DPZ2	Proteasome subunit alpha type OS=Nicotiana tabacum OX=4097 GN=LOC107832196 PE=3 SV=1	0.491	0.021454939
A0A3Q7JH77	Uncharacterized protein OS=Solanum lycopersicum OX=4081 GN=101265978 PE=4 SV=1	2.716	0.026777571
G3K2M4	Peptide-methionine (S)-S-oxide reductase OS=Solanum lycopersicum OX=4081 GN=MSRA4 PE=3 SV=1	2.094	0.029510391
A0A3Q7FSL1	Uncharacterized protein OS=Solanum lycopersicum OX=4081 PE=4 SV=1	2.785	0.033313029
A0A3Q7G5D7	Uncharacterized protein OS=Solanum lycopersicum OX=4081 PE=3 SV=1	2.714	0.033527905
A0A3Q7HKZ6	Uncharacterized protein OS=Solanum lycopersicum OX=4081 GN=543663 PE=3 SV=1	2.246	0.033977051
A0A1S4D172	protein FAM63B-like OS=Nicotiana tabacum OX=4097 GN=LOC107824775 PE=4 SV=1	2.69	0.037795213
A0A1S4BVP1	Elongation factor 1-alpha OS=Nicotiana tabacum OX=4097 GN=LOC107812355 PE=3 SV=1	2.726	0.037897384

Table S9. The list of differentially abundanced proteins identified by the comparison of NtLuc/MT and Nt/MT.

Uniprot Accession	Description	Abundance Ratio: (NtLuc/MT) / (Nt/MT)	Abundance Ratio Adj. P-Value: (NtLuc/MT) / (Nt/MT)
A0A3Q7H3Y0	Knot1 domain-containing protein OS=Solanum lycopersicum OX=4081 GN=101263826 PE=4 SV=1	0.313	1.19457E-11
A0A3Q7HS54	PAP_fibrillin domain-containing protein OS=Solanum lycopersicum OX=4081 PE=4 SV=1	0.104	1.2122E-09
A0A3Q7F0I8	Uncharacterized protein OS=Solanum lycopersicum OX=4081 GN=104645946 PE=4 SV=1	0.085	4.01011E-09
A0A3Q7FH23	Uncharacterized protein OS=Solanum lycopersicum OX=4081 GN=109119847 PE=3 SV=1	0.171	7.27687E-08
A0A3Q7IJL2	AAI domain-containing protein OS=Solanum lycopersicum OX=4081 PE=3 SV=1	0.359	8.6284E-08
A0A3Q7IKF2	Uncharacterized protein OS=Solanum lycopersicum OX=4081 GN=544092 PE=3 SV=1	0.371	2.59935E-07
K4D1U9	Non-specific lipid-transfer protein OS=Solanum lycopersicum OX=4081 GN=101266080 PE=2 SV=1	2.628	1.14915E-06
A0A3Q7IKC4	Cytochrome b-c1 complex subunit 7 OS=Solanum lycopersicum OX=4081 GN=101262441 PE=3 SV=1	2.335	1.08942E-05
A0A3Q7EEZ8	Uncharacterized protein OS=Solanum lycopersicum OX=4081 PE=3 SV=1	0.474	1.20449E-05
A0A1S4DI50	Lipoxygenase OS=Nicotiana tabacum OX=4097 GN=LOC107830099 PE=3 SV=1	0.382	2.09413E-05
A0A3Q7GEA3	Uncharacterized protein OS=Solanum lycopersicum OX=4081 GN=101254186 PE=4 SV=1	3.131	3.84793E-05
A0A3Q7GFL6	SHSP domain-containing protein OS=Solanum lycopersicum OX=4081 PE=3 SV=1	0.449	5.39363E-05
A0A3Q7FGU5	Uncharacterized protein OS=Solanum lycopersicum OX=4081 GN=101248647 PE=3 SV=1	0.451	0.000111409
A0A3Q7IZK1	Metallophos domain-containing protein OS=Solanum lycopersicum OX=4081 GN=101259706 PE=4 SV=1	2.207	0.000231831
A0A3Q7I801	Bet_v_1 domain-containing protein OS=Solanum lycopersicum OX=4081 GN=101246666 PE=3 SV=1	0.433	0.000443933
Q2MIK1	ATP synthase subunit b, chloroplastic OS=Solanum bulbocastanum OX=147425 GN=atpF PE=3 SV=1	2.329	0.000511959
A0A3Q7IAF8	Uncharacterized protein OS=Solanum lycopersicum OX=4081 PE=4 SV=1	2.779	0.000565728
A0A3Q7IUI4	Knot1 domain-containing protein OS=Solanum lycopersicum OX=4081 GN=101249140 PE=4 SV=1	2.978	0.000639662
A0A1S4AAC1	40S ribosomal protein S29-like OS=Nicotiana tabacum OX=4097 GN=LOC107795411 PE=3 SV=1	0.363	0.000820498
A0A3Q7IIS3	Uncharacterized protein OS=Solanum lycopersicum OX=4081 PE=3 SV=1	2.086	0.000910819
A0A1S4CL01	cell division cycle protein 48 homolog OS=Nicotiana tabacum OX=4097 GN=LOC107820024 PE=4 SV=1	2.074	0.001498396
A0A3Q7HST6	Uncharacterized protein OS=Solanum lycopersicum OX=4081 PE=3 SV=1	0.349	0.002025848
A0A3Q7JGX3	Uncharacterized protein OS=Solanum lycopersicum OX=4081 GN=101252541 PE=4 SV=1	0.343	0.002652514
A0A3Q7ES75	Uncharacterized protein OS=Solanum lycopersicum OX=4081 GN=101244107 PE=3 SV=1	2.109	0.00286081
A0A3Q7FVL0	Uncharacterized protein OS=Solanum lycopersicum OX=4081 PE=3 SV=1	2.421	0.004370662
A0A3Q7FAZ5	Uncharacterized protein OS=Solanum lycopersicum OX=4081 GN=101249974 PE=4 SV=1	2.331	0.005704787
A0A3Q7EQZ3	AAI domain-containing protein OS=Solanum lycopersicum OX=4081 GN=101250539 PE=4 SV=1	2.269	0.005857824
A0A3Q7EQS7	Pectin acetylesterase OS=Solanum lycopersicum OX=4081 GN=101268518 PE=3 SV=1	0.493	0.006349048
A0A3Q7H2V7	Uncharacterized protein OS=Solanum lycopersicum OX=4081 PE=4 SV=1	0.423	0.006479036
A0A3Q7HKZ6	Uncharacterized protein OS=Solanum lycopersicum OX=4081 GN=543663 PE=3 SV=1	2.058	0.009381698
A0A1S3WZE1	Peroxidase OS=Nicotiana tabacum OX=4097 GN=LOC107759554 PE=3 SV=1	0.336	0.009922993
A0A3Q7HC39	Protein kinase domain-containing protein OS=Solanum lycopersicum OX=4081 GN=101265560 PE=4 SV=1	0.333	0.010073042
A0A3Q7FZS6	Uncharacterized protein OS=Solanum lycopersicum OX=4081 PE=4 SV=1	2.063	0.01120885

A0A1S4B6Y6	Sm-like protein LSM2 OS=Nicotiana tabacum OX=4097 GN=LOC107805127 PE=3 SV=1	2.019	0.015845837
A0A3Q7HA91	Uncharacterized protein OS=Solanum lycopersicum OX=4081 PE=4 SV=1	0.387	0.016078395
A0A1S4AWV6	polygalacturonase At1g48100-like OS=Nicotiana tabacum OX=4097 GN=LOC107802165 PE=3 SV=1	2.069	0.016329131
A0A1S4AQ07	30S ribosomal protein S1, chloroplastic-like OS=Nicotiana tabacum OX=4097 GN=LOC107800017 PE=4 SV=1	2.113	0.023452326
A0A1S4CW49	Methyltransferase OS=Nicotiana tabacum OX=4097 GN=LOC107823235 PE=3 SV=1	0.376	0.035084343
A0A3Q7ID82	2,4-dienoyl-CoA reductase ((3E)-enoyl-CoA-producing) OS=Solanum lycopersicum OX=4081 PE=4 SV=1	0.436	0.039416675
A0A3Q7FV18	Uncharacterized protein OS=Solanum lycopersicum OX=4081 GN=101245630 PE=4 SV=1	2.332	0.04204019

Table S10. List of proteins commonly detected as differentially abundanced that were identified from the comparison of heterograft lines (Nt/MT and NtLuc/MT) and homograft line (MT/MT).

Uniprot Accession	Description	Abundance Ratio: (Nt/MT) / (MT/MT)	Abundance Ratio: (NtLuc/MT) / (MT/MT)
A0A3Q7JYB7	AT-hook motif nuclear-localized protein OS=Solanum lycopersicum OX=4081 GN=101247708 PE=4 SV=1	7.501	0.247
A0A3Q7H035	Fe2OG dioxygenase domain-containing protein OS=Solanum lycopersicum OX=4081 GN=101244962 PE=3 SV=1	3.004	2.681
A0A3Q7HHI5	Fe2OG dioxygenase domain-containing protein OS=Solanum lycopersicum OX=4081 PE=3 SV=1	3.228	3.851
A0A3Q7IQV1	HMA domain-containing protein OS=Solanum lycopersicum OX=4081 GN=101247578 PE=4 SV=1	2.106	2.457
A0A1S4DPZ2	Proteasome subunit alpha type OS=Nicotiana tabacum OX=4097 GN=LOC107832196 PE=3 SV=1	0.48	0.491
A0A1S3X2M1	Oleosin OS=Nicotiana tabacum OX=4097 GN=LOC107760666 PE=3 SV=1	0.449	0.488
A0A3Q7FSI4	Uncharacterized protein OS=Solanum lycopersicum OX=4081 PE=4 SV=1	0.304	0.355
A0A3Q7G5J4	AAI domain-containing protein OS=Solanum lycopersicum OX=4081 PE=4 SV=1	0.312	0.299
A0A3Q7EII7	Non-specific lipid-transfer protein OS=Solanum lycopersicum OX=4081 GN=101265675 PE=3 SV=1	0.328	0.324
Q9LEG1	Cathepsin D Inhibitor OS=Solanum lycopersicum OX=4081 GN=cathDInh PE=3 SV=1	0.321	0.297
A0A3Q7FP33	Uncharacterized protein OS=Solanum lycopersicum OX=4081 PE=3 SV=1	0.407	0.424
A0A494GA05	Uncharacterized protein OS=Solanum lycopersicum OX=4081 PE=3 SV=1	0.102	0.078
I3QHF0	Proteinase inhibitor II OS=Solanum lycopersicum OX=4081 PE=3 SV=1	0.146	0.297
B2LW68	PR1 protein OS=Solanum lycopersicum OX=4081 PE=2 SV=1	0.336	0.428
A0A3Q7FEG3	Uncharacterized protein OS=Solanum lycopersicum OX=4081 GN=104646135 PE=3 SV=1	0.163	0.229
A0A3Q7J2K1	Acylaminoacyl-peptidase OS=Solanum lycopersicum OX=4081 PE=3 SV=1	0.01	0.305

Table S11. List of proteins that commonly detected as differently abundanced in the comparison of the non-transgraft lines (Nt/MT and MT/MT) and the hetero-transgraft line (NtLuc/MT).

Uniprot Accession	Description	Abundance Ratio: (NtLuc/MT) / (MT/MT)	Abundance Ratio: (NtLuc/MT) / (Nt/MT)
K4D1U9	Non-specific lipid-transfer protein OS=Solanum lycopersicum OX=4081 GN=101266080 PE=2 SV=1	3.242	2.628
A0A3Q7FV18	Uncharacterized protein OS=Solanum lycopersicum OX=4081 GN=101245630 PE=4 SV=1	3.2	2.332
A0A3Q7HKZ6	Uncharacterized protein OS=Solanum lycopersicum OX=4081 GN=543663 PE=3 SV=1	2.246	2.058
A0A3Q7IJL2	AAI domain-containing protein OS=Solanum lycopersicum OX=4081 PE=3 SV=1	0.486	0.359
A0A3Q7IKF2	Uncharacterized protein OS=Solanum lycopersicum OX=4081 GN=544092 PE=3 SV=1	0.315	0.371
A0A3Q7H2V7	Uncharacterized protein OS=Solanum lycopersicum OX=4081 PE=4 SV=1	0.215	0.423

Table S12. List of proteins detected as differently abundanced in the comparison of Nt/MT to NtLuc/MT and MT/MT.

**Uniprot Accession**	**Description**	**Abundance Ratio: (Nt/MT) / (MT/MT)**	**Abundance Ratio: (NtLuc/MT) / (Nt/MT)**
A0A3Q7HC39	Protein kinase domain-containing protein OS=Solanum lycopersicum OX=4081 GN=101265560 PE=4 SV=1	5.285	0.333
A0A3Q7GFL6	SHSP domain-containing protein OS=Solanum lycopersicum OX=4081 PE=3 SV=1	2.834	0.449

Table S13. List of genes found to have increased or decreased expression levels in common by both transcriptomic and proteomic analyses of the non-transgrafted lines (Nt/MT and MT/MT).

Name	Uniprot Accession	Description	logFC: (Nt/MT) / (MT/MT)	Abundance Ratio: (Nt/MT) / (MT/MT)
Solyc03g020060.3	A0A3Q7FEG3	Proteinase inhibitor type-2 (AHRD V3.3 *** A0A2G2V3L6_CAPBA)	-4.129927638	0.163
Solyc01g106620.2	B2LW68	pathogenesis-related protein 1-like (AHRD V3.3 *** A0A2I4HRD5_9ROSI)	-3.579418331	0.336
Solyc09g097960.3	A0A3Q7I909	NAD(P)-linked oxidoreductase, aldo/keto reductase family protein (AHRD V3.3 *** A0A1Y1HY98_KLENI)	1.630665904	3.301

Table S14. List of genes found to have increased or decreased expression levels in common in both transcriptomic and proteomic analyses of NtLuc/MT and MT/MT.

**Name**	**Uniprot Accession**	**Description**	**logFC: (NtLuc/MT) / (MT/MT)**	**Abundance Ratio: (NtLuc/MT) / (MT/MT)**
Solyc10g079860.2	A0A3Q7IKF2	LEQB L.esculentum TomQ'b beta(1,3)glucanase	-4.930698679	0.315
Solyc03g019690.1	A0A3Q7FGU5	Serine protease inhibitor 1 (AHRD V3.3 *** A0A2G2XWH1_CAPAN)	-4.634655114	0.451
Solyc10g055820.3	A0A3Q7IIQ3	Chitinase (AHRD V3.3 *** B9VRK7_CAPAN)	-4.526587747	0.407
Solyc01g106620.2	B2LW68	pathogenesis-related protein 1-like (AHRD V3.3 *** A0A2I4HRD5_9ROSI)	-3.909163795	0.428
Solyc10g055810.2	Q05538	chitinase Z15140	-3.793829446	0.483
Solyc09g091000.4	A0A3Q7I801	Pathogenesis-related protein STH-2 (AHRD V3.3 *** PRS2_SOLTU)	-3.076837883	0.433
Solyc10g075150.2	A0A3Q7IJL2	Non-specific lipid-transfer protein (AHRD V3.3 *** A0A2G2YKI8_CAPAN)	-2.569027778	0.486
Solyc06g068960.1	A0A3Q7H093	Calcium-binding allergen Ole e 8 (AHRD V3.3 *** A0A2G2WLM3_CAPBA)	3.224922199	2.903

Table S15. The top 10 highest positive and negative loadings for the component 1 from the PCA based on the LC-MS analysis of hydrophilic extracts (corresponds to the score plot graph in Fig. 6).

Ion peak ID	RT[a]	*m/z*[b]	Adduct[c]	Exact mass[d]	Database[e]	Formula[f]	Loadings (PC2)	Mean ion intensity ratio of Nt/MT to MT/MT	Mean ion intensity ratio of NtLuc/MT to MT/MT	Mean ion intensity ratio of NtLuc/MT to Nt/MT
344	3.92	120.081	[M+H]+	119.074	-	Unknown	**0.0332**	8.9	6.3	0.7
337	3.90	163.123	[M+H]+	162.116	UC2	C10H14N2	**0.0331**	9.3	6.6	0.7
2260	10.99	345.116	[M+H]+	344.108	UC2	C22H17N2Cl	**0.0329**	1.4	1.5	1.1
4031	13.44	430.255	[M+H]+	429.248	EX-HR2	C20H35O7N3	**0.0327**	12.0	12.3	1.0
3305	12.49	420.223	[M+H]+	419.216	EX-HR2	C19H33O9N1	**0.0327**	2.1	1.8	0.8
338	3.91	132.081	[M+H]+	131.074	UC2	C9H9N	**0.0326**	9.9	7.0	0.7
4187	13.66	540.244	[M+H]+	539.237	UC2	C23H29O5N11	**0.0320**	2.4	3.1	1.3
340	3.91	117.058	[M+H]+	116.050	EX-HR2	C4H9N2P1	**0.0320**	10.7	7.0	0.7
565	4.85	177.102	[M+H]+	176.095	UC2	C10H12ON2	**0.0316**	11.5	7.8	0.7
1105	8.97	174.058	[M+H]+	173.051	EX-HR2	C7H11O2N1S1	**0.0316**	10.0	11.4	1.1
1455	9.58	203.139	[M+H]+	202.132	UC2	C9H18O3N2	**-0.0371**	0.5	0.6	1.1
4256	13.74	371.207	[M+H]+	370.199	UC2	C19H30O7	**-0.0371**	0.3	0.3	1.0
4593	14.26	231.214	[M+2H]2+	460.414	UC2	C28H52ON4	**-0.0371**	0.3	0.4	1.3
1273	9.24	176.160	[M+2H]2+	350.305	EX-HR2	C20H38O1N4	**-0.0373**	0.5	0.6	1.3
3912	13.21	290.150	[M+H]+	289.143	UC2	C15H19O3N3	**-0.0373**	0.4	0.4	1.1
5831	16.40	547.317	[M+2H]2+	1092.620	EX-HR2	C38H13O1N3P10S8	**-0.0373**	0.5	0.6	1.1
2217	10.94	265.155	[2M+H]+	132.074	-	Unknown	**-0.0375**	0.5	0.4	0.9
1047	8.83	416.119	[M+H]+	415.112	EX-HR2	C10H29O8N3S3	**-0.0376**	0.3	0.3	1.0
4587	14.25	479.432	[M+H]+	478.425	EX-HR2	C21H54N10S1	**-0.0376**	0.3	0.3	1.0
6046	17.17	1092.738	[M+4H]4+	4366.923	-	Unknown	**-0.0377**	0.5	0.6	1.2

[a]Mean retention time (min) of the analyte.

Table S16. List of the 54 ion peaks with statistically significant different abundances among the grafted plant lines by one-way ANOVA.

Ion peak ID	One-way ANOVA			Tuley's HSD	Mean ion intensity ratio of Nt/MT to MT/MT	Mean ion intensity ratio of NtLuc/MT to MT/MT	Mean ion intensity ratio of NtLuc/MT to Nt/MT
	F	p-value	q-value				
71	36.7	7.7E-06	1.4.E-03	Nt/MT-MT/MT; NtLuc/MT-MT/MT	0.4	0.3	0.8
79	70.8	2.3E-07	2.0.E-04	Nt/MT-MT/MT; NtLuc/MT-MT/MT	0.4	0.4	1.0
103	22.5	8.6E-05	6.6.E-03	Nt/MT-MT/MT; NtLuc/MT-MT/MT	0.5	0.5	1.1
109	21.8	1.0E-04	6.9.E-03	Nt/MT-MT/MT; NtLuc/MT-MT/MT	0.4	0.5	1.2
264	30.1	2.1E-05	2.5.E-03	Nt/MT-MT/MT; NtLuc/MT-MT/MT	0.5	0.5	1.0
337	43.8	3.0E-06	1.2.E-03	Nt/MT-MT/MT; NtLuc/MT-MT/MT	9.3	6.6	0.7
338	38.2	6.3E-06	1.3.E-03	Nt/MT-MT/MT; NtLuc/MT-MT/MT	9.9	7.0	0.7
339	36.9	7.5E-06	1.4.E-03	Nt/MT-MT/MT; NtLuc/MT-MT/MT	9.3	6.3	0.7
340	43.2	3.3E-06	1.2.E-03	Nt/MT-MT/MT; NtLuc/MT-MT/MT	10.7	7.0	0.7
344	70.0	2.4E-07	2.0.E-04	Nt/MT-MT/MT; NtLuc/MT-MT/MT; NtLuc/MT-Nt/MT	8.9	6.3	0.7
349	20.6	1.3E-04	7.5.E-03	Nt/MT-MT/MT; NtLuc/MT-MT/MT	0.5	0.4	0.7
433	36.3	8.1E-06	1.4.E-03	Nt/MT-MT/MT; NtLuc/MT-MT/MT	9.3	6.2	0.7
453	26.4	4.0E-05	4.3.E-03	Nt/MT-MT/MT; NtLuc/MT-MT/MT	7.2	5.4	0.8
481	22.0	9.6E-05	6.8.E-03	Nt/MT-MT/MT; NtLuc/MT-MT/MT	0.3	0.2	0.8
498	23.6	7.0E-05	6.2.E-03	Nt/MT-MT/MT; NtLuc/MT-MT/MT	0.5	0.6	1.0
501	21.4	1.1E-04	6.9.E-03	Nt/MT-MT/MT; NtLuc/MT-MT/MT	0.5	0.6	1.0
524	32.8	1.4E-05	1.8.E-03	Nt/MT-MT/MT; NtLuc/MT-MT/MT	14.1	9.2	0.7
565	33.7	1.2E-05	1.8.E-03	Nt/MT-MT/MT; NtLuc/MT-MT/MT	11.5	7.8	0.7
588	19.8	1.6E-04	8.0.E-03	Nt/MT-MT/MT; NtLuc/MT-MT/MT	0.4	0.5	1.1
596	19.7	1.6E-04	8.0.E-03	Nt/MT-MT/MT; NtLuc/MT-MT/MT	0.5	0.6	1.1
597	19.8	1.6E-04	8.0.E-03	Nt/MT-MT/MT; NtLuc/MT-MT/MT	0.5	0.6	1.1
986	40.5	4.6E-06	1.2.E-03	Nt/MT-MT/MT; NtLuc/MT-MT/MT	0.5	0.5	1.0
1047	22.5	8.8E-05	6.6.E-03	Nt/MT-MT/MT; NtLuc/MT-MT/MT	0.3	0.3	1.0
1065	23.2	7.5E-05	6.4.E-03	Nt/MT-MT/MT; NtLuc/MT-MT/MT	0.5	0.5	1.0
1546	33.5	1.2E-05	1.8.E-03	Nt/MT-MT/MT; NtLuc/MT-MT/MT	0.4	0.4	1.1
1787	21.7	1.0E-04	6.9.E-03	Nt/MT-MT/MT; NtLuc/MT-MT/MT	0.3	0.2	0.7
1788	20.0	1.5E-04	8.0.E-03	Nt/MT-MT/MT; NtLuc/MT-MT/MT	0.5	0.4	0.8
1789	23.0	7.9E-05	6.4.E-03	Nt/MT-MT/MT; NtLuc/MT-MT/MT	0.5	0.3	0.7
1794	18.9	1.9E-04	9.0.E-03	Nt/MT-MT/MT; NtLuc/MT-MT/MT	0.6	0.4	0.8
2042	104.4	2.6E-08	6.4.E-05	Nt/MT-MT/MT; NtLuc/MT-MT/MT	0.2	0.2	1.0
2171	40.2	4.8E-06	1.2.E-03	Nt/MT-MT/MT; NtLuc/MT-MT/MT	0.1	0.1	1.0
2709	19.0	1.9E-04	9.0.E-03	NtLuc/MT-MT/MT	1.5	2.0	1.3
2816	25.1	5.2E-05	5.0.E-03	Nt/MT-MT/MT; NtLuc/MT-MT/MT	0.5	0.5	1.0
3287	25.3	5.0E-05	5.0.E-03	Nt/MT-MT/MT; NtLuc/MT-MT/MT	0.5	0.4	0.8
3337	23.8	6.7E-05	6.2.E-03	Nt/MT-MT/MT; NtLuc/MT-MT/MT	0.3	0.4	1.2
3343	18.9	1.9E-04	9.0.E-03	Nt/MT-MT/MT; NtLuc/MT-MT/MT	0.5	0.5	1.0
3520	26.5	4.0E-05	4.3.E-03	Nt/MT-MT/MT; NtLuc/MT-MT/MT	0.4	0.5	1.2
3726	32.1	1.5E-05	1.9.E-03	Nt/MT-MT/MT; NtLuc/MT-MT/MT	0.4	0.4	1.1
3872	22.2	9.3E-05	6.8.E-03	Nt/MT-MT/MT; NtLuc/MT-MT/MT	0.4	0.3	0.7
4131	21.3	1.1E-04	6.9.E-03	Nt/MT-MT/MT; NtLuc/MT-MT/MT	0.7	0.7	1.1
4137	41.1	4.3E-06	1.2.E-03	Nt/MT-MT/MT; NtLuc/MT-MT/MT	0.7	0.8	1.1
4140	39.1	5.5E-06	1.3.E-03	Nt/MT-MT/MT; NtLuc/MT-MT/MT	0.7	0.8	1.1

4239	20.3	1.4E-04	7.9.E-03	Nt/MT-MT/MT; NtLuc/MT-MT/MT	0.3	0.4	1.1
4245	47.3	2.0E-06	1.0.E-03	Nt/MT-MT/MT; NtLuc/MT-MT/MT	0.5	0.5	1.0
4748	23.0	7.8E-05	6.4.E-03	Nt/MT-MT/MT; NtLuc/MT-MT/MT	0.6	0.6	1.0
4749	21.5	1.1E-04	6.9.E-03	Nt/MT-MT/MT; NtLuc/MT-MT/MT	0.6	0.7	1.1
4935	57.3	7.2E-07	4.5.E-04	Nt/MT-MT/MT; NtLuc/MT-MT/MT	0.5	0.5	1.0
5313	20.7	1.3E-04	7.5.E-03	Nt/MT-MT/MT; NtLuc/MT-MT/MT	2.3	1.8	0.8
5506	19.8	1.6E-04	8.0.E-03	Nt/MT-MT/MT; NtLuc/MT-MT/MT	0.3	0.4	1.5
6046	19.7	1.6E-04	8.0.E-03	Nt/MT-MT/MT; NtLuc/MT-MT/MT	0.5	0.6	1.2
6145	26.3	4.1E-05	4.3.E-03	Nt/MT-MT/MT; NtLuc/MT-MT/MT	0.6	0.6	1.0
6146	21.4	1.1E-04	6.9.E-03	Nt/MT-MT/MT; NtLuc/MT-MT/MT	0.6	0.6	1.0
6147	32.9	1.3E-05	1.8.E-03	Nt/MT-MT/MT; NtLuc/MT-MT/MT	0.6	0.6	1.0
6149	20.9	1.2E-04	7.3.E-03	Nt/MT-MT/MT; NtLuc/MT-MT/MT	0.6	0.6	1.0

Table S17. Annotation of the 54 ion peaks statistically significant different abundances among the grafted plant lines by one-way ANOVA.

Ion peak ID	RT[a]	*m/z*[b]	Adduct[c]	Exact mass[d]	Database[e]	Formula[f]
71	3.34	222.098	[M+H]+	221.090	UC2	C8H15NO6
79	3.34	204.087	[M+H]+	203.080	UC2	C8H13O5N
103	3.35	133.032	[M+H]+	132.025	UC2	C5H8O2S
109	3.35	150.059	[M+H]+	149.051	UC2	C5H11NO2S
264	3.72	256.129	[M+H]+	255.122	EX-HR2	C11H17O4N3
337	3.90	163.123	[M+H]+	162.116	UC2	C10H14N2
338	3.91	132.081	[M+H]+	131.074	UC2	C9H9N
339	3.91	130.065	[M+H]+	129.058	UC2	C9H7N
340	3.91	117.058	[M+H]+	116.050	EX-HR2	C4H9N2P1
344	3.92	120.081	[M+H]+	119.074	no hit	Unknown
349	3.97	174.076	[M+H]+	173.069	UC2	C7H11O4N
433	4.32	251.139	[M+H]+	250.132	UC2	C13H18O3N2
453	4.41	221.129	[M+H]+	220.121	EX-HR2	C12H16O2N2
481	4.48	410.112	[M+H]+	409.105	EX-HR2	C18H24O2N3P1S2
498	4.53	86.097	[M+H]+	85.090	no hit	Unknown
501	4.55	132.102	[M+H]+	131.095	UC2	C6H13NO2
524	4.71	179.118	[M+H]+	178.111	UC2	C10H14ON2
565	4.85	177.102	[M+H]+	176.095	UC2	C10H12ON2
588	4.91	398.202	[M+H]+	397.195	EX-HR2	C11H28O5N9P1
596	4.93	86.097	[M+H]+	85.090	no hit	Unknown
597	4.93	132.102	[M+H]+	131.095	UC2	C6H13NO2
986	8.62	457.218	[M+H]+	456.211	EX-HR2	C14H40O6N4S3
1047	8.83	416.119	[M+H]+	415.112	EX-HR2	C10H29O8N3S3
1065	8.90	538.128	[M+H]+	537.120	EX-HR2	C21H28O2N7P1S3
1546	9.72	227.103	[M+H]+	226.095	UC2	C10H14O4N2
1787	10.15	603.190	[M+H]+	602.183	EX-HR2	C26H43N4P1S5
1788	10.15	313.089	[M+Na]+	290.100	UC2	C12H18O8
1789	10.15	329.063	[M+H]+	328.056	EX-HR2	C10H17O10P1
1794	10.15	291.107	[M+H]+	290.100	UC2	C12H18O8
2042	10.64	984.356	[M+H]+	983.349	EX-HR2	C33H87O3N5P2S10
2171	10.85	822.303	[M+H]+	821.296	EX-HR2	C34H70O3N1P7S2
2709	11.65	406.171	[3M+H]+	135.055	UC2	C5H5N5
2816	11.79	137.060	[M+H]+	136.053	UC2	C8H8O2
3287	12.45	335.184	[M+2H]2+	668.353	UC2	C38H52O10
3337	12.52	542.245	[M+H]+	541.238	EX-HR2	C15H39O12N7S1
3343	12.53	547.200	[M+H]+	546.193	EX-HR2	C20H40O9N2P2S1
3520	12.70	629.257	[2M+H]+	314.125	EX-HR2	C11H27O2N2P1S2
3726	12.99	630.278	[2M+H]+	314.635	no hit	Unknown
3872	13.17	375.184	[M+2H]2+	748.354	EX-HR2	C47H49O3N4P1
4131	13.58	490.229	[M+H]+	489.221	EX-HR2	C25H36O3N3P1S1
4137	13.58	189.128	[M+H]+	188.120	UC2	C13H16O
4140	13.58	207.138	[M+H]+	206.131	UC2	C13H18O2
4239	13.72	533.260	[M+H]+	532.253	UC2	C25H40O12
4245	13.73	227.128	[M+H]+	226.121	UC2	C12H18O4
4748	14.51	247.133	[M+H]+	246.126	UC2	C15H18O3

4749	14.51	229.122	[M-H2O+H]+	246.126	UC2	C15H18O3
4935	14.73	334.165	[M+H]+	333.158	UC2	C18H23O5N
5313	15.32	227.164	[M+H]+	226.157	UC2	C13H22O3
5506	15.66	457.207	[M+H]+	456.200	UC2	C22H32O10
6046	17.17	1092.738	[M+4H]4+	4366.923	no hit	Unknown
6145	17.51	229.122	[M-H2O+H]+	246.126	UC2	C15H18O3
6146	17.51	247.133	[M+H]+	246.126	UC2	C15H18O3
6147	17.51	187.112	[M+H]+	186.105	UC2	C13H14O
6149	17.51	265.143	[M+H]+	264.136	UC2	C15H20O4

[a]Mean retention time (min) of the analyte.

[b]Measured *m/z* of the analyte.

[c]Adduct ion of the analyte estimated by considering the mass differences between the ion of interest and all other ions in the corresponding mass spectrum.

[d]Calculated exact mass of the analyte by considering its measured *m/z* and adduct ion.

[e]A database containing the database record which showed minimal error to the calculated exact mass of the analyte. Database search was performed by MFSearcher system using the calculated exact mass of the analyte as a query and the databases, ExactMassDB-HR2 database (EX-HR2) and Unique Connectivity of UnCharged compound database (UC2). In-house standard compound database search was also performed considering the average *m/z*, RT, and MS/MS spectrum of each ion features, but no hit was found.

[f]Chemical formula of the database record which showed minimal error for the calculated exact mass of the analyte.

Table S18 Moisture, protein, fat, ash, carbohydrate and energy of tomato fruits from the three grafted plant lines.

**Sample**[a]	**Moisture**	**Protein**	**Fat**	**Ash**	**Carbohydrate**	**Energy**
	**g/100 g**	**g/100 g**	**g/100 g**	**g/100 g**	**g/100 g**	**kcal/100 g**
MT/MT	91.7	1.5	0.4	0.7	5.7	32
Nt/MT	90.4	1.3	0.5	0.8	7.0	38
NtLuc/MT	90.5	1.5	0.6	0.7	6.7	38

[a]Tomato fruits were sampled from grafted plants produced in 2021. The samples were mixed and used for the analyses to ensure the required weight (30 g) for each of the MT/MT, Nt/MT, and NtLuc/MT groups.
